# Highly Selective Drug-Derived Fluorescent Probes for the Cannabinoid Receptor Type 1 (CB_1_R)

**DOI:** 10.1021/acs.jmedchem.4c00465

**Published:** 2024-07-11

**Authors:** Leonard Mach, Anahid Omran, Jara Bouma, Silke Radetzki, David A. Sykes, Wolfgang Guba, Xiaoting Li, Calvin Höffelmeyer, Axel Hentsch, Thais Gazzi, Yelena Mostinski, Malgorzata Wasinska-Kalwa, Fabio de Molnier, Cas van der Horst, Jens Peter von Kries, Marc Vendrell, Tian Hua, Dmitry B. Veprintsev, Laura H. Heitman, Uwe Grether, Marc Nazare

**Affiliations:** † Leibniz-Forschungsinstitut für Molekulare Pharmakologie (FMP), 13125 Berlin, Germany; ‡ Division of Drug Discovery and Safety, Leiden Academic Centre for Drug Research, Leiden University and Oncode Institute, 2333 CC Leiden, The Netherlands; § Division of Physiology, Pharmacology & Neuroscience, School of Life Sciences, University of Nottingham, NG7 2UH Nottingham, U.K.; ∥ Centre of Membrane Proteins and Receptors (COMPARE), University of Birmingham and University of Nottingham, Edgbaston, B15 2TT Birmingham, Midlands, U.K.; ⊥ Roche Pharma Research & Early Development, Roche Innovation Center Basel, F. Hoffmann-La Roche Ltd., 4070 Basel, Switzerland; # iHuman Institute, ShanghaiTech University, 201210 Shanghai, China; ¶ School of Life Science and Technology, ShanghaiTech University, 201210 Shanghai, China; ∇ IRR Chemistry Hub and Centre for Inflammation Research, Institute for Regeneration and Repair, University of Edinburgh, EH16 4UU Edinburgh, U.K.

## Abstract

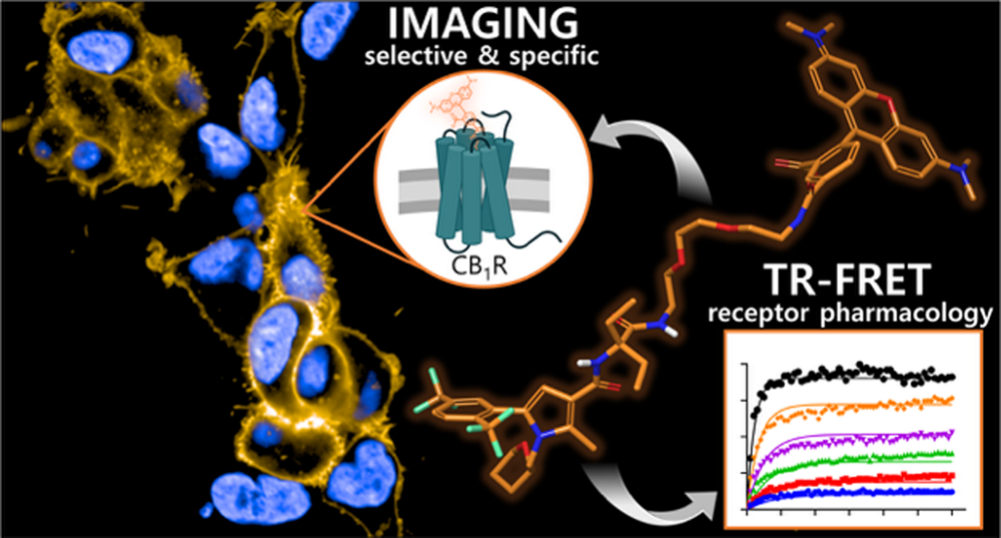

The cannabinoid receptor type 1 (CB_1_R) is pivotal within the endocannabinoid system regulating various signaling cascades with effects in appetite regulation, pain perception, memory formation, and thermoregulation. Still, understanding of CB_1_R’s cellular signaling, distribution, and expression dynamics is very fragmentary. Real-time visualization of CB_1_R is crucial for addressing these questions. Selective drug-like CB_1_R ligands with a defined pharmacological profile were investigated for the construction of CB_1_R fluorescent probes using a reverse design-approach. A modular design concept with a diethyl glycine-based building block as the centerpiece allowed for the straightforward synthesis of novel probe candidates. Validated by computational docking studies, radioligand binding, and cAMP assay, this systematic approach allowed for the identification of novel pyrrole-based CB_1_R fluorescent probes. Application in fluorescence-based target-engagement studies and live cell imaging exemplify the great versatility of the tailored CB_1_R probes for investigating CB_1_R localization, trafficking, pharmacology, and its pathological implications.

## Introduction

Present in all vertebrates, the cannabinoid receptor type 1 (CB_1_R), alongside the cannabinoid receptor type 2 (CB_2_R), is the key signal transducer of the endocannabinoid system (ECS).^1^ CB_1_R is predominantly expressed on presynaptic terminals in the central nervous system (CNS), where it modulates neuronal signaling.^2,3^ Yet, CB_1_R was also found on peripheral cells and organs.^4,5^ In conjunction with its localization, CB_1_R has implications in the homeostasis of various fundamental physiological processes, such as appetite regulation,^6^ energy metabolism,^7^ synaptic plasticity,^8^ and nociception.^9^ Most relevant is the fact that the aberrant expression of CB_1_R is associated with pathophysiological processes, among which are neurodegenerative diseases and neurological, metabolic, and inflammatory disorders.^10,11^ This plethora of potential therapeutic indications underlines the clinical relevance and has triggered extensive pharmaceutical research on CB_1_R.^12,13^ However, the withdrawal of the inverse agonist Rimonabant (**6**, Figure 2) as an anti-obesity agent from the European market in 2008 represented a major incision in CB_1_R drug research.^14,15^ The complexity of ECS signaling and the CB_1_R-related CNS side effects have called for appropriate analytical tools to advance a deeper understanding of the involvement of the CB_1_R in the ECS.^16^ For translation of novel promising CB_1_R drug candidates^17−24^ emerging from preclinical studies to clinical trials, visualization tools for spatiotemporally resolved CB_1_R pharmacological characterization are urgently required.^25^

**Figure 1:**
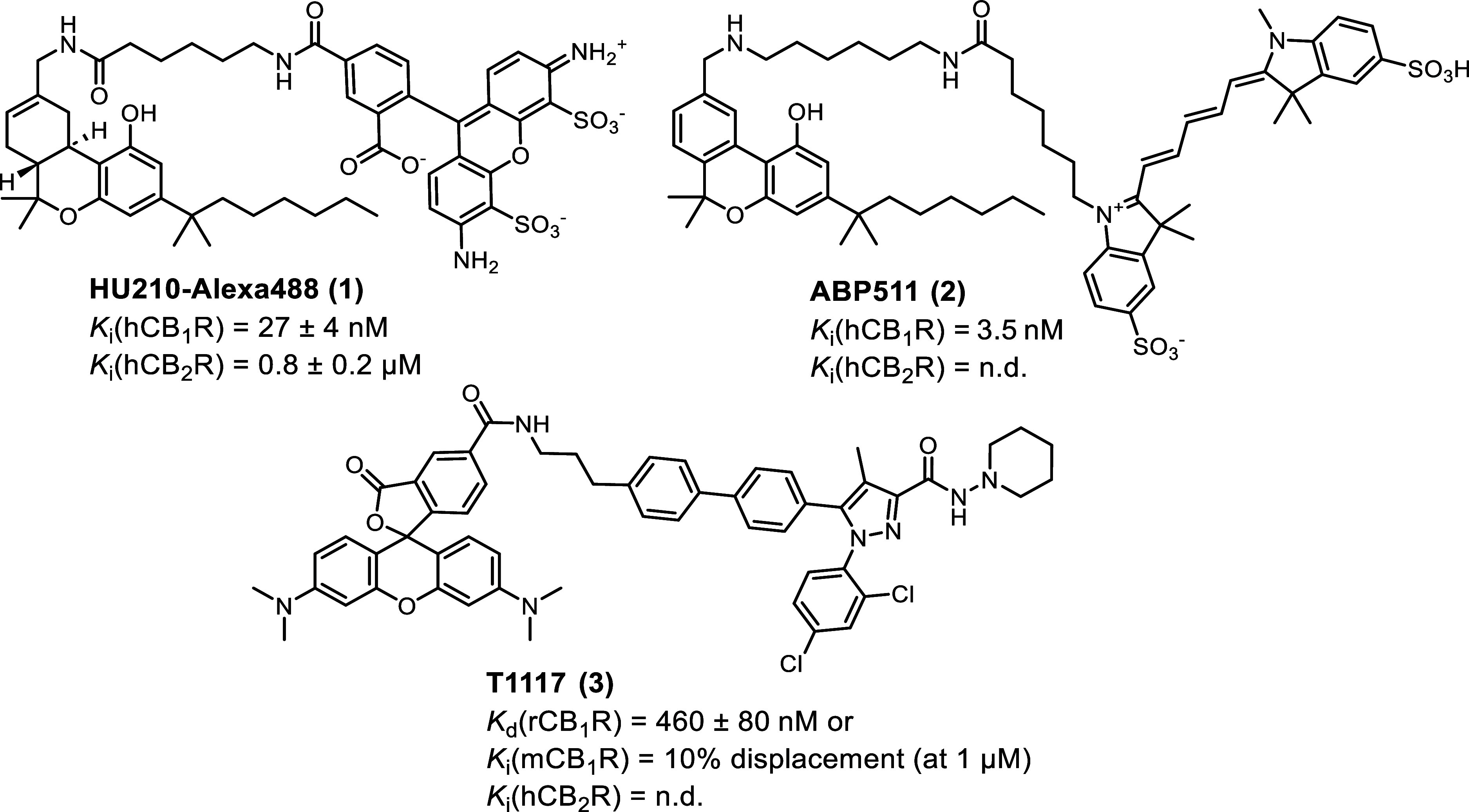
Structures of selected small molecule CB_1_R fluorescent probes.^30,36,37,39^

Fluorescence-based techniques have evolved into a powerful method for studying G-protein coupled receptors (GPCRs).^26,27^ In particular, small molecule fluorescent probes represent versatile tools to elucidate various mechanistic aspects of GPCR pharmacology. Among them are the detection of time-resolved target engagement, allosterism, internalization, dimerization, or membrane organization at the cellular level.^28−33^ While several CB_2_R fluorescent probes were recently reported, only a few CB_1_R fluorescent probes have been described so far.^34,35^ The only two examples of CB_1_R imaging probes are phytocannabinoid-derived (**1** and **2**, Figure 1).^36,37^ In general, issues associated with phytocannabinoid probes are their limited selectivity over CB_2_R and lipophilicity that may result in a high unspecific background signal. Besides phytocannabinoids, synthetic drug-derived fluorescent probes have been reported (e.g., **3**, Figure 1). However, their selective CB_1_R imaging application was not validated further.^30,38,39^ In turn, no CB_1_R-selective imaging probe is described that has been unambiguously characterized pharmacologically in terms of its functional activity and selectivity profile. However, knowledge of the detailed mechanism of action of an imaging probe is crucial to obtain definite and relevant biological results on live cells, as the probe represents a pharmacologically active unit itself. For example, GPCR agonists may induce receptor internalization relevant for internalization studies, whereas an inverse agonist may allow for the detection of steady-state membrane receptor pools.^40^

Herein, we report the modular design, synthesis, pharmacological evaluation, and application of CB_1_R-selective fluorescent probes. The probes were conceptualized based on a reverse design approach employing synthetic drug-like CB_1_R ligands with a defined pharmacological profile as starting points.^41^ By this way, we capitalized for the construction of high-quality tailored labeled probes from drug-derived validated starting points and the analysis and consideration of pre-existent structure-activity relationship (SAR). This study led to the discovery of novel and highly selective pyrrole-based CB_1_R fluorescent probes. Further exploration showcased the versatility of these inverse agonist fluorescent probes for pharmacological time-resolved Förster resonance transfer (TR-FRET) studies and CB_1_R imaging on live cells. Our approach presents a viable design concept for future CBR probes leveraging a deeper understanding of CB_1_R pharmacology.

## Design Concept

Previously, we reported a series of CB_2_R-selective fluorescent probes derived from CB_2_R ligands bearing an α,α-diethyl glycine (DEG) moiety as a versatile and suitable centerpiece for linker attachment.^42−45^ As an amino acid, the DEG motif has granted a high flexibility and synthetic simplicity for amide bond-based derivatization by different pharmacophoric units and linkers achieving CB_2_R probes. Considering the high homology of CB_1_R and CB_2_R, we concluded that this DEG motif would be also a suitable centerpiece in conjunction with a CB_1_R fluorescent probe. Analysis of CB_1_R ligand SAR and ligand alignment studies indicated a strong preference for similar branched lipophilic α,α-diethyl substitutions, providing the necessary steric bulk and favoring distinct conformational preorientation.^46^

**Figure 2:**
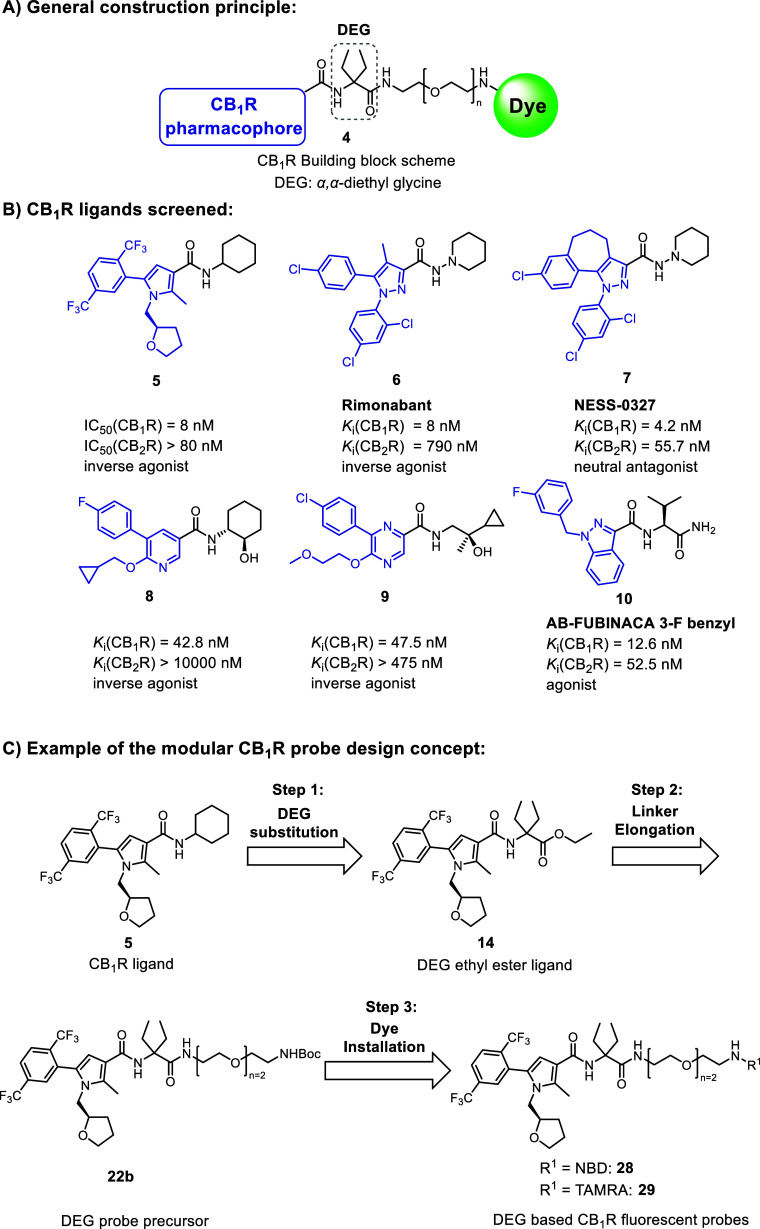
α,α-Diethyl glycine (DEG) amide probe design approach. (A) General construction scheme for CB_1_R fluorescent probes based on DEG. (B) Selected drug-like CB_1_R ligands^47−52^ bearing amide bonds are useful as donors for CB_1_R pharmacophoric units (blue) for the attachment to the DEG centerpiece. Amine fragments (black) were replaced with DEG. (C) Exemplified three-step probe exploration for CB_1_R pyrrole-based fluorescent probes **28** and **29**.

Therefore, we aimed to expand the design scope of this privileged and chemically stable DEG-based probe design toward a CB_1_R probe platform (**4**, Figure 2A). Attempting this, candidate structures as pharmacophore donors were selected among six high-affinity drug-like CB_1_R ligands (**5–10**, Figure 2B). Requirements for selection were a central amide bond to facilitate the attachment to the DEG centerpiece, structural diversity, and varied functionalities, e.g., inverse agonist, antagonist, and agonist.^47−52^

The probe design was based on three exploration steps to achieve validation of our construction concept. We first replaced the original apolar amine unit in **5–10** with DEG ethyl ester to examine whether this modification would be tolerated (Figure 2C). Ideally, the pharmacological properties of the original CB_1_R ligands, such as high affinity, functional activity, and selectivity for CB_1_R, would be preserved upon these structural changes. In the second and third step, the influence of linker attachment and then of fluorescent dye installation was investigated, respectively (Figure 2C). The SAR was screened throughout the series with pharmacological characterization of binding affinity to CB_1_R and CB_2_R.

## Results and Discussion

### Chemistry

**Scheme 1:**
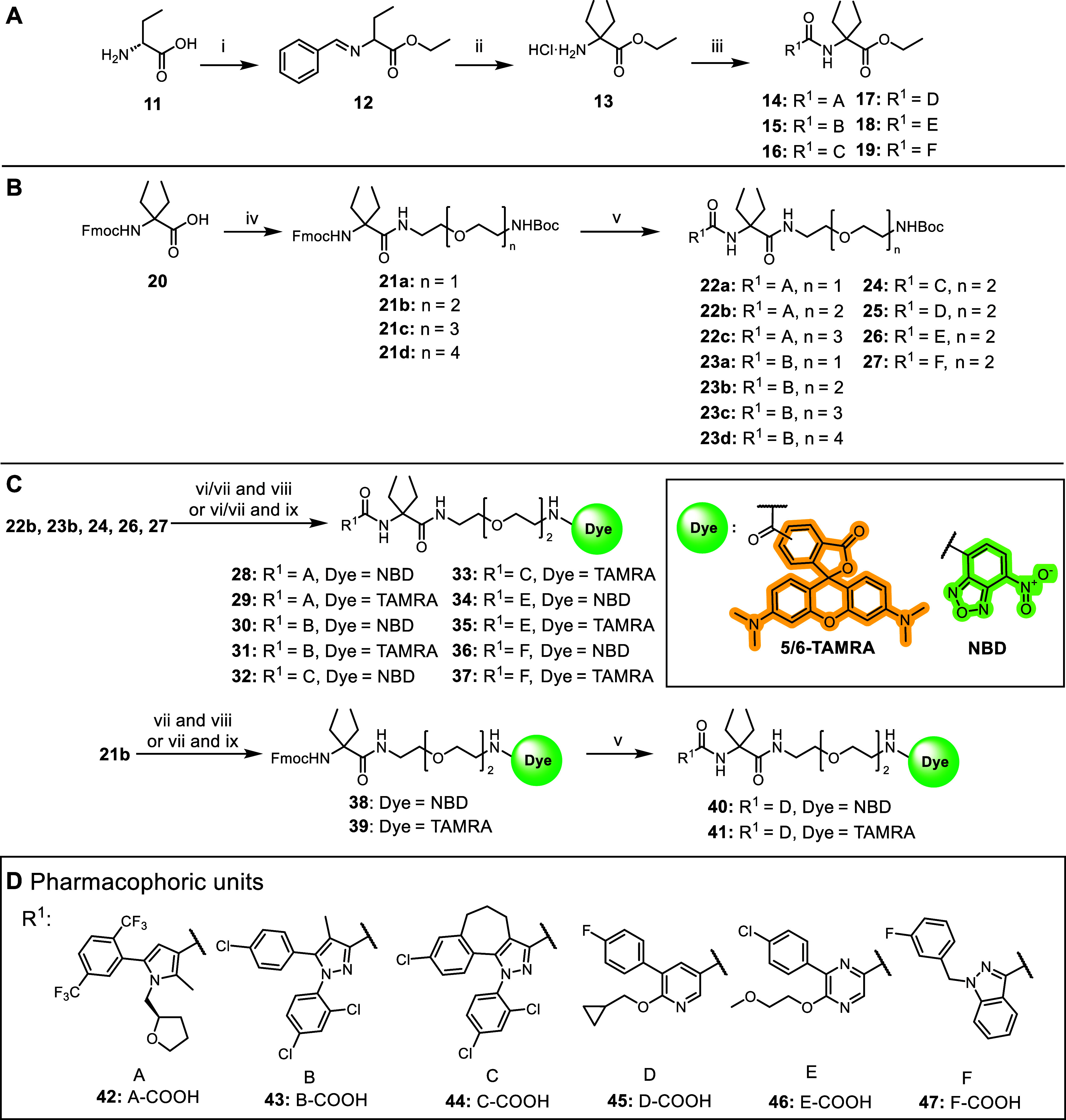
General Synthetic Routes for the Construction of Evaluated Ligands (A) Synthesis of the novel DEG ethyl ester ligands **14–19**: reagents and conditions: (i) (a) SOCl_2_, EtOH, 0 °C to reflux; 5 h; (b) benzaldehyde, TEA, DCM, MgSO_4_, rt, 30 h; (ii) (a) KHDMS, EtI, THF, −70 °C to rt, 24 h; (b) HCl, Et_2_O, 0 °C to rt, 15 h; (iii) **42–47**, HATU, DIPEA, DMF, 3 h, rt. (B) Synthesis of linker library **22a–27**. Reagents and conditions: (iv) HATU, BocNH–CH_2_CH_2_(OCH_2_CH_2_)_*n*_–NH_2_ (*n* = 1–4), DIPEA, DMF, 3 h, rt; (v) DBU, DMF, then HOAt, then **42–47**, HATU, DIPEA, DMF, 3 h 6 d, rt-45 °C. (C) Synthesis of fluorescent probes **28–37**: reagents and conditions: for **28** and **29** (vi) HFIP, MW, 90 min, 150 °C. For **30**, **32**, **34**, **36** (vii) TFA, DCM, 2 h, rt then (viii) NBD-F, DIPEA, DMF, 18 h. (ix) TAMRA-COOH, EDCI, HOAt, DIPEA, DMF, 20 h, rt or TAMRA-SE, DIPEA, DMF, 2 h, rt. Synthesis of fluorescent probes **40–41**: reagents and conditions: (vii) TFA, DCM, 2 h, rt. (viii) NBD-F, DIPEA, DMF, 18 h. (ix) TAMRA-COOH, EDCI, HOAt, DIPEA, DMF, 20 h, rt or TAMRA-SE, DIPEA, DMF, 2 h, rt. (v) DBU, DMF, then HOAt, then **45**, HATU, DIPEA, DMF, 3 h, rt. (D) Pharmacophoric carboxylic acid units **42–47** derived from **5–10**. For synthesis of probes **51** and **52,** see the Experimental Section.^54^

The synthesis of the novel DEG ethyl ester ligands **14–19** is outlined in Scheme 1A. The synthesis began with SOCl_2_-facilitated esterification of the carboxylic acid functional group of **11,** followed by protection of the amino group to give benzylidene intermediate **12**. The central DEG building block **13** was obtained via an alkylation of **12** using ethyl iodide and KHDMS, followed by hydrolysis of the benzylidene imine under acidic conditions. HATU-mediated amide coupling reaction with respective carboxylic acids **42–47** furnished the desired DEG ligands **14–19**.

To determine the optimal linker length for the fluorescent dye attachment, commercially available *N*-Fmoc-α,α-diethyl glycine **20** was utilized (Scheme 1B). Using an orthogonal protecting group strategy, a series of *N*-Boc protected diamine linkers (*n* = 1–4) were coupled to amino acid **20** using HATU to give access to **21a–d**. Fmoc-protecting group removal of compounds **21a–d** using DBU was followed by in situ coupling to corresponding carboxylic acids **42–47** to afford Boc-protected congeners **22a–c**, **23a–d**, and **24–27**. Notably, the HATU coupling of **42** with Fmoc-deprotected **22a–c** resulted in consistently low yields with an unreactive HOAt-ester intermediate as the main product (**S53**, see Figure S24). This observation could be attributed to the steric hindrance of DEG, which is known to be a challenging factor in amide couplings.^53^ The initially observed low yields of <10% for the amide coupling reaction (see **22a**) were improved for **22b** and **22c** by increasing the temperature to 40–45 °C and prolongation of the reaction times to 4–7 days (56 and 48% yield, respectively).

To obtain target fluorescent probes **28–37** and **40–41**, the terminal *N*-Boc protecting group of **-22b**, **23b**, and **24–27** had to be removed (Scheme 1C). Cleavage using TFA was applied for **23b**, **24**, **26,** and **27**. This procedure, however, was not compatible with compounds **22b** and **25** where partial degradation in the presence of TFA was observed. To overcome this problem, Boc-deprotection of **22b** was performed under mild, microwave-assisted cleavage using 1,1,1,3,3,3-hexafluoroisopropanol (HFIP).^55^ This procedure was found to be mild enough to avoid decomposition and yielded the free terminal amine of **22b**. The resulting free amines were coupled either to carboxy 5/6-tetramethyl rhodamine (TAMRA) fluorescent dye by amide coupling or to fluoro-nitrobenzoxadiazole (F-NBD) via nucleophilic aromatic substitution conditions to achieve probes **28**–**37** (see Figures S25–S34). Boc-deprotection of **25** was possible neither with TFA nor under HFIP/MW conditions. Therefore, probes **40** and **41** were synthesized via a variation of the synthetic route starting with Boc-deprotection of **21b** followed by conjugation of fluorescent dyes to obtain intermediates **38** and **39**. After the removal of the Fmoc-protecting group with DBU, another amide coupling under HATU conditions gave access to the fluorescent probes **40** and **41** (Scheme 1C, see Figures S35 and S36).

### Computational Studies

**Figure 3:**
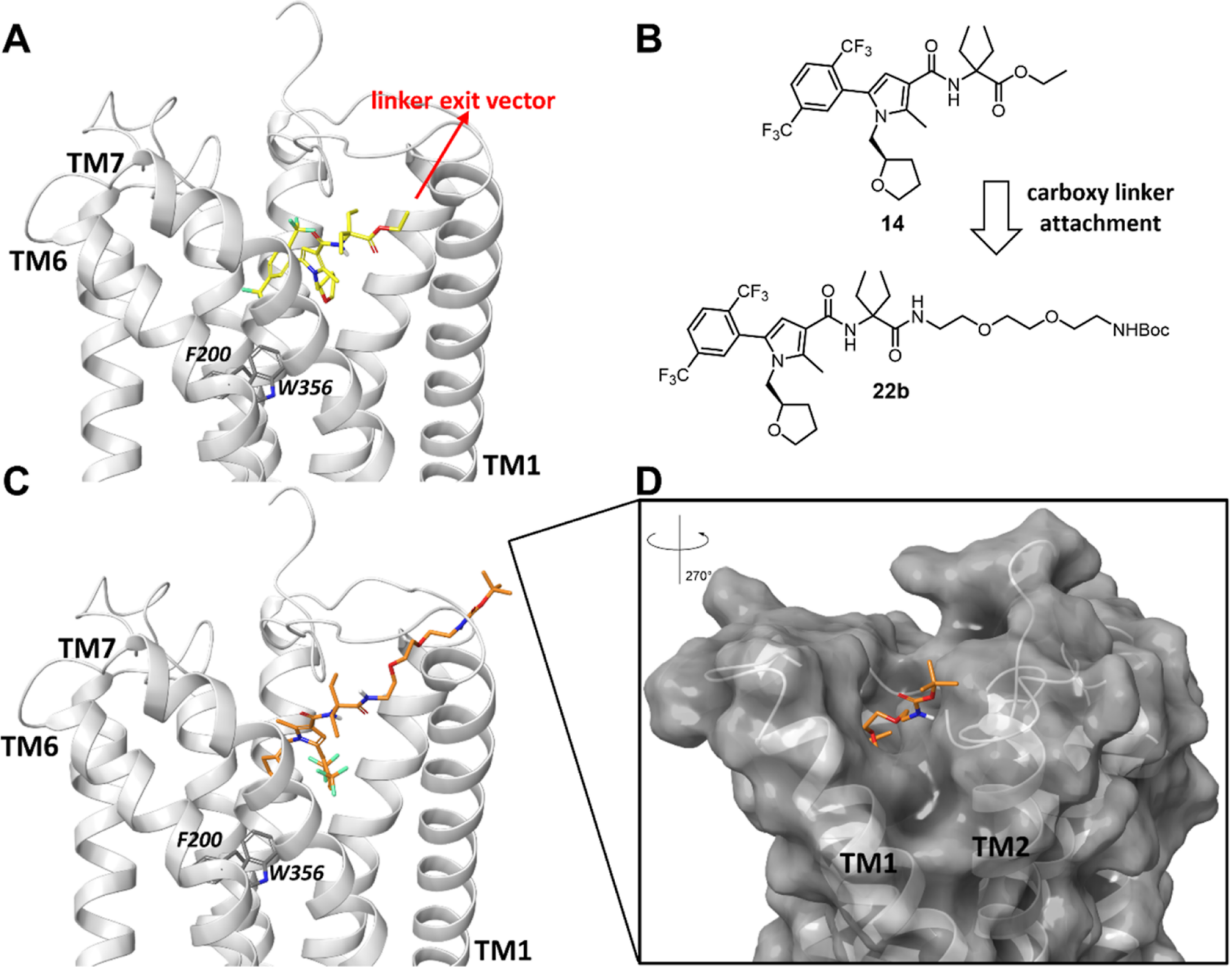
Docking poses of representative pyrrole-based CB_1_R ligand **14** and DEG probe precursor **22b** in the CB_1_R inactive state (light gray, docked in PDB ID: 5TGZ, X-ray diffraction, 2.80 Å).^56^ (A) Docking pose of novel DEG ethyl ester ligand **14** (bright yellow) located in the binding pocket of CB_1_R with the ethyl ester group pointing toward the N-terminal site. (B) Linker installation on **14** via the carboxy-terminal amide bond of DEG is a reasonable strategy based on the docking structures. (C) DEG probe precursor **22b** with *n* = 2 (orange). (D) Linker reaches the CB_1_R extracellular site through trans-membrane helices (TM) 1 and 2. For docking poses of **15–19**, **23b**, and **24–27** and a detailed description of the docking studies, see Supporting Information.

Docking studies were conducted to explore the orientation of novel DEG ethyl ester ligands **14–19**. Exemplified in Figure 3A is the docking structure of the DEG ethyl ester **14** derived from **5** in inactive CB_1_R (utilizing PDB ID: 5TGZ).^56^ Interestingly, the pharmacophoric pyrrole unit in **14** bearing the DEG centerpiece unit was well accommodated in the binding pocket of CB_1_R aligning with the known cocrystallized ligand AM6538 (PDB ID: 5TGZ) (see Figure S14). The DEG unit in **14** was oriented toward the extracellular space comparable with the piperidine unit of AM6538. Docking poses of compounds **15**–**19** consistently showed that the ethyl ester moiety points toward the *N*-terminus of CB_1_R (see Supporting Information, Figures S9–S13) and that the α-ethyl side chains are favorably involved in attractive van der Waals interactions with the F174 side chain. We therefore concluded that DEG can favorably replace the original amine units of **5–10** (Figure 2B, black fragments). Hence, utilization of the CB_1_R pharmacophoric units from **5–10** in conjunction with a DEG centerpiece appeared as a promising approach toward a platform for CB_1_R fluorescent probes. In addition, the docking study revealed that the terminal carboxy group of DEG is an ideal linker attachment site, allowing free access to the extracellular space, thereby avoiding extensive linker attachment studies (Figure 3A,B).

To estimate a proper linker length for fluorescent dye attachment in our probes, docking studies were performed on compounds **22b**, **23b**, and **24–27** in the same receptor structure. The docking pose of **22b** is shown in Figure 3C (for compounds **23b** and **24–27,** see Figures S9–S13). The PEG chain with *n* = 2 was predicted to reach out to the CB_1_R extracellular site through the trans-membrane helices TM1 and 2. This linker appeared to be long enough to allow for the envisioned fluorescent dye attachment at the terminal amine without interfering with binding (Figure 3D). A detailed SAR investigation on the linker length confirmed these results (see the next section).

### In Vitro Pharmacology

#### Pharmacological Profiling of DEG Ethyl Ester Intermediates

We first analyzed the novel drug-like DEG ester-derived CB_1_R ligands **14–19** to experimentally examine whether the insertion of DEG moiety would be tolerated without compromising CB_1_R affinity and functional activity compared to the original counterparts **5–10** (Table 1, binding data with standard error of mean in Table S1). The binding affinities were measured in a radioligand binding assay on Chinese hamster ovary (CHO) membranes stably expressing either human CB_1_R or CB_2_R. In this assay, all compounds (**14–19**) exhibited nanomolar to submicromolar affinity for human CB_1_R. However, among all tested chemotypes, only **14** preserved CB_1_R affinity and showed marked selectivity for CB_1_R [(*K*_i_(CB_1_R) = 11 nM; *K*_i_(CB_2_R) = 306 nM, *K*_i_(CB_2_R)/*K*_i_(CB_1_R) = 28-fold selectivity]. Notably, compounds **17** and **18** showed a swap from CB_1_R-selectivity to CB_2_R-selectivity. This finding could be attributed to the acquired structural similarity to 3,4,5-substituted pyridine CB_2_R-ligands^43^ upon conjugation with the DEG ethyl ester. Even though the differences between the CB_1_R and CB_2_R binding affinities for compounds **15** and **16** were not pronounced, they exhibited a slight preference for CB_2_R. Even though indazole-based **19** showed no CB_1_R-selectivity after installation of the DEG moiety, the lack of CB_1_R-selectivity was not surprising in this case as **19** was derived from agonist **10**, which already featured a weak CB_1_R preference [*K*_i_(CB_2_R)/*K*_i_(CB_1_R) = 4-fold selectivity] commonly observed with this compound class.^52,57^ In a CB_1_R cAMP functional homogeneous time-resolved fluorescence (HTRF) assay,^58^**14**, **15**, **17**, and **18** were found to be inverse agonists and **19** an agonist, while **16** showed no activity in the assay. To our delight, all DEG esters retained the functional activity of their original structures **5–10**.

**Table 1:**
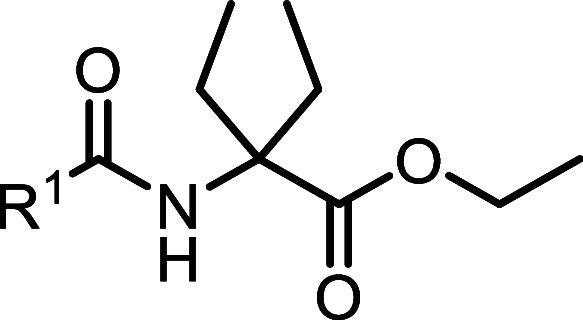

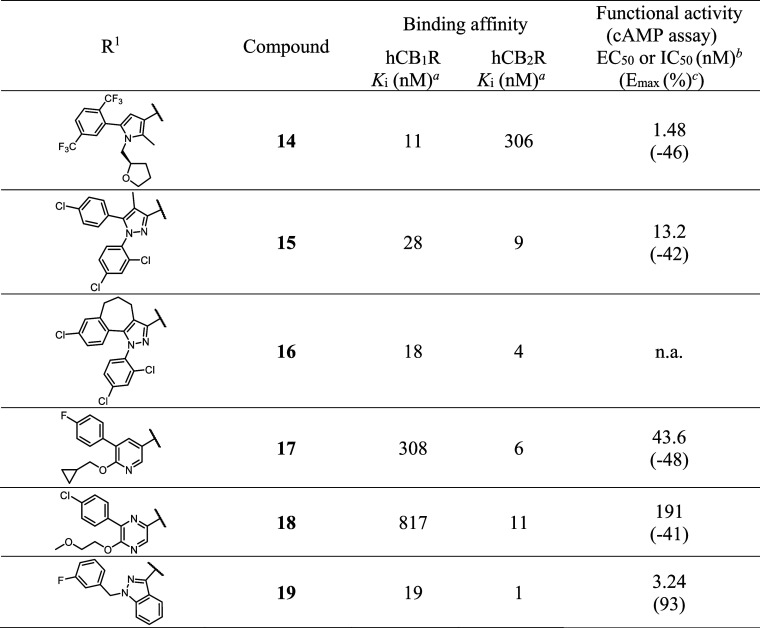
Binding Affinities and Functional Activity of CB_1_R DEG Ligands

a *K*_i_ (nM) values obtained from [^3^H]CP55,940 displacement assays on CHO membranes stably expressing human CB_1_R or human CB_2_R. Values are means of three independent experiments performed in duplicate.

b The activity levels (EC_50_ or IC_50_) of **14**–**19** were measured using cells stably expressing hCB_1_R in homogeneous time-resolved fluorescence (HTRF) cAMP assay. The data are the means of three independent experiments performed in technical replicates.

c Maximum effect (*E*_max_ in %) was normalized to reference full agonist CP55,940. n.a. denotes no activity. All data with standard error of mean are given in the Supporting Information.

Previous studies showed that linker and fluorescent dye attachment can strongly affect the pharmacological profile of probes in an unpredictable fashion.^59−61^ Hence, to get an unbiased and detailed picture of the linker tolerance of the structures and optimal length for fluorescent dye conjugation, we progressed with a linker screen by using compound series **22a–c** and **23a–d** with *N*-Boc-protected terminus as model compounds.

The pharmacological evaluation of the DEG probe precursors **22a–c**, **23a–d**, and **24–27** is outlined in Table 2 (binding data with standard error of mean in Table S1). Even though the overall binding affinities of compounds **22a–c** declined compared to **14**, CB_1_R preference was preserved. Despite the absence of a linear correlation between the linker length and binding affinities, linker length *n* = 2 of **22b** appeared as most favorable, as it exhibited the highest CB_1_R affinity and selectivity. In addition, **22b** retained inverse agonist activity (IC_50_ = 131 nM, *E*_max_ = −69%). This linker selection was supported by our docking studies (Figure 3). We further examined the effect of the linker attachment and length on pyrazoles **23a–d** compared to DEG ethyl ester ligand **15**. Interestingly, while attachment of DEG ester in compound **15** attenuated its CB_1_R selectivity, installation of *N*-Boc-protected PEG chains in compounds **23a–d** revived the CB_1_R-selectivity over CB_2_R. Unlike **22a–c**, compounds **23a–d** showed a linear correlation between CB_1_R affinity and the linker lengths. In this series, **23a** (*n* = 1) showed the highest affinity and selectivity to CB_1_R. However, a short linker might lead to a steric clash with the receptor’s binding pocket after the envisioned fluorescent dye installation and consequently compromise binding affinity. Altogether, the molecular docking of **22b** and binding data of series **22a–c** and **23a–d** supported the selection of *n* = 2 as the most suited linker for our probes. To our delight, **23b** also showed conserved functional activity as an inverse agonist on CB_1_R (IC_50_ = 64.6 nM, *E*_max_ = −44%).

**Table 2:**
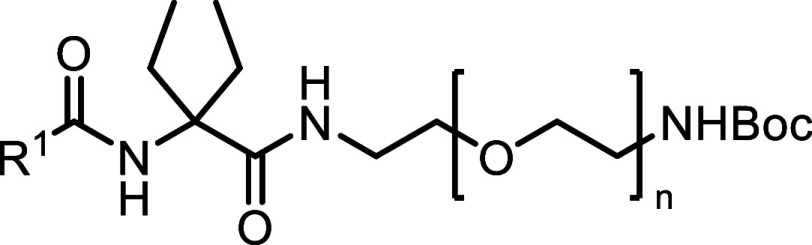

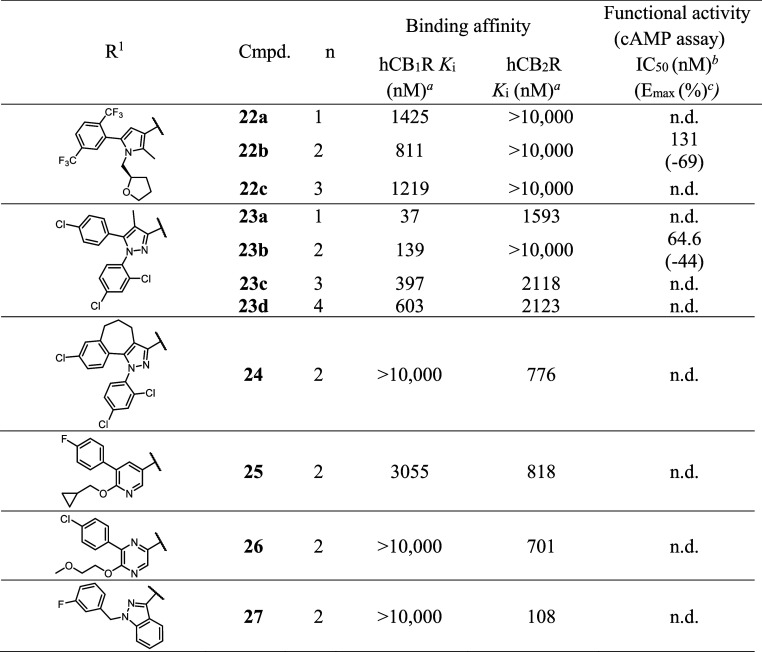
Binding Affinities and Functional Activity of the N-Boc-Protected DEG Probe Precursors

a *K*_i_ (nM) values obtained from [^3^H]CP55,940 displacement assays on CHO cell membranes stably expressing human CB_1_R or human CB_2_R. Values are means of three independent experiments performed in duplicate.

b The activity levels (IC_50_) of **22b** and **23b** were measured using cells stably expressing hCB_1_R in homogeneous time-resolved fluorescence (HTRF) cAMP assay. The data are the means of three independent experiments performed in technical replicates.

c Maximum effect (*E*_max_ in %) was normalized to reference full agonist CP55,940. n.d. is not determined. All data with standard error of mean are given in the Supporting Information.

The *N*-Boc-protected PEG chain with *n* = 2, as the ideal linker, was also examined in combination with pharmacophores **44–47** yielding DEG probe precursors **24–27**. Unfortunately, compounds **24–27** exhibited no or significantly weaker CB_1_R binding (between 3 and >10 μM) (Table 2) and instead CB_2_R preference indicating that linker elongation is not equally well tolerated by all pharmacophores.

#### Pharmacological Profiling of CB_1_R Fluorescent Probes

Next, we studied the CB_1_R binding affinity of probes **28–37** and **40–41** equipped with fluorescent dyes NBD and TAMRA. As the presence of a fluorescent dye might significantly alter the pharmacological profile of the probes,^59,62,63^ we thoroughly characterized our target compounds (Table 3, binding data with standard error of mean in Table S1). In this study, we have chosen green-emitting NBD and orange-emitting TAMRA as examples for sterically small and large fluorescent dye, respectively. In addition, TAMRA as a partially zwitterionic hydrophilic rhodamine-derivative should be especially suited for cellular imaging of membrane proteins due to its good photostability and quantum yield. Photophysical characteristics of the probes were assessed in PBS buffer (Table S5). We determined the CB_1_R and CB_2_R binding profile of the labeled probes carrying different fluorescent dyes in the radioligand binding assay and in the functional HTRF cAMP assay. We observed fluorescent dye-dependent differences in the binding profile of the probes. For example, pyrrole-based probes **28** and **29** bearing NBD and TAMRA, respectively, maintained their CB_1_R-selectivity. However, the substantially lower *K*_i_ value for the TAMRA probe [**29**, *K*_i_(hCB_1_R) = 2077 nM, *K*_i_(CB_2_R)/*K*_i_(CB_1_R) > 4.8] suggested that the larger TAMRA dye might interfere with ligand binding, while NBD conjugation turned out to be beneficial for the CB_1_R affinity [**28**, *K*_i_(hCB_1_R) = 97 nM, *K*_i_(CB_2_R)/*K*_i_(CB_1_R) > 103] when compared to the DEG probe precursor **22b** (*K*_i_(hCB_1_R) = 811 nM). In contrast to the binding assay, both inverse agonists **28** (IC_50_ = 16.6 nM) and **29** (IC_50_ = 102 nM) were more potent in the cAMP functional assay when compared to **22b** and with only weak fluorescent dye-dependency. A similar effect was observed for pyrazole-based probes **30** and **31**. Inverse agonist NBD probe **30** [*K*_i_(CB_1_R) = 428 nM; *K*_i_(CB_2_R)/*K*_i_(CB_1_R) > 23, IC_50_ = 60.3 nM] preserved its CB_1_R profile when compared to precursor **23b** while TAMRA conjugation was deleterious for the binding affinity of **40** to either of the CBRs. To our surprise, the indazole-based NBD probe **36** showed binding to CB_1_R [*K*_i_(CB_1_R) = 1174 nM], while its DEG probe precursor **27** and TAMRA congener **37** were solely CB_2_R binders. Yet, both showed preferred binding to CB_2_R. Similarly, rigidified pyrazole **32**, pyridine **40,** and pyrazine **34** NBD probes displayed CB_2_R selectivity. Within this series, fluorescent dye dependency was observed again, as with their respective TAMRA congeners, **33**, **41**, and **35** did not bind to either of the receptors.

**Table 3:**
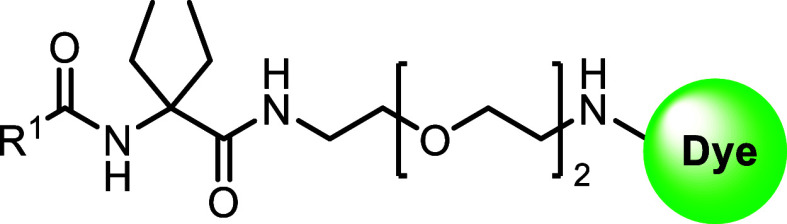

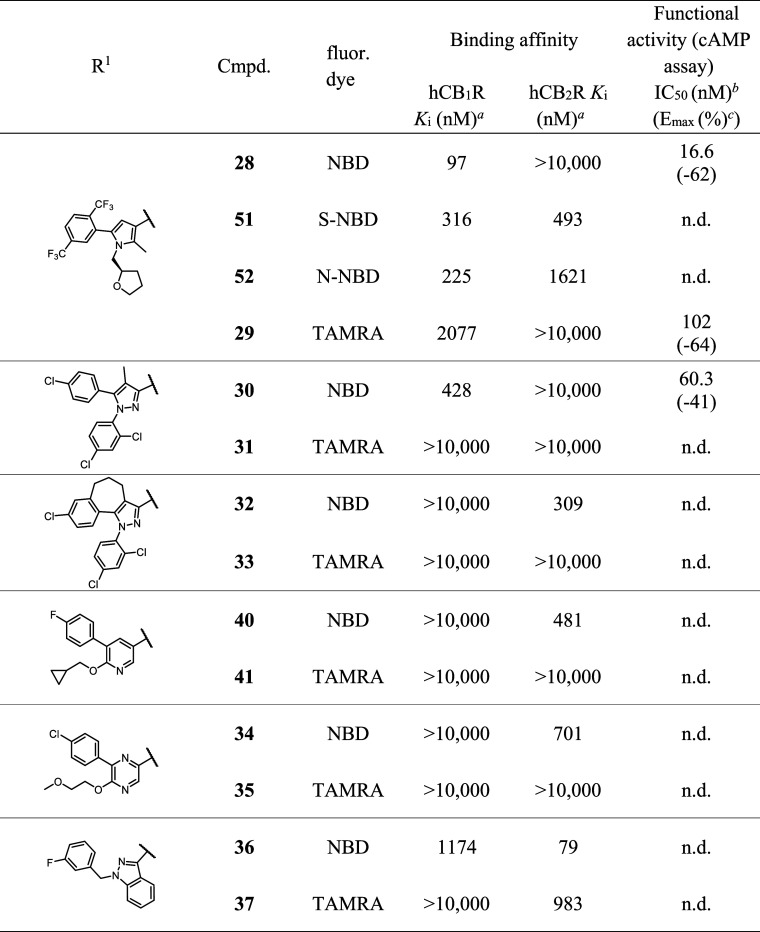
Binding Affinities and Functional Activity of the Fluorescent Probes

a *K*_i_ (nM) values obtained from [^3^H]CP55,940 displacement assays on CHO membranes stably expressing human CB_1_R or human CB_2_R. Values are means of three independent experiments performed in duplicate.

b The activity levels (IC_50_) of **28–30** were measured using cells stably expressing hCB_1_R in homogeneous time-resolved fluorescence (HTRF) cAMP assay. The data are the means of three independent experiments performed in technical replicates.

c Maximum effect (*E*_max_ in %) was normalized to reference full agonist CP55,940. n.d. is not determined. All data with standard error of mean are given in the Supporting Information.

In summary, all NBD probes maintained CBR preference as observed with their corresponding DEG probe precursor structures in the linker screen (Table 2). In turn, installation of the sterically more demanding TAMRA dye was not tolerated well and led to a loss of CB_1_R binding affinity except for probe **29**. This trend was further confirmed with two other small fluorescent dyes of the “Scotfluor” series^54^ (CB_1_R-selective probes **51** and **52**, for synthesis, see Experimental Section). Our investigation exemplifies that the modular reverse design approach is capable of facilitating and guiding the construction of DEG-based fluorescent probes from CB_1_R pharmacophores but that careful pharmacological characterization is crucial for probe design.

#### Conformational Molecular Dynamics Simulation

**Figure 4:**
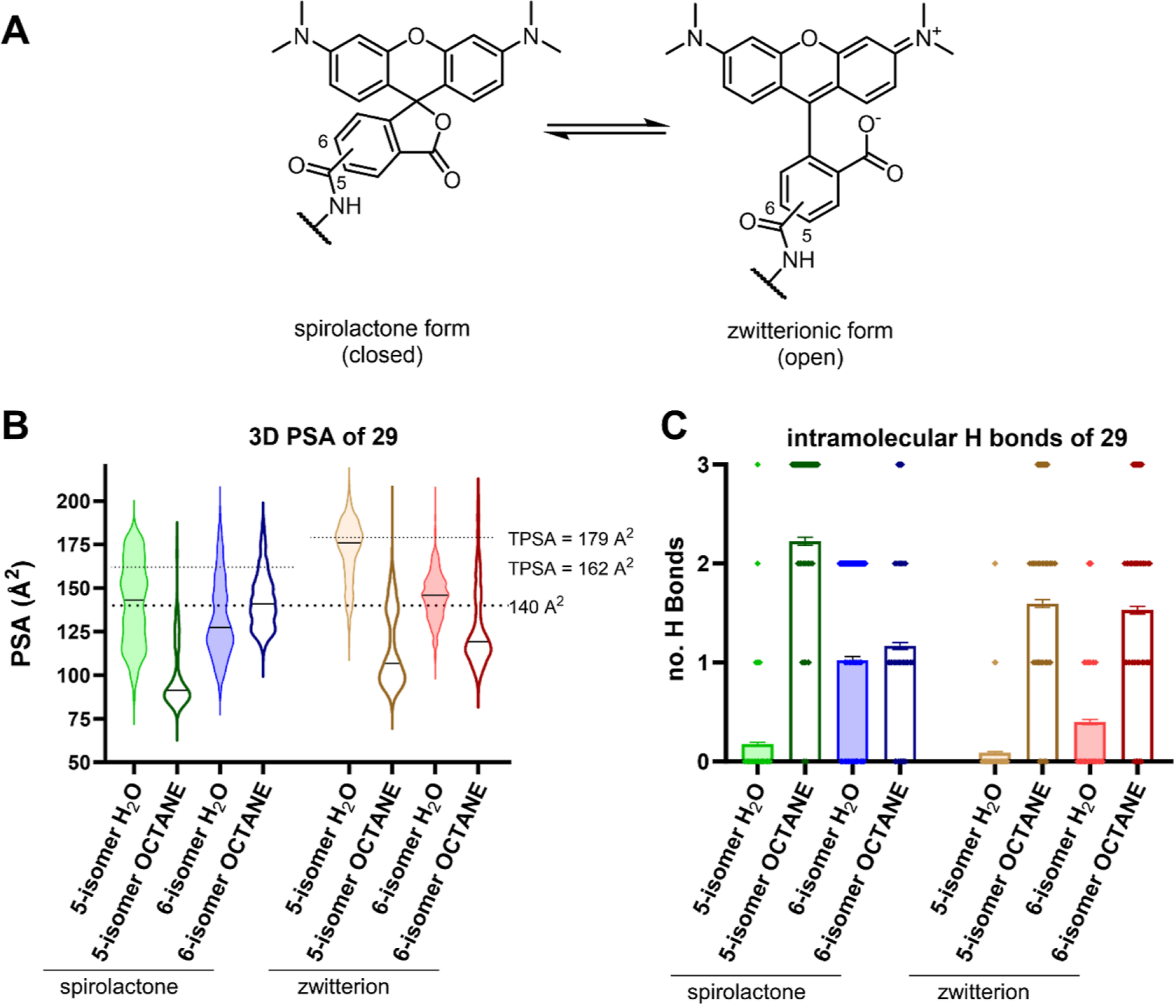
Conformational analysis of **29**. (A) Equilibrium of 5/6-TAMRA isomers as spirolactone (closed) and zwitterionic (open) form. (B) Violin plot of the 3D PSA distribution of the four possible isomers (open/closed, 5/6-isomer) of **29** in water and octane obtained by MD simulation (50 ns). Drug-like PSA cutoff 140 Å^2^ and tPSA of spirolactone and zwitterion form are indicated as dotted lines. Mean 3D PSA of each isomer is indicated as black line in the violin plot. (C) Intramolecular hydrogen bond formation observed in MD simulation (50 ns) of the four possible isomers (open/closed, 5/6-isomer) of **29** in water and octane. Mean hydrogen bond interactions represented as bar chart ± SEM.

While the classical construction principle of fluorescent dye labeled probes features several physicochemical characteristics that might hamper passive cellular permeation, we still observed rather efficient permeation at low concentration of probe **29** in the confocal imaging experiment (vide infra). Specifically, **29** has a high molecular weight, an increased number of rotatable bonds, and a high topological polar surface area (tPSA) and is equipped with 5/6-TAMRA, which equilibrates in an (open) zwitterionic or a (closed) spirolactone form (Table 4 and Figure 4A).^64−67^ We therefore investigated the unexpected membrane permeability of **29** by molecular dynamics (MD) simulations. For that, we hypothesized that **29** would effectively reduce its critically high PSA of >140 Å^2^ by formation of intramolecular hydrogen bonds (IMHB) when entering an apolar environment (“chameleonic effect”).^68−70^ During a 50 ns MD simulation, we analyzed the conformations of **29**, their 3D PSA, and the amount of formed IMHBs in water and *n*-octane (as a model of apolar cell membrane environment).

**Table 4:** Calculated Physicochemical Descriptors of DEG Ethyl Ester Ligand **14** and Probe **29** Isomers as Spirolactone and Zwitterion Forms by Chemoinformatic Tools

compd.	MW (g/mol)^a^	HBA^a^	HBD^a^	rotatable bonds^a^	tPSA (Å^2^)^a^
**14**	562.54	10	1	13	69.56
5/6-**29** spirolactone	1077.12	15	3	25	161.93
5/6-**29** zwitterion	1077.12	15	3	26	179.44

a SwissADME.ch prediction by Swiss Institute of Bioinformatics.^71^

Probe **29** adopted a broad range of conformations with variable 3D PSA (see Figures 4B and S15–S22). Consistently, the transition of compound **29** from water to *n*-octane would lead to a significant reduction of the mean 3D PSA and an increased number of IMHBs interactions, with the only exception being the spirolactone 6-isomer. For instance, the mean 3D PSA of the 5-zwitterion isomer (prevalent in water) would be reduced from 171.7 to 99.8 Å^2^ when transitioning into *n*-octane and equilibrating into the spirolactone form (prevalent in apolar solvents). Simultaneously, the mean number IMHB of 0.1 in water would increase to 2.2 in *n*-octane (for other values see Table S4).

These MD data suggest that in particular, the 5-isomer of **29** has a strong tendency for chameleonic effects. In addition, based on the 3D PSA, a better membrane permeability by passive diffusion of probe **29** can be concluded than predicted by classical metrics of drug-likeness.^71^ This shows that MD-derived studies for assessment of intracellular accessibility of high molecular weight compounds are relevant and useful also for fluorescent probe conjugates.^68,72^

#### TR-FRET Binding Assay

TR-FRET has evolved as an attractive alternative to radioligand binding assays using fluorescent probes as tracers. TR-FRET assays are available for CB_1_R^73−75^ and especially suited for the determination of kinetic ligand–receptor interactions.^76,77^ Consequently, human embryonic kidney (HEK293TR) cells overexpressing SNAP-tagged hCB_1_R were labeled with a SNAP-Lumi4-Tb FRET-donor and cell membrane preparations generated. Laser excitation of the terbium cryptate (337 nm) on the N-terminus of CB_1_R induces energy transfer to a fluorescent probe when bound to CB_1_R.

**Figure 5:**
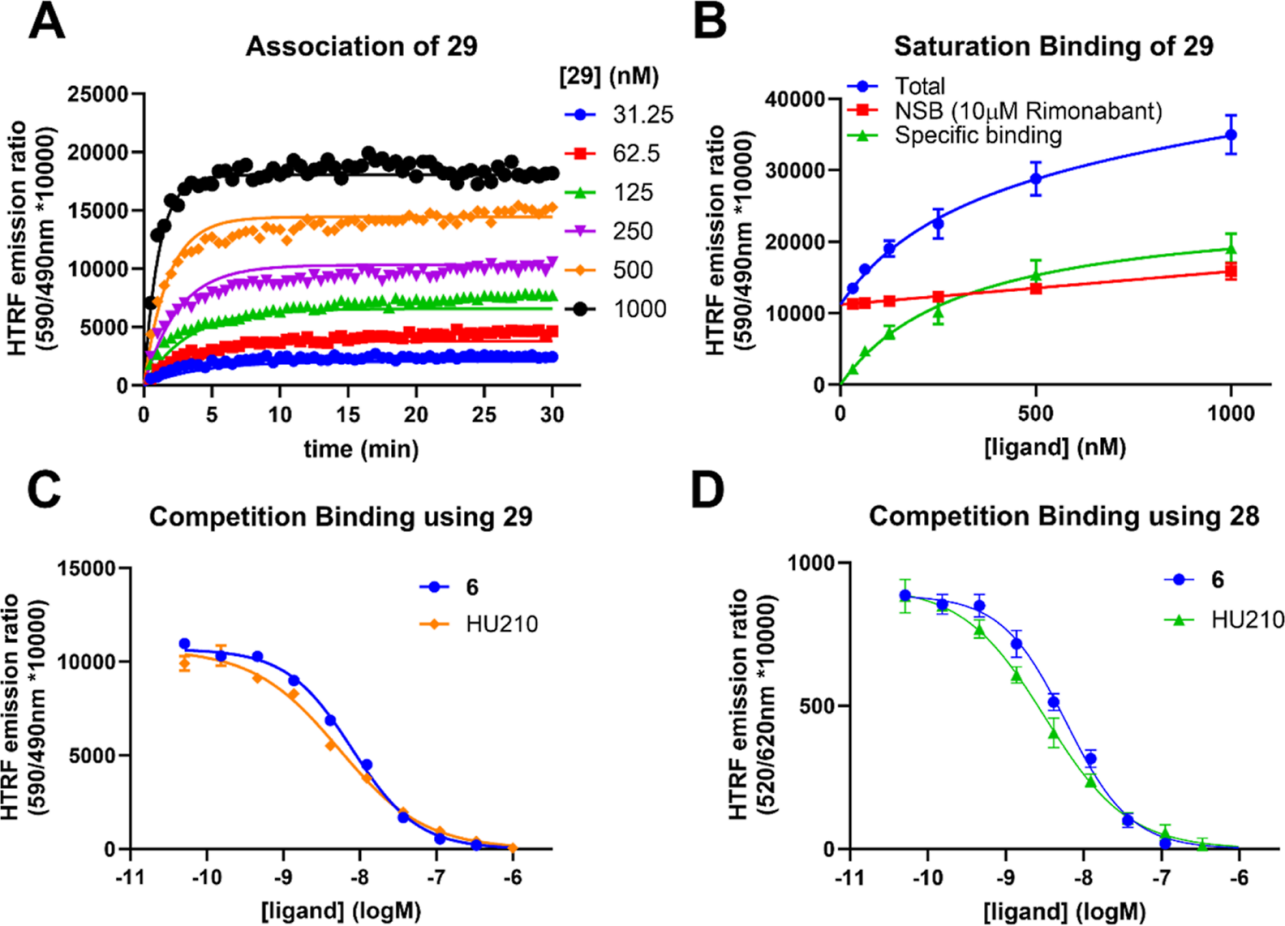
TR-FRET binding assays using HEK293TR-hCB_1_R cell membranes. (A) Observed association curves of TAMRA probe **29** to hCB_1_R. (B) Saturation binding analysis of **29** to hCB_1_R after 60 min. (C) Competition binding using **29** (300 nM) as a tracer with increasing concentrations of CB_1_R ligand **6** and HU210. (D) Competition binding using NBD probe **28** (60 nM) as a tracer with increasing concentrations of CB_1_R ligand **6** and HU210. Kinetic and equilibrium data were fitted to the equations described in the Supporting Information to calculate *K*_D_, *k*_on_, and *k*_off_ values for fluorescent and unlabeled ligands. Data are presented as mean ± SEM, *N* = 3.

We first examined saturation and kinetic binding parameters of TAMRA probe **29** on CB_1_R membrane preparations. The probe showed stable binding to the receptor over a time course of 30 min (Figure 5A). The saturation binding affinity value of **29** was lower (*K*_D_ = 335.5 nM) (Figure 5B) than obtained in the radioligand binding assay, yet, still in a commensurate range. In a kinetic association and dissociation experiment, **29** exhibited a moderate association rate of 0.81 × 10^6^ M^–1^ min^–1^ on hCB_1_R which supports its applicability as imaging probe (Table 5).

**Table 5:** HTRF Binding Parameters of CB_1_R Probe **29** and Unlabeled Ligands^a^

compd.	*k*_on_ (10^6^ M^–1^ min^–1^)	*k*_off_ (min^–1^)	RT (min)	kinetic *K*_D_ (nM)	*K*_i_ (nM)^b^	*K*_i_ (nM)^c^
**29**	0.81	0.26	3.85	365		
**6**	48.3^b^	0.15^b^	6.67	3.23	3.34	2.08
HU210	37.4^b^	0.11^b^	9.10	3.04	2.08	1.26

a Data are presented as mean, *N* = 3.

b Probe **29** (300 nM) used as a tracer.

c Probe **28** (60 nM) used as a tracer. RT: residence time. All data with standard error of mean are given in the Supporting Information.

Exploring the binding kinetics of a ligand is a crucial aspect of GPCR drug development and can be used to promote improved drug efficacy.^78^ Using **29** as a fluorescent tracer, the kinetic parameters *k*_on_ and *k*_off_ and resulting *K*_D_ of **6** and HU210 were determined (Table 5). In addition, their equilibrium binding affinity was determined with both fluorescent NBD **28** and TAMRA **29** as tracers in a simple competition binding assay (Figure 5C,D and Table 5). Competition binding affinities *K*_i_ of the known CB_1_R ligands were in excellent agreement with the kinetic *K*_D_ and literature radioligand binding affinities determined at human CB_1_R.^79−82^ In addition, the determined *K*_i_ values of **6** and HU210 were probe-independent. These experiments underscore the applicability of our fluorescent pyrazole probes **28** and **29** as highly useful tools in TR-FRET-based CB_1_R drug discovery to characterize the kinetic binding and equilibrium affinities of CB_1_R ligands in a potential high throughput setting avoiding radioactively labeled ligands.

#### Fluorescence Confocal Microscopy in Live Cells

**Figure 6:**
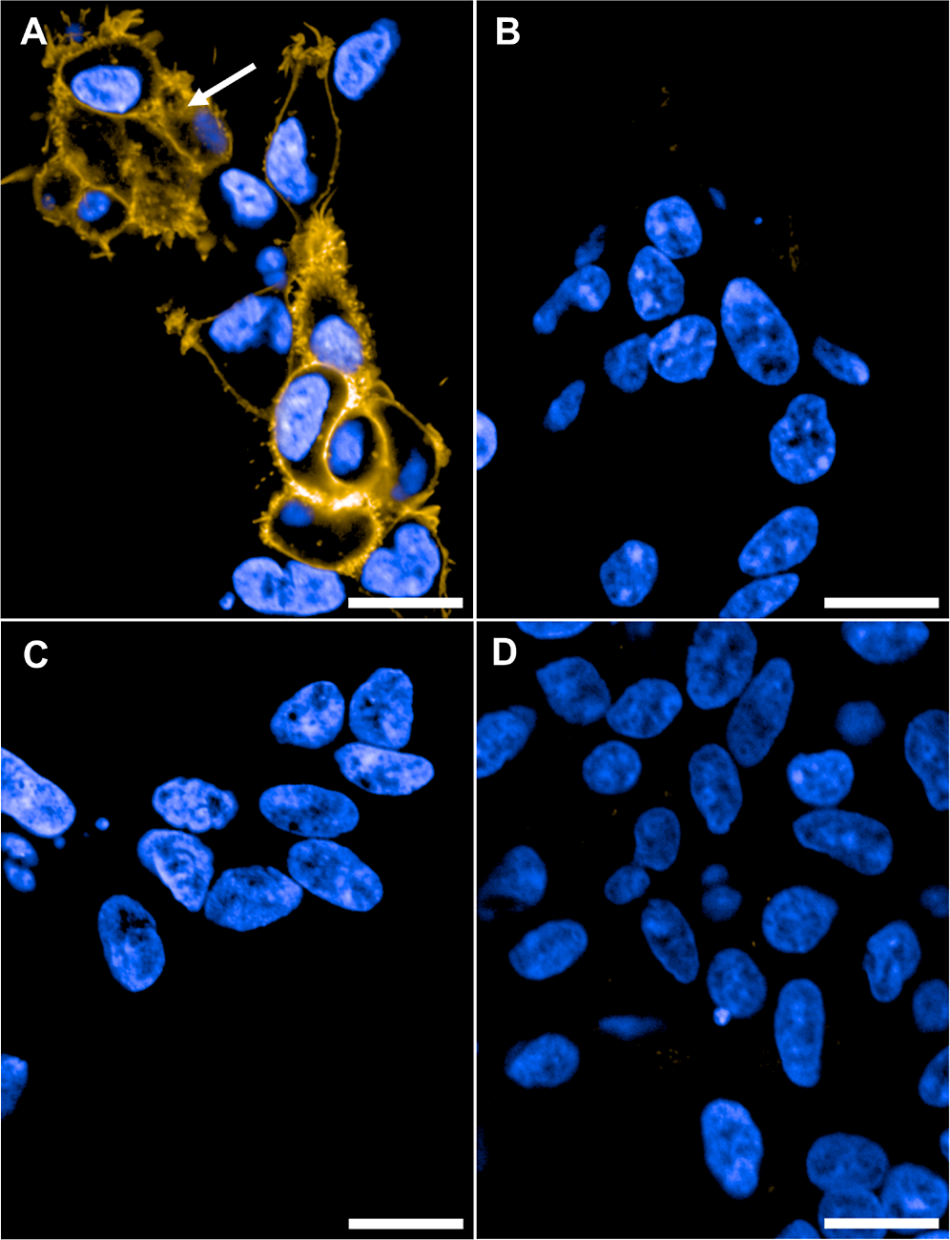
Live cell confocal imaging of HEK293TR cells. (A) Induced CB_1_R-HEK293TR cells incubated with **29** (250 nM, yellow). White arrow: Intracellular staining by **29**. (B) Induced CB_2_R-HEK293TR cells incubated with **29** (250 nM, yellow). (C) Uninduced CB_1_R-HEK293TR cells incubated with **29** (250 nM, yellow). (D) Induced CB_1_R-HEK293TR cells preincubated with competitor **6** (5 μM, 30 min) and then **29** (250 nM, yellow). Images recorded after 10 min at 63× magnification with nucleus counter stain Hoechst 33342 (blue). Scale bars 20 μm images are representative of two to three independent experiments.

Having validated **28** and **29** as selective and useful fluorescent probes for CB_1_R pharmacology investigations, we next examined the potential for visualization of human CB_1_R on live SNAP-CB_1_R-HEK293TR cells by confocal microscopy (Figure 6). Here, we selected the TAMRA bearing probe **29** for further studies, due to the superior photophysical properties (**28**: λ_Abs_: 475 nm, λ_Em_: 550 nm, Φ = 1.3% in PBS and **29**: λ_Abs_: 555 nm, λ_Em_: 585 nm, Φ = 37% in PBS).^83−85^ For rigorous validation of selectivity and specificity, the experiments were performed side-by-side on tetracycline-inducible HEK293TR cells expressing CB_1_R and CB_2_R in comparison with parental HEK293TR cells without CBR expression. Probe **29** was able to selectively stain and visualize CB_1_R on the HEK cells (Figure 6A) within 10 min (see also Video S1). In addition to membrane CB_1_R, we observed staining of intracellular receptor pools of the CB_1_R positive HEK293TR cells (Figure 6A, white arrow, Figures S7 and S8).^7,86^ Since **29** was shown to be an inverse agonist the possibility of ligand-induced internalization of membrane CB_1_R by **29** was excluded.^87^ Accordingly, probe **29** was able to passively permeate the outer cell membrane although exceeding typical drug-like parameters (see Table 4). This confirms the chameleonic behavior predicted by our MD simulation of probe **29**. In contrast, no staining was observed on CB_2_R-HEK293TR or uninduced CB_1_R-HEK293TR (Figure 6B,C). Similarly, the uninduced CB_2_R-HEK293TR and HEK293TR cells without CBRs showed neither any staining nor unspecific background signal (Figures S3 and S4). The rapid staining (see Figures S5 and S6) and excellent CB_1_R-selectivity and specificity emphasize the real-time imaging capabilities of probe **29** and correlate with the selectivity determined in the radioligand binding assay.

## Conclusions

In summary, by using drug-like CB_1_R ligands **5–10,** we systematically explored the compatibility of our modular DEG-based CBR probe design approach. The screening from novel DEG ethyl esters **14–19** over PEG-linked compounds **22–27** to fluorescent probes **28–37** and **40–41** was guided by thorough pharmacological characterization and computational docking studies.

Our study showed that the DEG centerpiece can be used in combination with CB_1_R pharmacophoric units. Unfortunately, while CB_1_R binding and functional activity of the DEG ethyl esters was maintained, selectivity toward CB_1_R was strongly compromised for most structures. Upon the linker exploration, this trend was solidified except for diarylpyrazole series **23a–d**, which turned into selective CB_1_R binders. Optimal linker length and attachment site were investigated by docking studies and confirmed by SAR studies using radioligand displacement assays. Among the tested fluorescent dyes, NBD was well tolerated without affecting the probes selectivity profile. In contrast, TAMRA installation had detrimental effects on the CBR binding affinities, except for **29** and **37**. Most notably, throughout the exploration steps and among the tested structures, pyrrole-based compounds (**14**, **22a–c**, and **28**, **29**) exhibited outstanding selectivity toward CB_1_R and tolerance for any modification. We characterized **28** and **29** as CB_1_R-selective inverse agonist fluorescent probes with applicability in TR-FRET kinetic and equilibrium CB_1_R ligand–receptor binding studies. In live cell confocal fluorescence microscopy, drug-derived **29** showed rapid, highly selective, and specific staining of CB_1_R on HEK293TR cells. The observed membrane permeability of **29** was rationalized by in silico studies suggesting chameleonic effects.

Our novel building block strategy for probe design following reverse-design principles allowed for the fast accomplishment of well-validated, selective, and specific tools for fluorescence-based CB_1_R pharmacology. We believe that our CB_1_R probes will pave the way for a deeper and broader understanding of CB_1_R pharmacology in cannabinoid research.

## Experimental Section

### Radioligand Binding Assay

#### Cell Culture and Membrane Preparation

CHOK1hCB_1__bgal and CHOK1hCB_2__bgal cells (DiscoverRx, Fremont, CA, USA) were cultured in Dulbecco’s modified Eagle’s medium/Nutrient Mixture F-12 Ham supplemented with 10% fetal calf serum, 1 mM glutamine, 50 μg/mL penicillin, 50 μg/mL streptomycin, 300 mg/mL hygromycin, and 800 μg/mL geneticin in a humidified atmosphere at 37 °C and 5% CO_2_. Cells were subcultured twice a week at a ratio of 1:20 on 10 cm plates by trypsinization. For membrane preparation, the cells were subcultured with a ratio of 1:10 and transferred to 15 cm ⌀plates. The cells were collected by scraping in 5 mL phosphate-buffered saline (PBS) and centrifuged at 1000*g* for 5 min. Pellets derived from 30 plates were combined and resuspended in 20 mL cold Tris–HCl, MgCl_2_ buffer (50 mM Tris–HCl (pH 7.4), 5 mM MgCl_2_). The cell suspension was homogenized using an UltraTurrax homogenizer (Heidolph Instruments Schwabach, Germany). Membranes and cytosolic fractions were separated by centrifugation in a Beckman Optima LE-80K ultracentrifuge (Beckman Coulter Inc., Fullerton, CA, USA) at 100,000*g* for 20 min at 4 °C. The supernatant was discarded. The pellet was resuspended in 10 mL cold Tris–HCl, MgCl_2_ buffer, and homogenization and centrifugation steps were repeated. The membranes were resuspended in 10 mL cold Tris–HCl, MgCl_2_ buffer. Aliquots of 100 μL were stored at −80 °C until further use. The protein concentration was determined using the Pierce BCA Protein Assay Kit (ThermoFisher Scientific, Waltham, MA, USA).

#### [^3^H]CP55,940 Displacement Assay

[^3^H]CP55940 displacement assays on 96-well plates were performed in 50 mM Tris–HCl (pH 7.4), 5 mM MgCl_2_, 0.1% BSA assay buffer. Membrane aliquots of either CHOK1hCB_1__bgal or CHOK1CB_2__bgal containing 1 or 2.5 μg membrane protein, respectively, were incubated at 25 °C for 2 h in the presence of ∼1.5 nM [^3^H]CP55,940 (specific activity 106.5 Ci/mmol; Revvity, Waltham, MA, USA). At first, all compounds were tested at a final concentration of 10 μM compound. When radioligand displacement was greater than 50%, full curves were recorded to determine the affinity (p*K*_i_) values of the compounds. To determine the total binding of [^3^H]CP55,940, a control without test compound was included. Nonspecific binding was determined in the presence of 10 μM Rimonabant (CHOK1hCB_1__bgal) or AM630 (CHOK1hCB_2__bgal). The total assay volume was 100 μL. The final concentration of DMSO was 0.25%. The incubation was terminated by rapid vacuum filtration through GF/C 96-well filter plates (Revvity, Waltham, MA), to separate the bound and free radioligand, using a PerkinElmer Filtermate-harvester (Revvity, Waltham, MA, USA). Filters were subsequently washed 20 times with ice-cold assay buffer. The filter-bound radioactivity was determined by scintillation spectrometry using a Microbeta2 2450 microplate counter (Revvity, Waltham, MA, USA), after addition of 25 μL MicroScint-O (Revvity, Waltham, MA, USA) and 3 h incubation.

#### Data Analysis

All experimental data were analyzed using GraphPad Prism 9 (GraphPad Software Inc., San Diego, CA). The data were normalized to % specific radioligand binding, where total binding is 100% and nonspecific binding is 0%. Nonlinear regression for one-site was used to determine the IC_50_ values from the full curve [^3^H]CP55940 displacement assays. The p*K*_i_ values were obtained using the Cheng–Prusoff equation:^88^, where [*L*] is the exact concentration [^3^H]CP55940 determined per experiment and the *K*_D_ is the dissociation constant of [^3^H]CP55940, which is 0.84 and 0.48 nM for CB_1_R and CB_2_R, respectively (data not shown). All data were obtained from at least three separate experiments performed in duplicate.

### HTRF cAMP Assay for CB_1_R

The homogeneous time-resolved fluorescence (HTRF) cAMP assay was conducted following the manufacturer’s protocol for the cAMP-G_s_ Dynamic kit. Briefly, the CHO cell line stably overexpressing CB_1_R was cultured in Ham’s F12 supplemented with 10% FBS, 10 μg/mL blasticidin, and 400 μg/mL zeocin. For the CB_1_R agonist or inverse agonist, dissociated cells were resuspended in Ham’s F12 and dispensed into 384-well low-volume plates at 6000 cells/5 μL per well. The cells were then stimulated with compounds diluted in stimulation buffer (2.5 μL/well) for 15 min at room temperature, followed by the addition of 2.5 μL 5 μM forskolin or 2.5 μL 5 μM forskolin with 100 nM CP55,940 for antagonists. After 15 min, reactions were stopped by the 1× cAMP-*d*_2_ conjugate in lysis buffer (5 μL/well), followed by 1× anti-cAMP cryptate conjugate in lysis buffer (5 μL/well). Following a 1 h incubation at room temperature, the plates were read in a Revvity Envision reader for time-resolved fluorescence resonance energy transfer detection at 620 and 665 nm. The HTRF ratio versus compound concentrations was plotted using Prism 8.1 (GraphPad). HTRF ratio = (signal 665 nm/signal 620 nm) × 10^4^. All HTRF ratio data sets of test compounds were normalized to the *E*_max_ of CP55,940 (100%) or AM281 (−100%) and obtained the means ± standard error of the mean (SEM) of three independent experiments performed in technical replicates.

#### Materials

The cAMP-G_s_ Dynamic kit (PerkinElmer, 62AM4PEC), Ham’s F12 (Gibco, C11330500BT), FBS (Gibco, A5669701), Forskolin (MCE, HY-15371), Zeocin (Gibco, R25001), and Blasticidin (Gibco, A1113903) were used.

### TR-FRET Assay

#### Cell Culture

HEK293TR cells were maintained in a humidified environment at 37 °C and 5% CO_2_ in Dulbecco’s modified Eagle’s medium (DMEM) with 10% fetal bovine serum (FBS) containing blasticidin (5 μg/mL; Invitrogen) and (Zeocin; 20 μg/mL; Invitrogen). For inducible expression, a SNAP-tagged human CB_1_R cDNA (in TR-FRET experiments, a truncated CB_1_R variant, CB_1_R_91-472_ was used to facilitate the FRET, and named based on the residues remaining after truncation) in pcDNA4/TO was introduced through transfection, using PEI into HEK293TR cells (Invitrogen, which express Tet repressor protein to allow inducible expression). A mixed population stable line was selected by resistance to blasticidin (TR vector, 5 μg/mL) and Zeocin; (receptor plasmid, 20 μg/mL). For receptor-inducible expression, cells were seeded into T175 flasks, grown to 70% confluence, and DMEM containing 1 μg/mL tetracycline added. 24 h later, cells were labeled with SNAP-Lumi4-Tb (CisBio) and membranes prepared as described in detail below.

#### Terbium Labeling of SNAP-Tagged CB_1_R HEK293-TR Cells

Cell culture medium was removed from the T175 flasks containing confluent adherent CB_1_R HEK293-TR cells. Cells were washed 1× in PBS (GIBCO Carlsbad, CA), followed by 1× Tag-lite labeling medium (LABMED, CisBio) to remove the excess cell culture media, and then 10 mL of LABMED containing 100 nM of SNAP-Lumi4-Tb was added to the flask and incubated for 1 h at 37 °C under 5% CO_2_. Cells were washed 1× in PBS (GIBCO Carlsbad, CA) to remove the excess of SNAP-Lumi4-Tb, then detached using 5 mL of GIBCO enzyme-free Hank’s-based cell dissociation buffer (GIBCO, Carlsbad, CA), and collected in a vial containing 5 mL of DMEM (Sigma-Aldrich) supplemented with 10% fetal calf serum. Cells were pelleted by centrifugation (5 min at 350*g*), and the pellets were frozen to −80 °C.

#### Membrane Preparation

All steps were conducted at 4 °C to avoid tissue degradation. Cell pellets were thawed and resuspended using ice-cold buffer containing 10 mM HEPES and 10 mM EDTA, pH 7.4. The suspension was homogenized using an electrical homogenizer Ultra-Turrax (Ika-Werk GmbH & Co. KG, Staufen, Germany) and subsequently centrifuged at 1200*g* for 5 min. The pellet obtained then, containing cell nucleus and other heavy organelles, was discarded, and supernatant was centrifuged for 30 min at 48,000*g* at 4 °C (Beckman Avanti J-251 Ultracentrifuge; Beckman Coulter, Fullerton, CA). The supernatant was discarded, and the pellet was resuspended using the same buffer (10 mM HEPES and 10 mM EDTA, pH 7.4) and centrifuged for a second time for 30 min as described above. Finally, the supernatant was discarded, and the pellet resuspended using ice-cold 10 mM HEPES and 0.1 mM EDTA, pH 7.4. Protein concentration determination was carried out using the bicinchoninic acid assay kit (Sigma-Aldrich) and using BSA as a standard. The final membrane suspension was aliquoted and maintained at −80 °C until required for the binding assays.

#### Fluorescent Ligand-Binding Assays

All fluorescent ligand binding experiments were conducted in white 384-well Optiplate plates, in assay binding buffer, either Hanks Balanced Salt Solution (HBSS), 5 mM HEPES, 0.5% BSA, 0.02% pluronic F-127 pH 7.4, and 100 μM GppNHp. GppNHp was included to remove the G protein-coupled population of receptors that can result in two distinct populations of binding sites in membrane preparations since the Motulsky–Mahan model is only appropriate for ligands competing at a single site. In all cases, nonspecific binding was determined by the presence of 10 μM Rimonabant.

#### Determination of Fluorescent Ligand Binding Kinetics and Equilibrium Affinity

To accurately determine the association rate (*k*_on_) and dissociation rate (*k*_off_) values, the observed rate of association (*k*_obs_) was calculated using at least six different concentrations of fluorescent ligand. The appropriate concentration of fluorescent ligand binding was incubated with human CB_1_R HEK293-TR cell membranes (0.5 μg per well) in assay binding buffer (final assay volume, 40 μL). The degree of fluorescent ligand bound to the receptor was assessed at multiple time points by HTRF detection to allow for the construction of association kinetic curves. The resulting data were globally fitted to the association kinetic model (eq 1, see Signal Detection and Data Analysis section below) to derive a single best-fit estimate for *k*_on_ and *k*_off_ as described under data analysis. Saturation analysis was performed at equilibrium, by simultaneously fitting total and nonspecific (NSB) binding data (eq 2, see Signal Detection and Data Analysis section below) allowing for the determination of fluorescent ligand binding affinity.

#### Competition Binding

To determine the affinity of CB_1_R-selective ligands, we used a simple competition kinetic binding assay. This approach involves the simultaneous addition of both fluorescent ligand and competitor to the CB_1_R preparation. Compounds were added simultaneously with increasing concentrations of the unlabeled compound to CB_1_R cell membranes (0.5 μg per well) in 40 μL of assay buffer in a 384-well Optiplate incubated at room temperature with orbital mixing. The degree of fluorescent ligand bound to the receptor was assessed at equilibrium by HTRF detection. Nonspecific binding was determined as the amount of HTRF signal detected in the presence of Rimonabant (10 μM) and was subtracted from total binding, to calculate specific binding for construction of IC_50_ curves.

#### Signal Detection and Data Analysis

Signal detection was performed on a PHERAstar FSX (BMG Labtech, Offenburg, Germany). The terbium donor was always excited with four laser flashes at a wavelength of 337 nm. TR-FRET signals were collected at 590 (acceptor) and 620 nm (donor) when using the orange acceptor fluorescent ligand or at 520 (acceptor) and 620 nm (donor) when using the green acceptor fluorescent ligand. HTRF ratios were obtained by dividing the acceptor signal by the donor signal and multiplying this value by 10,000. All experiments were analyzed by nonregression using Prism 8.0 (GraphPad Software, San Diego, USA). Fluorescent ligand association data were fitted as follows to a global fitting model using GraphPad Prism 8.0 to simultaneously calculate *k*_on_ and *k*_off_ using the following equation
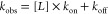
1
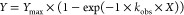
where *k*_obs_ equals the observed rate of ligand association and *k*_on_ and *k*_off_ are the association and dissociation-rate constants, respectively, of the fluorescent ligand. In this globally fitted model of tracer binding, tracer concentrations [*L*] are fixed, *k*_on_ and *k*_off_ are shared parameters, while *k*_obs_ is allowed to vary. Here, *Y* is the level of receptor-bound tracer, *Y*_max_ is the level of tracer binding at equilibrium, *X* is in units of time (e.g., min), and *k*_obs_ is the rate in which equilibrium is approached (e.g., min^–1^). Saturation binding data were analyzed by nonlinear regression according to a one-site equation by globally fitting total and NSB. Individual estimates for the fluorescent ligand dissociation constant (*K*_D_) were calculated using the following equations where *L* is the fluorescent ligand concentration
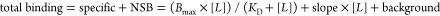
2
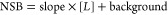


Fitting the total and NSB data sets globally (simultaneously), sharing the value of slope, provides one best-fit value for both the *K*_D_ and the *B*_max_. Competition displacement binding data were fitted to sigmoidal (variable slope) curves using a “four-parameter logistic equation”
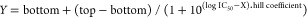
3

IC_50_ values obtained from the inhibition curves were converted to *K*_i_ values using the method of Cheng and Prusoff.^88^
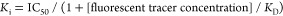
4

### Imaging Experiments

#### Cell Culture

Human CB_1_R HEK293TR cells (same transfected cell line as for TR-FRET binding assay was used, see above) were maintained in a humidified environment at 37 °C and 5% CO_2_ in DMEM with 10% FBS containing blasticidin (5 μg/mL; Invitrogen) and (Zeocin; 20 μg/mL; Invitrogen).

#### Methodology

Cells were plated onto a 384-well microplate (PhenoPlate, Revvity), at a density of 3500 cells/well (40 μL) with 1 μg/mL tetracycline for receptor-inducible expression and incubated for 48 h. The cell nuclei were stained using 0.9 μM Hoechst 33342 for 1 h incubation. After replacement of medium to serum-free conditions without Phenol red (20 μL), fluorescent probes were added (10 μL) and tested at 250 nM concentration. In case of blocking experiments, nuclei-stained cells were incubated with inhibitors (5 μM) for 30 min under serum-free conditions before probe administration. Confocal live cell imaging was performed using the Opera Phenix High Content Screening System (Revvity) at 22 °C. The probe fluorescence was monitored by kinetic measurements of 10 min with a break for probe administration. The fluorescence of one image per sample was captured using a water immersion objective (63×, NA 1.15, field of view 0.21 × 0.21 mm) at each time point. Probe detection was realized using the appropriate laser for excitation and filter for fluorescence emission. Image acquisition parameters, including laser power, offset, and gain settings, were kept constant.

### Computational Docking

The previously reported X-ray diffraction structure for CB_1_R complexed with the CB_1_R antagonist, AM6538 (PDB: 5TGZ),^56^ was used as a template to dock CB_1_R compounds. Docking experiments were performed interactively using MOE software (Chemical Computing Group) with default settings [Molecular Operating Environment (MOE), 2022.02; Chemical Computing Group ULC, 1010 Sherbrooke St. West, Suite #910, Montreal, QC, Canada, H3A 2R7, 2022]. The most reasonable docking pose with respect to molecular interactions and internal conformational strain was energy-minimized within the binding pocket. Adjacent amino acid side chains were energy-minimized without restraints. The resulting docking poses were checked for consistency with the available structure–activity relationship (SAR) information. Visualization was performed using Maestro Schrödinger.

### Photophysical Characterization

#### Absorbance/Emission Determination

50 μL of 10 μM solution of compound **28–37** and **40–41** in PBS (pH = 7.4) in the presence of 0.1% (v/v) DMSO was placed in a Corning 384-well Polystyrene microplates and the UV/vis absorbance spectra were first recorded in the wavelength range of 300–800 nm (scan step 5 nm) to determine the wavelength with the maximal absorbance signal, which was later used for excitation of the corresponding compound and fluorescent emission signal measurements. All measurements were performed at room temperature using a Tecan Safire II UV–vis fluorescence and absorbance plate reader.

#### Quantum Yield Determination

The absolute quantum yield was determined using a HAMAMATSU PHOTONICS K.K Absolute PL Quantum Yield Spectrometer with xenon lamp bulb L11562. For this purpose, 3 mL of 100 nM solution of compound in PBS (pH = 7.4) in the presence of 0.1% (v/v) DMSO were placed into a quartz cuvette with a rod (Size: 12.5 × 12.5 × 140 mm). After excitation, the quantum yield was recorded with the supplier’s software version 4.6.0 CD-ROM and reported as percentage.

### Synthesis Procedures and Analytical Data for the Compounds

Reactions with air or moisture-sensitive substances were carried out under an inert atmosphere of nitrogen with the help of the Schlenk technique, if not otherwise indicated. All chemicals were purchased from commercial suppliers and used as received unless otherwise specified. 6-(4-Chlorophenyl)-5-(2-methoxyethoxy)pyrazine-2-carboxylic acid (**46**) (CAS RN 960248-07-1), 6-(cyclopropylmethoxy)-5-(4-fluorophenyl)nicotinic acid (**45**) (CAS RN 912454-39-8) and (*R*)-5-(2,5-bis(trifluoromethyl)phenyl)-2-methyl-1-((tetrahydrofuran-2-yl)methyl)-1*H*-pyrrole-3-carboxylic acid (**42**) (WO2005108393A1) were obtained from Roche, Basel, Switzerland. 5-(4-Chlorophenyl)-1-(2,4-dichlorophenyl)-4-methyl-1*H*-pyrazole-3-carboxylic acid (**43**) was commercially available from Ambeed, Arlington Heights, IL, USA. 8-Chloro-1-(2,4-dichlorophenyl)-1,4,5,6-tetrahydrobenzo[6,7]cyclohepta[1,2-*c*]pyrazole-3-carboxylic acid (**44**) and 1-(3-fluorobenzyl)-1*H*-indazole-3-carboxylic acid (**47**) were synthesized according to the synthetic routes describes below. Compound names are derived from Chemdraw and are not necessarily identical to the IUPAC nomenclature. For thin layer chromatography aluminum backed silica gel plates were used (silica gel 60 F 254 from E. Merck), visualizing with UV light (λ = 254 nm). Microwave heating of reactions was carried out on a Biotage Initiator + apparatus. Chromatographic separations were carried out using Biotage Isolera One apparatus or Combiflash NextGen 300+ apparatus with RediSepRf columns from Teledyne Isco. High-performance liquid chromatography (HPLC) separations were carried out using a Gilson PLC 2050 system, a Gilson PLC 2250 or a Shimadzu system with the following components: CBM20A, LC20AP, SPD, 20A, and FC200Al. The Gilson systems were used with an automated gradient optimizer. As the stationary phase, a Macherey-Nagel VP 250/21 Nucleodur 100-7 C18Ec column or a Macherey-Nagel VP 250/10 Nucleodur 100-5 C18Ec column was used. As the mobile phase, ACN/water with 0.1% TFA as an acidic modifier or ACN/water was used. The analytical data was obtained with the help of the following equipment: ^1^H and ^13^C NMR spectra were recorded at either Bruker AV 300 (295 K, 300 MHz, 75 MHz), Bruker AV 600 (300 K, 600 MHz, 151 MHz), or Bruker AV 750cryo (300 K, 750 MHz, 189 MHz) spectrometers in CDCl_3_, MeOD, ACN-*d*_3_ or DMSO-*d*_6_ as solvents. Spin multiplicities were described as singlet (s), doublet (d), triplet (t), quartet (q), multiplet (m) doublet of doublet (dd), doublet of triplet (dt), doublet of quartet (dq), and broad-singlet (br s). Coupling constants (^*n*^*J*, whereby n equals the number of bonds between the coupled nuclei) were recorded in Hz. All ^13^C NMR-spectra were recorded with ^1^H-broad-band decoupling. All chemical shifts are reported as found in ppm (δ) relative to tetramethylsilane (δ = 0.00 ppm) and were calibrated with respect to their deuterated solvents.^89^ NMR data were analyzed with MNova. Analytical HPLC-MS and purity analyses were performed with Agilent 1260 series HPLC system employing a DAD detector (at 300, 254, and 220 nm) equipped with Agilent Technologies 6120 Quadrupole LC/MS in electrospray positive and negative ionization modes (ESI-MS). A Thermo Accuore RP-MS (30 × 2.1 mm, 2.6 μm) column was used with a flow rate 0.8 mL/min in combination with the following separation conditions: 0.1% formic acid in water (solvent A); 0.1% formic acid in ACN (solvent B); system (1) 5% B for 0.5 min, from 5 to 95% B in 6.5 min, 95% B for 1 min (stop point at 8 min); system (2) 5% B for 0.2 min, from 5 to 95% B in 0.9 min, 95% B for 1.4 min (stop point at 2.5 min). Data analysis was performed with ChemStation software. All compounds are >95% pure by HPLC. High-resolution mass spectrometry (HRMS) analyses were carried out on Agilent Technologies 6530 Accurate Mass Q-ToF LC/MS linked to Agilent Technologies HPLC 1260 Infinity II and HRMS results are reported in *m*/*z*.

#### Ethyl 2-Amino-2-ethylbutanoate Hydrochloride (**13**)

Thionyl chloride (3.50 mL, 47.9 mmol) was added dropwise to a solution of (**11**) (3.80 g, 36.9 mmol) in EtOH (40 mL), over a period of 5 min at 0 °C. The reaction mixture was stirred at 0 °C for 1 additional hour. Afterward, the resulting solution was refluxed for 4 h (80 °C). The reaction mixture was cooled to rt and concentrated under reduced pressure to yield a colorless oil (quant.), using crude ethyl 2-aminobutanoate for the next step. The crude (4.80 g, 36.9 mmol) and dried magnesium sulfate (4.40 g, 36.9 mmol) were stirred in dry dichloromethane (30 mL) at ambient temperature for 20 min. Afterward, benzaldehyde (3.80 mL, 36.9 mmol) and dry triethylamine (9.50 mL, 68.2 mmol) were added sequentially and dropwise. The resulting mixture was stirred for 30 h at the same temperature and then filtered, and the solvent was evaporated. The residue was dissolved in ether (8 mL) and water (8 mL) and the separated aqueous layer extracted with ether (2 × 8 mL). The combined ether solutions were washed with brine, then dried, filtered, and concentrated under reduced pressure to leave the imine as a clear oil. The crude intermediate product (ethyl 2-(benzylideneamino) butyrate) (**12**) was used for the further step. Potassium bis(trimethylsilyl)amide (5.50 g, 27.5 mmol) in THF (30 mL) was added dropwise to a solution of (**12**) (4.00 g; 18.3 mmol) in THF (10 mL) cooled to −70 °C. After 1 h, iodoethane (1.90 mL, 23.7 mmol) was added at the same temperature. The cooling bath was removed, and the mixture was stirred at room temperature for an additional 20 h. Afterward, the reaction was concentrated under reduced pressure, to remove most of the tetrahydrofuran. The residue was then partitioned between dichloromethane and water. The organic layer was separated, and the aqueous phase was extracted with dichloromethane (4×). The combined organic extracts were washed with brine, dried (MgSO_4_), filtered, and concentrated to yield intermediate ethyl-2-(benzylideneamino)-2-ethylbutanoate (quant.). To a solution of ethyl-2-(benzylideneamino)-2-ethylbutanoate (4.50 mg, 18.3 mmol) in diethyl ether (12 mL) under inert atm., HCl 5 M (13 mL) was added dropwise at 0 °C. After the addition, the reaction mixture was allowed to warm to room temperature and stirred for an additional 15 h. The ether layer was then separated, and the water phase was extracted with dichloromethane (2×). The dichloromethane extracts were extracted with HCl 2 M (2×). The aqueous layers were combined and concentrated to give a yellow solid (3.02 g, 84%). ^1^H NMR (300 MHz, CDCl_3_) δ (ppm): 9.02–8.65 (m, 3H), 4.27 (q, ^3^*J* = 7.0 Hz, 2H), 2.18–1.91 (m, 2H), 1.30 (t, ^3^*J* = 7.1 Hz, 3H), 1.09 (t, ^3^*J* = 7.4 Hz, 6H). LC–MS (ESI+) *m*/*z*: [M + H]^+^ calcd for C_8_H_17_NO_2_, 160.1332; found, 160.3.

#### Ethyl (*R*)-2-(5-(2,5-Bis(trifluoromethyl)phenyl)-2-methyl-1-((tetrahydrofuran-2-yl)methyl)-1*H*-pyrrole-3-carboxamido)-2-ethylbutanoate (**14**)

To a solution of (**42**) (18.4 mg, 85.6 μmol, 1.0 equiv) and HATU (32.5 mg, 85.6 μmol, 1.0 equiv) in ACN/DCM (1 mL, v/v 1:1) was added DIPEA (36.3 μL, 27.6 mg, 214 μmol, 2.5 equiv). The solution was stirred for 20 min before (**13**) (18.4 mg, 94.1 μmol, 1.1 equiv) was added. After 22 h, the mixture was concentrated under reduced pressure. The residue was taken up in ACN/H_2_O (v/v, 1/1), filtered, and purified by reversed-phase preparative HPLC (30–95% ACN + 0.1% TFA/H_2_O + 0.1% TFA). The title compound was obtained as a white powder (14.2 mg, 25.2 μmol, 28%) after lyophilization. ^1^H NMR (300 MHz, CDCl_3_) δ (ppm): 8.00–7.60 (m, 3H), 6.74 (s, 1H), 6.35 (s, 1H), 4.24 (q, ^3^*J* = 7.1 Hz, 2H), 4.10–3.33 (m, 5H), 2.71–2.52 (m, 5H), 1.96–1.59 (m, 6H), 1.29 (t, ^3^*J* = 7.1 Hz, 3H), 0.79 (t, ^3^*J* = 7.4 Hz, 6H). HR-MS (ESI+) *m*/*z*: [M + H]^+^ calcd for C_27_H_32_F_6_N_2_O_4_, 563.2339; found, 563.2361.

#### Ethyl 2-(5-(4-Chlorophenyl)-1-(2,4-dichlorophenyl)-4-methyl-1*H*-pyrazole-3-carboxamido)-2-ethylbutanoate (**15**)

To a solution of (**43**) (42.0 mg, 111 μmol, 1.0 equiv) and HATU (42.0 mg, 111 μmol, 1.0 equiv) in ACN (2 mL) was added DIPEA (57.0 μL, 43.0 mg, 333 μmol, 3.0 equiv). The solution was stirred for 20 min before (**13**) (26.0 mg, 133 μmol, 1.2 equiv) was added. The reaction was stirred for 16 h. To prepare the mixture for purification, H_2_O was added (2 mL), and the mixture was filtered and then purified by reversed-phase preparative HPLC (40–95% ACN + 0.1% TFA/H_2_O + 0.1% TFA). The title compound was obtained as a white powder (22.9 mg, 43.7 μmol, 40%) after lyophilization. ^1^H NMR (600 MHz, MeOD) δ (ppm): 7.57 (d, ^4^*J* = 2.2 Hz, 1H), 7.53 (d, ^3^*J* = 8.5 Hz, 1H), 7.45 (dd, ^3^*J* = 8.5, ^4^*J* = 2.1 Hz, 1H), 7.36 (d, ^3^*J* = 8.5 Hz, 2H), 7.20 (d, ^3^*J* = 8.5 Hz, 2H), 4.26 (q, ^3^*J* = 7.1 Hz, 2H), 2.40 (dq, ^2^*J* = 14.7, ^3^*J* = 7.5 Hz, 2H), 2.29 (s, 3H), 1.96 (dq, ^2^*J* = 14.7, ^3^*J* = 7.4 Hz, 2H), 1.29 (t, ^3^*J* = 7.1 Hz, 3H), 0.83 (t, ^3^*J* = 7.5 Hz, 6H). ^13^C NMR (151 MHz, MeOD) δ (ppm): 174.88, 163.48, 145.99, 144.84, 137.35, 137.31, 136.22, 134.16, 132.52, 132.49, 131.10, 129.88, 129.23, 128.63, 118.37, 66.59, 62.84, 28.75, 14.53, 9.55, 8.60. HR-MS (ESI+) *m*/*z*: [M + H]^+^ calcd for C_25_H_26_Cl_3_N_3_O_3_, 522.1113; found, 522.1125.

#### Ethyl 2-(3-Chloro-9-hydroxy-6,7-dihydro-5*H*-benzo[7]annulen-8-yl)-2-oxoacetate (**48**)

Na (460 mg, 20.0 mmol) was dissolved in absolute EtOH (13 mL) under a N_2_ atmosphere. After complete dissolution of the metal, diethyl oxalate (599 mg, 4.10 mmol, 2.5 equiv) was added via a syringe before 2-chloro-6,7,8,9-tetrahydro-5*H*-benzo[7]annulen-5-one (317 mg, 1.60 mmol, 1.0 equiv) in absolute EtOH (20 mL) was added dropwise over a period of 30 min via syringe. The reaction was stirred for 16 h and acidified with HCl (2 M), while cooling with an ice bath. The mixture was extracted with CHCl_3_ (4 × 20 mL). The combined organic layers were dried over anhydrous Na_2_SO_4_, filtered, and concentrated under reduced pressure. The residue was purified by automated silica gel chromatography (SiO_2_, 0 → 100 EtOAc). Fractions containing the product were combined and concentrated under reduced pressure. The title compound was obtained as a yellow oil (336.7 mg, 1.10 mmol, 69%). ^1^H NMR (300 MHz, CDCl_3_) δ (ppm): 7.58 (d, ^3^*J* = 8.2 Hz, 1H), 7.34 (dd, ^3^*J* = 8.3, ^4^*J* = 2.1 Hz, 1H), 7.24 (d, ^4^*J* = 2.1 Hz, 1H), 4.44–4.27 (m, 3H), 2.71 (t, ^3^*J* = 7.0 Hz, 2H), 2.31 (t, ^3^*J* = 6.7 Hz, 2H), 2.07 (quint, ^3^*J* = 6.8 Hz, 2H), 1.40 (t, ^3^*J* = 7.1 Hz, 5H). LC–MS (ESI+) *m*/*z*: [M + H]^+^ calcd for C_15_H_15_ClO_4_, 295.0732; found, 295.0. Analytical data correspond to previous reports.^90^

#### Ethyl 8-Chloro-1-(2,4-dichlorophenyl)-1,4,5,6-tetrahydrobenzo[6,7]cyclohepta[1,2-*c*]pyrazole-3-carboxylate (**49**)

To a solution of (**48**) (337 mg, 1.10 mmol, 1.0 equiv) in EtOH (15 mL) in a microwave vial was added 2,4-dichlorophenylhydrazine hydrochloride (272 mg, 1.30 mmol, 1.3 equiv). The vial was capped and submitted to a microwave reactor (80 °C, 12 h). The solvent was removed under reduced pressure. The crude residue was purified by automated silica gel chromatography (SiO_2_, 0 → 100 EtOAc). The title compound was obtained as an orange foam (440 mg, 1.0 mmol, 91%). ^1^H NMR (300 MHz, CDCl_3_) δ (ppm): 7.53 (d, ^3^*J* = 8.8 Hz, 1H), 7.42–7.36 (m, 2H), 7.31 (d, ^4^*J* = 2.2 Hz, 1H), 7.02 (dd, ^3^*J* = 8.3, ^4^*J* = 2.2 Hz, 1H), 6.60 (d, ^3^*J* = 8.3 Hz, 1H), 4.45 (q, ^3^*J* = 7.1 Hz, 2H), 3.37–3.07 (m, 2H), 2.66 (t, ^3^*J* = 6.6 Hz, 2H), 2.41–2.12 (m, 2H), 1.43 (t, ^3^*J* = 7.1 Hz, 3H). LC–MS (ESI+) *m*/*z*: [M + H]^+^ calcd for C_21_H_17_Cl_3_N_2_O_2_, 435.0428; found, 435.0. Analytical data corresponds with previous reports.^90^

#### 8-Chloro-1-(2,4-dichlorophenyl)-1,4,5,6-tetrahydrobenzo[6,7]cyclohepta[1,2-*c*]pyrazole-3-carboxylic Acid (**44**)

A solution of (**49**) (26.2 mg, 60.2 μmol, 1.0 equiv) and LiOH (13.8 mg, 329 μmol, 5.5 equiv) in methanol (15 mL) was stirred at room temperature for 4.5 h. The solvent was removed under reduced pressure. The residue was taken up in H_2_O (25 mL) and acidified with HCl (1 M). The resulting suspension was extracted with EtOAc (3 × 15 mL). The combined organic layers were dried over anhydrous Na_2_SO_4_, filtered, and concentrated under reduced pressure. The title compound was obtained as an orange solid (19.0 mg, 46.6 μmol, 78%). ^1^H NMR (300 MHz, CDCl_3_) δ (ppm): 7.51 (d, ^3^*J* = 8.3 Hz, 1H), 7.46–7.39 (m, 2H), 7.32 (d, ^4^*J* = 2.2 Hz, 1H), 7.03 (dd, ^3^*J* = 8.3, ^4^*J* = 2.2 Hz, 1H), 6.61 (d, ^3^*J* = 8.3 Hz, 1H), 3.42–2.76 (m, 2H), 2.68 (t, ^3^*J* = 6.5 Hz, 2H), 2.36–2.17 (m, 2H). LC–MS (ESI+) *m*/*z*: [M + Na]^+^ calcd for C_19_H_13_Cl_3_N_2_O_2_, 407.0115; found, 407.0. Analytical data corresponds with previous reports.^90^

#### Ethyl 2-(8-Chloro-1-(2,4-dichlorophenyl)-1,4,5,6-tetrahydrobenzo[6,7]cyclohepta[1,2-*c*]pyrazole-3-carboxamido)-2-ethylbutanoate (**16**)

To a solution of (**44**) (18.0 mg, 44.2 μmol, 1.0 equiv) and HATU (17.0 mg, 44.2 μmol, 1.0 equiv) in DMF (2 mL) was added DIPEA (30.0 μL, 23.0 mg, 177 μmol, 4.0 equiv). The solution was stirred for 20 min before (**13**) (8.00 mg, 53.0 μmol, 1.2 equiv) was added. The reaction was stirred for 5 h. Upon incomplete conversion, another portion of (**13**) (9.30 mg, 58.0 μmol, 1.3 equiv) was added together with EDCI (8.50 mg, 44.2 μmol, 1.0 equiv) and DIPEA (30.0 μL, 23.0 mg, 177 μmol, 4.0 equiv). After another 15 h, the solvent was removed under reduced pressure and the residue was taken up in ACN/H_2_O (v/v, 1/1), filtered, and purified by reversed-phase preparative HPLC (50–95% ACN + 0.1% TFA/H_2_O + 0.1% TFA). The title compound was obtained as a white powder (8.92 mg, 16.3 μmol, 37%) after lyophilization. ^1^H NMR (600 MHz, MeOD) δ 7.69 (d, ^3^*J* = 8.5 Hz, 1H), 7.61 (d, ^4^*J* = 2.3 Hz, 1H), 7.56 (dd, ^3^*J* = 8.6, ^4^*J* = 2.3 Hz, 1H), 7.39 (d, ^4^*J* = 2.2 Hz, 1H), 7.08 (dd, ^3^*J* = 8.4, ^4^*J* = 2.1 Hz, 1H), 6.71 (d, ^3^*J* = 8.3 Hz, 1H), 4.26 (q, ^3^*J* = 7.1 Hz, 2H), 2.70 (t, ^3^*J* = 6.6 Hz, 2H), 2.40 (dq, ^2^*J* = 14.9, ^3^*J* = 7.5 Hz, 2H), 2.25 (t, ^3^*J* = 7.0 Hz, 2H), 1.96 (dq, ^2^*J* = 14.7, ^3^*J* = 7.4 Hz, 2H), 1.29 (t, ^3^*J* = 7.1 Hz, 3H), 0.83 (t, ^3^*J* = 7.4 Hz, 6H). ^13^C NMR (151 MHz, MeOD) δ (ppm): 174.87, 163.47, 145.22, 145.04, 144.02, 137.35, 137.29, 135.54, 133.62, 132.35, 131.31, 130.92, 129.67, 129.59, 129.09, 127.31, 122.98, 66.59, 62.87, 33.19, 32.74, 28.70, 21.13, 14.55, 8.63. HR-MS (ESI+) *m*/*z*: [M + Na]^+^ calcd for C_27_H_28_Cl_3_N_3_O_3_, 570.1088; found, 570.1090.

#### Ethyl 2-(6-(Cyclopropylmethoxy)-5-(4-fluorophenyl)nicotinamido)-2-ethylbutanoate (**17**)

To a solution of (**45**) (35.3 mg, 123 μmol, 1.0 equiv) and HATU (48.3 mg, 123 μmol, 1.0 equiv) in ACN/DCM (1 mL, v/v, 1:1) was added DIPEA (31.0 μL, 23.6 mg, 184 μmol, 1.5 equiv). The solution was stirred for 20 min before (**13**) (21.6 mg, 135 μmol, 1.1 equiv) was added. After 5 h, the mixture was concentrated under reduced pressure. The residue was taken up in ACN/H_2_O (v/v, 1/1), filtered, and purified by reversed-phase preparative HPLC (20–95% ACN/H_2_O). The title compound was obtained as a white powder (5.80 mg, 13.5 μmol, 11%) after lyophilization. ^1^H NMR (600 MHz, CDCl_3_) δ (ppm): 8.58 (d, ^4^*J* = 2.4 Hz, 1H), 8.02 (d, ^4^*J* = 2.4 Hz, 1H), 7.63–7.58 (m, 2H), 7.16 (s, 1H), 7.14–7.09 (m, 2H), 4.33–4.25 (m, 4H), 2.64 (dq, ^2^*J* = 14.9, ^3^*J* = 7.5 Hz, 2H), 1.89 (dq, ^2^*J* = 14.6, ^3^*J* = 7.3 Hz, 2H), 1.33 (t, ^3^*J* = 7.1 Hz, 3H), 1.29 (s, 1H), 0.79 (t, ^3^*J* = 7.4 Hz, 6H), 0.61–0.55 (m, 2H), 0.38–0.32 (m, 2H). ^13^C NMR (151 MHz, CDCl_3_) δ (ppm): 174.6, 164.2, 162.6 (d, ^1^*J*_C–F_ = 247.6 Hz), 162.6, 144.9, 137.4, 132.0 (d, ^4^*J*_C–F_ = 3.0 Hz), 131.1 (d, ^3^*J*_C–F_ = 8.1 Hz), 124.6, 123.5, 115.36 (d, ^2^*J*_C–F_ = 21.5 Hz), 71.6, 66.8, 62.2, 28.4, 14.4, 10.2, 8.7, 3.4. HR-MS (ESI+) *m*/*z*: [M + H]^+^ calcd for C_24_H_29_FN_2_O_4_, 429.2184; found, 429.2194.

#### Ethyl 2-(6-(4-Chlorophenyl)-5-(2-methoxyethoxy)pyrazine-2-carboxamido)-2-ethylbutanoate (**18**)

To a solution of (**46**) (28.7 mg, 93.0 μmol, 1.0 equiv) and HATU (35.3 mg, 93.0 μmol, 1.0 equiv) in ACN/DCM (1 mL, v/v, 1:1) was added DIPEA (39.4 μL, 30.0 mg, 232 μmol, 2.5 equiv). The solution was stirred for 20 min before (**13**) (20.0 mg, 102 μmol, 1.1 equiv) was added. After completion of the reaction, DCM (5 mL) was added and the organic layer was washed with NaHCO_3_ (2 × 10 mL, 0.5 M). The combined aqueous layers were re-extracted with DCM (5 mL) before the combined organic layers were dried over Na_2_SO_4_, filtered, and concentrated under reduced pressure. The residue was taken up in ACN/H_2_O (v/v, 1/1), filtered, and purified by reversed-phase preparative HPLC (20–95% ACN + 0.1% TFA/H_2_O + 0.1% TFA). The title compound was obtained as a white powder (16.6 mg, 36.9 μmol, 41%) after lyophilization. ^1^H NMR (600 MHz, CDCl_3_) δ (ppm): 8.82 (s, 1H), 8.77 (CON*H*, s, 1H), 8.19 (d, ^3^*J* = 8.6 Hz, 2H), 7.48 (d, ^3^*J* = 8.6 Hz, 2H), 4.68–4.64 (m, 2H), 4.30 (q, ^3^*J* = 7.1 Hz, 2H), 3.83–3.78 (m, 2H), 3.43 (s, 3H), 2.62 (dq, ^2^*J* = 14.9, ^3^*J* = 7.5 Hz, 2H), 1.93 (dq, ^2^*J* = 14.6, ^3^*J* = 7.4 Hz, 2H), 1.34 (t, ^3^*J* = 7.1 Hz, 3H), 0.80 (t, ^3^*J* = 7.4 Hz, 6H). ^13^C NMR (151 MHz, CDCl_3_) δ (ppm): 174.05, 162.30, 159.19, 139.62, 139.59, 137.81, 136.10, 133.55, 130.86, 128.81, 70.71, 66.65, 66.55, 62.02, 59.32, 28.62, 14.53, 8.86. HR-MS (ESI+) *m*/*z*: [M + H]^+^ calcd for C_22_H_28_ClN_3_O_5_, 450.1790; found, 450.1810.

#### Methyl 1-(3-Fluorobenzyl)-1*H*-indazole-3-carboxylate (**50**)

To a solution of methyl 1*H*-indazole-3-carboxylate (214 mg, 1.20 mmol, 1.0 equiv) in THF (10 mL) was added a solution of KO*t*Bu (12% in THF, 1.45 mL, 1.45 mmol, 1.2 equiv) at 0 °C under N_2_-atm. The solution was stirred for 30 min before 1-(bromomethyl)-3-fluorobenzene was added dropwise in THF (5 mL) at 0 °C. The solvent was removed, and the material was purified on automated silica gel chromatography (SiO_2_, 0 → 20% EtOAc in CyHex). The title compound was obtained as a clear oil (270 mg, 1.00 mmol, 78%). The compound was synthesized accordingly to ref (91). ^1^H NMR (300 MHz, CDCl_3_) δ (ppm): 8.26 (dt, ^3^*J* = 7.9, ^4^*J* = 1.2 Hz, 1H), 7.46–7.22 (m, 4H), 7.04–6.83 (m, 3H), 5.70 (s, 2H), 4.06 (s, 3H). ^13^C NMR (75 MHz, CDCl_3_) δ (ppm): 162.97 (d, ^1^*J*_C–F_ = 247.4 Hz), 162.95, 140.54, 138.09 (d, ^3^*J*_C–F_ = 7.1 Hz), 135.28, 130.46 (d, ^3^*J*_C–F_ = 8.2 Hz), 127.27, 124.06, 123.40, 122.72 (d, ^4^*J*_C–F_ = 3.0 Hz), 122.38, 115.12 (d, ^2^*J*_C–F_ = 21.1 Hz), 114.18 (d, ^2^*J*_C–F_ = 22.2 Hz), 109.78, 53.37 (d, *J*^5^_C_–_F_ = 2.0 Hz), 52.13. LC–MS (ESI+) *m*/*z*: [M + H]^+^ calcd for C_16_H_13_FN_2_O_2_, 285.1034; found, 285.1.

#### 1-(3-Fluorobenzyl)-1*H*-indazole-3-carboxylic Acid (**47**)

To a solution of (**50**) (270 mg, 1.00 mmol, 1.0 equiv) in THF (10 mL) and water (10 mL) was added LiOH (68.4 mg, 2.80 mmol, 3.0 equiv). The mixture was stirred for 16 h before the organic solvent was removed under reduced pressure. The remaining aqueous phase was acidified (pH ≈ 1–2) with HCl (2 M) and extracted with DCM (3 × 10 mL). The combined organic layers were dried over Na_2_SO_4_, filtered, and removed under reduced pressure. The title compound was obtained as a white solid (244 mg, 0.90 mmol, 95%). ^1^H NMR (300 MHz, MeOD) δ (ppm): 8.19 (dt, ^3^*J* = 8.2, ^4^*J* = 1.0 Hz, 1H), 7.62 (dt, ^3^*J* = 8.5, ^4^*J* = 0.9 Hz, 1H), 7.46 (ddd, ^3^*J* = 8.4, ^3^*J* = 6.9, ^4^*J* = 1.2 Hz, 1H), 7.09–6.96 (m, 2H), 5.75 (s, 1H). LC–MS (ESI+) *m*/*z*: [M + H]^+^ calcd for C_15_H_11_FN_2_O_2_, 271.0877; found, 271.1.

#### Ethyl 2-Ethyl-2-(1-(3-fluorobenzyl)-1*H*-indazole-3-carboxamido)butanoate (**19**)

To a solution of (**47**) (30.0 mg, 111 μmol, 1.0 equiv) and HATU (42.0 mg, 111 μmol, 1.0 equiv) in ACN (2 mL) was added DIPEA (57.0 μL, 43.0 mg, 333 μmol, 3.0 equiv). The solution was stirred for 20 min before (**13**) (26.0 mg, 133 μmol, 1.2 equiv) was added. The reaction was stirred for 16 h. To prepare the mixture for purification H_2_O was added (2 mL), the mixture filtered and then purified by reversed-phase preparative HPLC (40–95% ACN + 0.1% TFA/H_2_O + 0.1% TFA). The title compound was obtained as a clear oil (9.56 mg, 23.2 μmol, 21%) after lyophilization. ^1^H NMR (600 MHz, MeOD) δ (ppm): 8.21 (d, ^3^*J* = 8.2 Hz, 1H), 7.60–7.55 (m, 1H), 7.46–7.40 (m, 1H), 7.36–7.26 (m, 2H), 7.08–6.96 (m, 3H), 5.74 (s, 1H), 4.29 (q, ^3^*J* = 7.1 Hz, 1H), 2.46 (dq, ^2^*J* = 14.7, ^3^*J* = 7.5 Hz, 2H), 2.01 (dq, ^2^*J* = 14.6, ^3^*J* = 7.4 Hz, 2H), 1.31 (t, ^3^*J* = 7.2 Hz, 2H), 0.85 (t, ^3^*J* = 7.4 Hz, 5H). ^13^C NMR (151 MHz, MeOD) δ (ppm): 175.02, 164.38 (d, ^1^*J*_C–F_ = 245.5 Hz), 163.38, 142.50, 140.59 (d, ^3^*J*_C–F_ = 7.2 Hz), 138.76, 131.68 (d, ^3^*J*_C–F_ = 8.3 Hz), 128.32, 124.17 (d, ^4^*J*_C–F_ = 3.0 Hz), 124.08, 124.04, 123.13, 115.74 (d, ^2^*J*_C–F_ = 21.4 Hz), 115.18 (d, ^2^*J*_C–F_ = 22.3 Hz), 111.20, 66.72, 62.93, 53.56, 28.89, 14.54, 8.63. HR-MS (ESI+) *m*/*z*: [M + H]^+^ calcd for C_23_H_26_FN_3_O_3_, 412.2031; found, 412.2034.

#### (9*H*-Fluoren-9-yl)methyl (13-Ethyl-2,2-dimethyl-4,12-dioxo-3,8-dioxa-5,11-diazapentadecan-13-yl)carbamate (**21a**)

The protected amino acid (**20**) (200 mg, 0.57 mmol, 1.0 equiv) was activated with HATU (215 mg, 0.57 mmol, 1.0 equiv) and DIPEA (148 μL, 112 mg, 0.85 mmol, 1.5 equiv) in DMF (6 mL) and stirred for 20 min. Then the amine component *tert*-butyl (2-(2-aminoethoxy)ethyl)carbamate (127 mg, 0.62 mmol, 1.1 equiv) was added in DMF (2 mL). The reaction was stirred for 1 h, and the solvent was evaporated under reduced pressure. The residue was taken up in EtOAc (30 mL). The organic solvent layer was washed with NaHCO_3_ solution (2 × 20 mL, 1 M) and sat. NaCl solution (20 mL), dried over Na_2_SO_4_, filtered, and removed under reduced pressure. The title compound was obtained as a white powder (255 mg, 47.3 μmol, 83%). ^1^H NMR (300 MHz, CDCl_3_) δ (ppm): 7.76 (d, ^3^*J* = 7.6 Hz, 2H), 7.61 (d, ^3^*J* = 7.8 Hz, 2H), 7.39 (td, ^3^*J* = 7.6, ^4^*J* = 0.8 Hz, 2H), 7.31 (td, ^3^*J* = 7.4, ^4^*J* = 1.2 Hz, 2H), 6.27 (br s, 1H), 6.16 (br s, 1H), 4.90 (br s, 1H), 4.38 (d, ^3^*J* = 7.0 Hz, 2H), 4.22 (t, ^3^*J* = 6.8 Hz, 1H), 3.58–3.42 (m, 6H), 3.29 (s, 1H), 2.55–2.31 (m, 2H), 1.68–1.49 (m, 2H), 1.44 (s, 9H), 0.78 (t, ^3^*J* = 7.0 Hz, 6H). ^13^C NMR (75 MHz, CDCl_3_) δ (ppm): 172.98, 156.18, 154.31, 144.11, 141.45, 127.77, 127.18, 125.20, 120.09, 79.75, 70.29, 69.65, 66.25, 64.33, 47.45, 40.62, 39.75, 29.17, 28.52, 8.20. LC–MS (ESI+) *m*/*z*: [M + Na]^+^ calcd for C_30_H_41_N_3_O_6_, 562.2888; found, 562.3.

#### (9*H*-Fluoren-9-yl)methyl (16-Ethyl-2,2-dimethyl-4,15-dioxo-3,8,11-trioxa-5,14-diazaoctadecan-16-yl)carbamate (**21b**)

The protected amino acid (**20**) (200 mg, 0.57 mmol, 1.0 equiv) was activated with HATU (215 mg, 0.57 mmol, 1.0 equiv) and DIPEA (148 μL, 113 mg, 0.85 mmol, 1.5 equiv) in DMF (6 mL) and stirred for 20 min. Then the amine component *tert*-butyl (2-(2-(2-aminoethoxy)ethoxy)ethyl)carbamate (155 mg, 0.62 mmol, 1.1 equiv) was added in DMF (2 mL). The reaction was stirred for 1 h, and the solvent was evaporated under reduced pressure. The residue was taken up in EtOAc (30 mL). The organic solvent layer was washed with NaHCO_3_ solution (1 M, 2 × 20 mL) and sat. NaCl solution (20 mL), dried over Na_2_SO_4_, filtered, and removed under reduced pressure. The title compound was obtained as a white powder (315 mg, 53.9 μmol, 95%). ^1^H NMR (300 MHz, CDCl_3_) δ (ppm): 7.75 (dd, ^3^*J* = 7.6, 1.1 Hz, 2H), 7.61 (d, ^3^*J* = 7.4 Hz, 2H), 7.38 (td, ^3^*J* = 7.5, ^4^*J* = 1.2 Hz, 2H), 7.30 (td, ^3^*J* = 7.4, ^4^*J* = 1.2 Hz, 2H), 6.65–6.13 (m, 1H), 4.34 (d, ^3^*J* = 7.2 Hz, 2H), 4.21 (t, ^3^*J* = 6.9 Hz, 1H), 3.75–3.43 (m, 10H), 3.30 (t, ^3^*J* = 5.2 Hz, 1H), 2.58–2.31 (m, 2H), 1.75–1.52 (m, 2H), 1.44 (s, 9H), 0.77 (t, ^3^*J* = 7.4 Hz, 6H). ^13^C NMR (75 MHz, CDCl_3_) δ (ppm): 173.11, 156.30, 154.14, 144.16, 141.41, 127.71, 127.15, 125.24, 120.03, 79.79, 70.56, 70.33, 69.91, 66.18, 64.35, 47.43, 39.93, 39.11, 29.02, 28.51, 8.23. LC–MS (ESI+) *m*/*z*: [M + Na]^+^ calcd for C_32_H_45_N_3_O_7_, 606.3150; found, 606.3.

#### (9*H*-Fluoren-9-yl)methyl *tert*-Butyl (14-Ethyl-13-oxo-3,6,9-trioxa-12-azahexadecane-1,14-diyl)dicarbamate (**21c**)

The protected amino acid (**20**) (200 mg, 0.57 mmol, 1.0 equiv) was activated with HATU (215 mg, 0.57 mmol, 1.0 equiv) and DIPEA (148 μL, 113 mg, 0.85 mmol, 1.5 equiv) in DMF (6 mL) and stirred for 20 min. Then the amine component *tert*-butyl (14-amino-14-ethyl-13-oxo-3,6,9-trioxa-12-azahexadecyl)carbamate (182 mg, 0.62 mmol, 1.1 equiv) was added in DMF (2 mL). The reaction was stirred for 1 h, and the solvent was evaporated under reduced pressure. The residue was taken up in EtOAc (30 mL). The organic solvent layer was washed with NaHCO_3_ solution (1 M, 2 × 20 mL) and sat. NaCl solution (20 mL), dried over Na_2_SO_4_, filtered, and removed under reduced pressure. The title compound was obtained as a white powder (339 mg, 54.0 μmol, 95%). ^1^H NMR (300 MHz, CDCl_3_) δ (ppm): 7.76 (d, ^3^*J* = 7.3 Hz, 2H), 7.62 (d, ^3^*J* = 7.4 Hz, 2H), 7.39 (td, ^3^*J* = 7.8, ^4^*J* = 1.2 Hz, 2H), 7.30 (td, ^3^*J* = 7.4, ^4^*J* = 1.3 Hz, 2H), 6.67–6.13 (m, 2H), 5.07 (br s, 1H), 4.35 (d, ^3^*J* = 7.0 Hz, 2H), 4.22 (t, ^3^*J* = 6.9 Hz, 1H), 3.68–3.46 (m, 12H), 3.30 (t, ^3^*J* = 5.2 Hz, 2H), 2.57–2.31 (m, 2H), 1.70–1.50 (m, 2H), 1.44 (s, 9H), 0.93–0.62 (m, 6H). ^13^C NMR (75 MHz, CDCl_3_) δ (ppm): 172.98, 156.19, 154.16, 144.17, 141.43, 127.73, 127.16, 125.25, 120.05, 79.58, 70.57, 70.55, 70.37, 70.33, 70.24, 69.88, 66.19, 64.35, 47.45, 40.72, 39.77, 29.13, 28.54, 8.25. LC–MS (ESI+) *m*/*z*: [M + Na]^+^ calcd for C_34_H_49_N_3_O_8_, 650.3412; found, 650.4.

#### (9*H*-Fluoren-9-yl)methyl *tert*-Butyl (17-Ethyl-16-oxo-3,6,9,12-tetraoxa-15-azanonadecane-1,17-diyl)dicarbamate (**21d**)

The protected amino acid (**20**) (200 mg, 0.57 mmol, 1.0 equiv) was activated with HATU (215 mg, 0.57 mmol, 1.0 equiv) and DIPEA (148 μL, 113 mg, 0.85 mmol, 1.5 equiv) in DMF (6 mL) and stirred for 20 min. Then, the amine component *tert*-butyl (17-amino-17-ethyl-16-oxo-3,6,9,12-tetraoxa-15-azanonadecyl)carbamate (209 mg, 0.62 mmol, 1.1 equiv) was added in DMF (2 mL). The reaction was stirred for 1 h, and the solvent was evaporated under reduced pressure. The residue was taken up in EtOAc (30 mL). The organic solvent layer was washed with NaHCO_3_ solution (1 M, 2 × 20 mL) and sat. NaCl solution (20 mL), dried over Na_2_SO_4_, filtered, and removed under reduced pressure. The title compound was obtained as a white powder (372 mg, 55.5 μmol, 97%). ^1^H NMR (300 MHz, CDCl_3_) δ (ppm): 7.76 (d, ^3^*J* = 7.4 Hz, 2H), 7.62 (d, ^3^*J* = 7.4 Hz, 2H), 7.39 (td, ^3^*J* = 7.5, ^4^*J* = 1.1 Hz, 2H), 7.30 (td, ^3^*J* = 7.4, ^4^*J* = 1.3 Hz, 2H), 6.51 (br s, 1H), 6.31 (br s, 1H), 5.12 (br s, 1H), 4.44–4.28 (m, 2H), 4.22 (t, ^3^*J* = 6.9 Hz, 1H), 3.72–3.47 (m, 16H), 3.36–3.25 (m, 2H), 2.60–2.28 (m, 2H), 1.78–1.49 (m, 2H), 1.43 (s, 9H), 0.77 (t, ^3^*J* = 7.5 Hz, 6H). ^13^C NMR (75 MHz, CDCl_3_) δ (ppm): 172.92, 156.15, 154.17, 144.17, 141.43, 127.73, 127.16, 125.25, 120.05, 79.37, 70.70, 70.64, 70.62, 70.58, 70.44, 70.35, 70.30, 69.86, 66.19, 64.35, 47.45, 40.57, 39.76, 29.19, 28.55, 8.27. LC–MS (ESI+) *m*/*z*: [M + Na]^+^ calcd for C_36_H_53_N_3_O_9_, 694.3674; found, 694.3.

#### *tert*-Butyl (*R*)-(2-(2-(2-(5-(2,5-Bis(trifluoromethyl)phenyl)-2-methyl-1-((tetrahydrofuran-2-yl)methyl)-1*H*-pyrrole-3-carboxamido)-2-ethylbutanamido)ethoxy)ethyl)carbamate (**22a**)

The Fmoc-protected amine (**21a**) (45.0 mg, 83.4 μmol, 1 equiv) was dissolved in anhydrous DMF (1 mL) and DBU (18.0 μL, 19.1 mg, 125 μmol, 1.5 equiv). After 30 min, HOAt (18.0 mg, 133 μmol, 1.6 equiv) was added to the mixture and stirred for another 10 min. In a separate flask, (**42**) (35.1 mg, 83.4 μmol, 1.1 equiv) was dissolved with HATU (32.0 mg, 83.4 μmol, 1.1 equiv) and DIPEA (42.0 μL, 32.0 mg, 250 μmol, 3.0 equiv) in anhydrous DMF (1 mL). The solution was stirred for 15 min and then combined with the deprotected amine mixture. The progress of the reaction was monitored via LCMS. After 2 h of stirring another portion of (**42**) (35.1 mg, 83.4 μmol, 1.1 equiv) was dissolved with HATU (32 mg, 83.4 μmol, 1.1 equiv) and DIPEA (42.0 μL, 32.0 mg, 250 μmol, 3.0 equiv) in anhydrous DMF (1 mL). The solution was stirred for 15 min and then combined with the deprotected amine mixture. The progress of the reaction was monitored via LCMS. The solvent was removed under reduced pressure, the mixture taken up in ACN/H_2_O (v/v 1/1), filtered, and purified by reversed-phase preparative HPLC (40–95% ACN/H_2_O). The title compound was obtained as an off-white powder (5.40 mg, 7.49 μmol, 9%) after lyophilization. Side product **S53** isolated with 77% yield (see Figure S24). ^1^H NMR (600 MHz, CDCl_3_) δ (ppm): 7.95–7.62 (m, 3H), 6.94 (N*H*, s, 1H), 6.46 (s, 1H), 6.37 (N*H*, s, 1H), 4.95 (N*H*, s, 1H), 4.11–3.37 (m, 11H), 3.32–3.27 (m, 2H), 2.62 (s, 5H), 1.92 (s, 1H), 1.73–1.62 (m, 2H), 1.43 (s, 9H), 1.34–1.26 (m, 1H), 0.84 (t, ^3^*J* = 7.4 Hz, 6H). ^13^C NMR (151 MHz, CDCl_3_) δ (ppm): 173.79, 165.01, 156.07, 134.13, 133.93, 132.91, 132.61, 125.30, 123.95, 115.88, 109.28, 79.40, 70.20, 69.59, 67.66, 65.05, 49.06, 40.41, 39.61, 29.22, 29.02, 28.40, 25.42, 11.73, 8.29. LC–MS (ESI+) *m*/*z*: [M + H]^+^ calcd for C_34_H_46_F_6_N_4_O_6_, 721.3394; found, 720.8.

#### *tert*-Butyl (*R*)-(1-(5-(2,5-Bis(trifluoromethyl)phenyl)-2-methyl-1-((tetrahydrofuran-2-yl)methyl)-1*H*-pyrrol-3-yl)-3,3-diethyl-1,4-dioxo-8,11-dioxa-2,5-diazatridecan-13-yl)carbamate (**22b**)

The Fmoc-protected amine (**21b**) (64.9 mg, 111 μmol, 1.0 equiv) was dissolved in anhydrous DMF (0.5 mL) and DBU (24.2 μL, 25.4 mg, 167 μmol, 1.5 equiv). After 30 min, HOAt (27.6 mg, 178 μmol, 1.6 equiv) was added to the mixture and stirred for another 10 min. In a separate flask, (**42**) (51.5 mg, 122 μmol, 1.1 equiv) was dissolved with HATU (46.4 mg, 122 μmol, 1.1 equiv) and DIPEA (75.4 μL, 57.3 mg, 444 μmol, 4.0 equiv) in anhydrous DMF (0.5 mL). The solution was stirred for 15 min, then combined with the deprotected amine mixture, heated to 40 °C, and stirred for 14 days. The solvent was removed under reduced pressure, and the was mixture taken up in ACN/H_2_O (v/v 1/1), filtered, and purified by reversed-phase preparative HPLC (40–95% ACN/H_2_O). The title compound was obtained as an off-white powder (47.7 mg, 62.0 μmol, 56%) after lyophilization. ^1^H NMR (600 MHz, MeOD) δ (ppm): 8.12–7.76 (m, 3H), 6.44 (s, 1H), 4.15–3.74 (m, 2H), 3.64–3.38 (m, 13H), 3.21 (t, ^3^*J* = 5.7 Hz, 2H), 2.59 (s, 3H), 2.41 (dq, ^2^*J* = 15.4, ^3^*J* = 7.5 Hz, 2H), 2.00–1.92 (m, 1H), 1.87 (dq, ^2^*J* = 14.5, ^3^*J* = 7.1 Hz, 2H), 1.82–1.65 (m, 2H), 1.42 (s, 9H), 1.39–1.33 (m, 1H), 0.80 (t, ^3^*J* = 7.4 Hz, 6H). ^13^C NMR (151 MHz, MeOD) δ (ppm): 175.88, 167.04, 158.43, 135.35, 135.15, 134.96, 134.28, 133.64, 128.65, 128.16, 126.95, 125.71, 125.54, 123.91, 123.72, 116.76, 110.24, 80.10, 79.04, 71.30, 71.06, 70.61, 68.62, 66.03, 50.13, 41.23, 40.65, 29.99, 28.82, 28.76, 26.38, 11.95, 8.46. HR-MS (ESI+) *m*/*z*: [M + H]^+^ calcd for C_36_H_50_F_6_N_4_O_7_, 764.3584; found, 764.3595.

#### *tert*-Butyl (*R*)-(1-(5-(2,5-Bis(trifluoromethyl)phenyl)-2-methyl-1-((tetrahydrofuran-2-yl)methyl)-1*H*-pyrrol-3-yl)-3,3-diethyl-1,4-dioxo-8,11,14-trioxa-2,5-diazahexadecan-16-yl)carbamate (**22c**)

The Fmoc-protected amine (**21c**) (14.1 mg, 22.4 μmol, 0.9 equiv) was dissolved in anhydrous DMF (0.3 mL) and DBU (9.30 μL, 9.90 mg, 64.8 μmol, 1.7 equiv). After 30 min, HOAt (8.33 mg, 64.8 μmol, 1.7 equiv) was added to the mixture and stirred for another 10 min. In a separate flask, (**42**) (18.3 mg, 43.4 μmol, 1.0 equiv) was dissolved in anhydrous DMF (0.3 mL) together with HATU (16.5 mg, 43.4 μmol, 1.0 equiv) and DIPEA (37.0 μL, 28.0 mg, 217 μmol, 5.0 equiv). The solution was stirred for 15 min, then combined with the deprotected amine mixture, heated to 45 °C, and stirred for min. Four days. The solvent was removed under reduced pressure, and the mixture was taken up in ACN/H_2_O (v/v 1/1), filtered, and purified by reversed-phase preparative HPLC (30–95% ACN/H_2_O). The title compound was obtained as an off-white powder (16.9 mg, 20.9 μmol, 48%) after lyophilization. ^1^H NMR (600 MHz, CDCl_3_) δ (ppm): 7.95–7.61 (m, 3H), 7.13 (s, 1H), 6.56 (s, 1H), 6.39 (s, 1H), 5.29–4.92 (m, 1H), 4.08–3.20 (m, 19H), 3.37–3.19 (m, 2H), 2.70 (dt, ^3^*J* = 14.6, 7.4 Hz, 2H), 2.63 (s, 3H), 2.00–1.56 (m, 5H), 1.44 (s, 9H), 1.34–1.23 (m, 1H), 0.81 (t, ^3^*J* = 7.4 Hz, 6H). HR-MS (ESI+) *m*/*z*: [M + H]^+^ calcd for C_38_H_54_F_6_N_4_O_8_, 809.3919; found, 809.3978.

#### *tert*-Butyl (2-(2-(2-(5-(4-Chlorophenyl)-1-(2,4-dichlorophenyl)-4-methyl-1*H*-pyrazole-3-carboxamido)-2-ethylbutanamido)ethoxy)ethyl)carbamate (**23a**)

The Fmoc-protected amine (**21a**) (25.0 mg, 46.3 μmol, 1 equiv) was dissolved in anhydrous ACN/DCM (0.2 mL, v/v 1/1) and DBU (10.0 μL, 11 mg, 69.5 μmol, 1.5 equiv). After 30 min, HOAt (10.0 mg, 74.1 μmol, 1.6 equiv) was added to the mixture and stirred for another 10 min. The compound (**43**) (21.0 mg, 55.6 μmol, 1.2 equiv) was dissolved together with HATU (21.0 mg, 55.6 μmol, 1.2 equiv) and DIPEA (27.5 μL, 20.9 mg, 162 μmol, 3.5 equiv) in anhydrous ACN/DCM (0.2 mL, v/v 1/1) and stirred for 20 min. The activated acid was added to the amine component, and the reaction was followed via LC–MS for 3 h. Another portion of (**43**) (21.0 mg, 55.6 μmol, 1.2 equiv) was dissolved with HATU (21.0 mg, 55.6 μmol, 1.2 equiv) and DIPEA (27.5 μL, 20.9 mg, 162 μmol, 3.5 equiv) in anhydrous ACN/DCM (0.2 mL, v/v 1/1) and added to the mixture to bring unreacted amine to reaction. After 15 h, the solvent was evaporated, and the mixture was taken up in ACN/H_2_O (v/v 1/1), filtered, and purified by reversed-phase preparative HPLC (40–95% ACN + 0.1% TFA/H_2_O + 0.1% TFA). The title compound was obtained as a white powder (21.2 mg, 31.0 μmol, 65%) after lyophilization. ^1^H NMR (600 MHz, CDCl_3_) δ (ppm): 7.89 (s, 1H), 7.40 (d, ^4^*J* = 2.1 Hz, 1H), 7.33–7.26 (m, 4H), 7.09–7.03 (m, 2H), 6.53 (t, ^3^*J* = 5.5 Hz, 1H), 4.98 (s, 1H), 3.59–3.45 (m, 6H), 3.28 (q, ^3^*J* = 5.1 Hz, 2H), 2.53 (dq, ^2^*J* = 14.8, ^3^*J* = 7.4 Hz, 2H), 2.35 (s, 3H), 1.81 (dq, ^2^*J* = 14.6, ^3^*J* = 7.3 Hz, 2H), 1.43 (s, 9H), 0.88 (t, ^3^*J* = 7.4 Hz, 6H). ^13^C NMR (151 MHz, CDCl_3_) δ (ppm): 173.36, 162.25, 156.15, 145.27, 143.12, 136.19, 135.90, 134.97, 133.02, 130.99, 130.84, 130.36, 129.00, 127.91, 127.56, 117.61, 79.49, 70.30, 69.75, 65.14, 40.58, 39.71, 28.68, 28.56, 9.62, 8.38. HR-MS (ESI+) *m*/*z*: [M + Na]^+^ calcd for C_32_H_40_Cl_3_N_5_O_5_, 702.1987; found, 702.1998.

#### *tert*-Butyl (1-(5-(4-Chlorophenyl)-1-(2,4-dichlorophenyl)-4-methyl-1*H*-pyrazol-3-yl)-3,3-diethyl-1,4-dioxo-8,11-dioxa-2,5-diazatridecan-13-yl)carbamate (**23b**)

The protected amine (**21b**) (48.9 mg, 83.8 μmol, 1.0 equiv) was dissolved in anhydrous ACN/DCM (1 mL, v/v 1/1) and DBU (18.0 μL, 19.1 mg, 126 μmol, 1.5 equiv). After 30 min, HOAt (18.1 mg, 135 μmol, 1.6 equiv) was added to the mixture and stirred for another 10 min. The compound (**43**) (38.4 mg, 101 μmol, 1.2 equiv) was activated with HATU (38.2 mg, 101 μmol, 1.2 equiv) and DIPEA (43.0 μL, 32.4 mg, 251 μmol, 3.0 equiv) in anhydrous ACN/DCM (1 mL, v/v 1/1) and stirred for 20 min. The activated acid was added to the amine component, and the reaction was followed via LC–MS for 3 h. Another portion of (**43**) (16.0 mg, 42.1 μmol, 0.5 equiv) was activated with HATU (16.0 mg, 42.1 μmol, 0.5 equiv) and DIPEA (21.5 μL, 16.2 mg, 126 μmol, 1.5 equiv) in anhydrous ACN/DCM (1 mL, v/v 1/1) for 20 min and added to the reaction mixture to bring unreacted amine to reaction. After 15 h, the solvent was evaporated, and the mixture taken up in ACN/H_2_O (v/v 1/1), filtered, and purified by reversed-phase preparative HPLC (40–95% ACN + 0.1% TFA/H_2_O + 0.1% TFA). The title compound was obtained as a highly viscous oil (33.4 mg, 46.1 μmol, 55%) after lyophilization. ^1^H NMR (600 MHz, MeOD) δ (ppm): 7.57 (d, ^4^*J* = 2.2 Hz, 1H), 7.55–7.52 (m, 1H), 7.45 (dd, ^3^*J* = 8.5, ^4^*J* = 2.3 Hz, 1H), 7.39–7.34 (m, 2H), 7.24–7.18 (m, 2H), 3.64–3.53 (m, 6H), 3.49 (t, ^3^*J* = 5.7 Hz, 2H), 3.44 (t, ^3^*J* = 5.5 Hz, 2H), 3.20 (t, ^3^*J* = 5.7 Hz, 2H), 2.53 (dq, ^2^*J* = 14.7, ^3^*J* = 7.4 Hz, 2H), 1.87 (dq, ^2^*J* = 14.5, ^3^*J* = 7.3 Hz, 2H), 1.43 (s, 9H), 0.82 (t, ^3^*J* = 7.4 Hz, 6H). ^13^C NMR (151 MHz, MeOD) δ (ppm): 175.12, 163.36, 158.44, 146.33, 144.77, 137.40, 137.30, 136.19, 134.17, 132.56, 132.49, 131.06, 129.88, 129.22, 128.71, 118.23, 80.10, 71.30, 71.28, 71.06, 70.56, 66.47, 41.24, 40.69, 29.21, 28.77, 9.60, 8.53. HR-MS (ESI+) *m*/*z*: [M + Na]^+^ calcd for C_34_H_44_Cl_3_N_5_O_6_, 746.2249; found, 746.2259.

#### *tert*-Butyl (1-(5-(4-Chlorophenyl)-1-(2,4-dichlorophenyl)-4-methyl-1*H*-pyrazol-3-yl)-3,3-diethyl-1,4-dioxo-8,11,14-trioxa-2,5-diazahexadecan-16-yl)carbamate (**23c**)

The protected amine (**21c**) (42.4 mg, 67.5 μmol, 1.0 equiv) was dissolved in anhydrous ACN/DCM (1 mL, v/v 1/1) and DBU (14.5 μL, 15.4 mg, 101 μmol, 1.5 equiv). After 30 min, HOAt (14.6 mg, 108 μmol, 1.6 equiv) was added to the mixture and stirred for another 10 min. The compound (**43**) (31.0 mg, 80.9 μmol, 1.2 equiv) was activated with HATU (31.6 mg, 80.9 μmol, 1.2 equiv) and DIPEA (34.4 μL, 26.0 mg, 202 μmol, 3.0 equiv) in anhydrous ACN/DCM (1 mL, v/v 1/1) and stirred for 20 min. The activated acid was added to the amine component, and the reaction was followed via LC–MS for 3 h. Another portion of (**43**) (12.9 mg, 33.7 μmol, 0.5 equiv) was activated with HATU (12.8 mg, 33.7 μmol, 0.5 equiv) and DIPEA (11.5 μL, 8.7 mg, 67.4 μmol, 1.5 equiv) in anhydrous ACN/DCM (1 mL, v/v 1/1) for 20 min and added to the reaction mixture to bring unreacted amine to reaction. After 15 h, the solvent was evaporated, and the mixture was taken up in ACN/H_2_O (v/v 1/1), filtered, and purified by reversed-phase preparative HPLC (40–95% ACN + 0.1% TFA/H_2_O + 0.1% TFA). The title compound was obtained as a highly viscous oil (25.2 mg, 32.8 μmol, 49%) after lyophilization. ^1^H NMR (600 MHz, CDCl_3_) δ (ppm): 8.07 (br s, 1H), 7.38 (d, ^4^*J* = 2.2 Hz, 1H), 7.32 (d, ^3^*J* = 8.4 Hz, 1H), 7.29–7.26 (m, 3H), 6.48 (br s, 1H), 5.01 (br s, 1H), 3.65–3.55 (m, 10H), 3.55–3.49 (m, 4H), 3.30 (s, 2H), 2.68–2.55 (m, 2H), 2.34 (s, 3H), 1.80–1.68 (m, 2H), 1.44 (s, 9H), 0.85 (t, ^3^*J* = 7.4 Hz, 6H). ^13^C NMR (151 MHz, CDCl_3_) δ (ppm): 173.27, 161.98, 156.15, 145.41, 142.95, 136.24, 135.77, 134.87, 132.98, 130.95, 130.86, 130.28, 128.95, 127.85, 127.65, 117.46, 79.41, 70.62, 70.41, 70.36, 70.31, 69.96, 65.19, 40.50, 39.76, 28.90, 28.56, 9.62, 8.46. HR-MS (ESI+) *m*/*z*: [M + Na]^+^ calcd for C_36_H_48_Cl_3_N_5_O_7_, 790.2512; found, 790.2519.

#### *tert*-Butyl (1-(5-(4-Chlorophenyl)-1-(2,4-dichlorophenyl)-4-methyl-1*H*-pyrazol-3-yl)-3,3-diethyl-1,4-dioxo-8,11,14,17-tetraoxa-2,5-diazanonadecan-19-yl)carbamate (**23d**)

The protected amine (**21d**) (62.9 mg, 93.6 μmol, 1.0 equiv) was dissolved in anhydrous ACN/DCM (1 mL, v/v 1/1) and DBU (20.2 μL, 21.4 mg, 140 μmol, 1.5 equiv). After 30 min, HOAt (20.2 mg, 150 μmol, 1.6 equiv) was added to the mixture and stirred for another 10 min. The compound (**43**) (43.0 mg, 112 μmol, 1.2 equiv) was activated with HATU (43.0 mg, 112 μmol, 1.2 equiv) and DIPEA (48.0 μL, 36.2 mg, 281 μmol, 3.0 equiv) in anhydrous ACN/DCM (1 mL, v/v 1/1) and stirred for 20 min. The activated acid was added to the amine component, and the reaction was followed via LC–MS for 3 h. Another portion of (**43**) (18.0 mg, 47.2 μmol, 0.5 equiv) was activated with HATU (18.0 mg, 47.2 μmol, 0.5 equiv) and DIPEA (24.0 μL, 18.2 mg, 141 μmol, 1.5 equiv) in anhydrous ACN/DCM (1 mL, v/v 1/1) for 20 min and added to the reaction mixture to bring unreacted amine to reaction. After 15 h, the solvent was evaporated, and the mixture was taken up in ACN/H_2_O (v/v 1/1), filtered, and purified by reversed-phase preparative HPLC (40–95% ACN + 0.1% TFA/H_2_O + 0.1% TFA). The title compound was obtained as a highly viscous oil (40.8 mg, 50.2 μmol, 79%) after lyophilization. ^1^H NMR (600 MHz, CDCl_3_) δ (ppm): 8.13 (br s, 1H), 7.38 (d, ^4^*J* = 2.2 Hz, 1H), 7.35–7.31 (m, 1H), 7.30–7.25 (m, 4H), 7.08–7.03 (m, 2H), 6.64 (br s, 1H), 5.06 (br s, 1H), 3.66–3.60 (m, 12H), 3.57 (t, ^3^*J* = 5.0 Hz, 2H), 3.55–3.50 (m, 4H), 3.31 (t, ^3^*J* = 5.2 Hz, 2H), 2.64 (dq, ^2^*J* = 14.8, ^3^*J* = 7.4 Hz, 2H), 2.35 (s, 3H), 1.75 (dq, ^2^*J* = 14.5, ^3^*J* = 7.2 Hz, 2H), 1.44 (s, 9H), 0.85 (t, ^3^*J* = 7.3 Hz, 6H). ^13^C NMR (151 MHz, CDCl_3_) δ (ppm): 173.28, 161.99, 156.18, 145.45, 142.96, 136.27, 135.78, 134.88, 133.00, 130.97, 130.88, 130.30, 128.96, 127.86, 127.68, 117.46, 79.38, 70.75, 70.70, 70.68, 70.63, 70.47, 70.39, 70.37, 69.99, 65.25, 40.69, 39.77, 28.97, 28.58, 9.64, 8.49. HR-MS (ESI+) *m*/*z*: [M + Na]^+^ calcd for C_38_H_52_Cl_3_N_5_O_8_, 834.2774; found, 834.2791.

#### *tert*-Butyl (1-(8-Chloro-1-(2,4-dichlorophenyl)-1,4,5,6-tetrahydrobenzo[6,7]cyclohepta[1,2-*c*]pyrazol-3-yl)-3,3-diethyl-1,4-dioxo-8,11-dioxa-2,5-diazatridecan-13-yl)carbamate (**24**)

The Fmoc-protected amine (**21b**) (70.4 mg, 121 μmol, 1.2 equiv) was dissolved in anhydrous DMF (1 mL) and DBU (23.0 μL, 24.4 mg, 516 μmol, 1.5 equiv). After 30 min, HOAt (22.0 mg, 161 μmol, 1.6 equiv) was added to the mixture and stirred for another 10 min. In a separate flask, (**44**) (41.0 mg, 101 μmol, 1.0 equiv) was activated with HATU (46.7 mg, 121 μmol, 1.2 equiv) and DIPEA (65.0 μL, 50.7 mg, 503 μmol, 5 equiv) in anhydrous DMF (1 mL) and stirred for 20 min. The activated acid was added to the amine component, and the reaction was followed via LC–MS for 3 h. The solvent was evaporated, and the mixture taken up in ACN/H_2_O (v/v 1/1), filtered, and purified by reversed-phase preparative HPLC (50–95% ACN + 0.1% TFA/H_2_O + 0.1% TFA). The title compound was obtained as a white powder (33.4 mg, 46.1 μmol, 55%) after lyophilization. ^1^H NMR (600 MHz, MeOD) δ (ppm): 7.69 (d, ^3^*J* = 8.5 Hz, 1H), 7.61 (d, ^4^*J* = 2.3 Hz, 1H), 7.57 (dd, ^3^*J* = 8.5, ^4^*J* = 2.3 Hz, 1H), 7.39 (d, ^4^*J* = 2.2 Hz, 1H), 7.08 (dd, ^3^*J* = 8.3, ^4^*J* = 2.2 Hz, 1H), 6.72 (d, ^3^*J* = 8.3 Hz, 1H), 3.62–3.54 (m, 6H), 3.49 (t, ^3^*J* = 5.7 Hz, 2H), 3.44 (t, ^3^*J* = 5.5 Hz, 2H), 3.20 (t, ^3^*J* = 5.7 Hz, 2H), 2.71 (t, ^3^*J* = 6.7 Hz, 2H), 2.53 (dq, ^2^*J* = 14.7, ^3^*J* = 7.4 Hz, 2H), 2.27–2.24 (m, 2H), 1.87 (dq, ^2^*J* = 14.6, ^3^*J* = 7.2 Hz, 2H), 1.43 (s, 9H), 0.83 (t, ^3^*J* = 7.4 Hz, 6H). One bridging CH_2_-group signal was not resolved (see Figure S23 for the coalescence effect). ^13^C NMR (151 MHz, MeOD) δ (ppm): 175.10, 163.33, 158.43, 145.45, 145.23, 143.95, 137.39, 137.29, 135.50, 133.65, 132.37, 131.27, 130.91, 129.62, 129.59, 129.17, 127.30, 122.86, 80.10, 71.32, 71.28, 71.07, 70.57, 66.50, 41.24, 40.70, 33.23, 32.74, 29.18, 28.77, 21.20, 8.55. HR-MS (ESI+) *m*/*z*: [M + Na]^+^ calcd for C_36_H_46_Cl_3_N_5_O_6_, 772.2406; found, 772.2411.

#### *tert*-Butyl (1-(6-(Cyclopropylmethoxy)-5-(4-fluorophenyl)pyridin-3-yl)-3,3-diethyl-1,4-dioxo-8,11-dioxa-2,5-diazatridecan-13-yl)carbamate (**25**)

The Fmoc-protected amine (**21b**) (25.0 mg, 46.3 μmol, 1.0 equiv) was dissolved in anhydrous DMF (1 mL) and DBU (10.0 μL, 10.6 mg, 69.5 μmol, 1.5 equiv). After 30 min, HOAt (10.0 mg, 74.1 μmol, 1.6 equiv) was added to the mixture and stirred for another 10 min. In a separate flask, (**45**) (13.3 mg, 46.3 μmol, 1.0 equiv) was activated with HATU (17.6 mg, 46.3 μmol, 1.0 equiv) and DIPEA (24.0 μL, 17.9 mg, 139 μmol, 3.0 equiv) in anhydrous DMF (1 mL) and stirred for 20 min. The activated acid was added to the amine component, and the reaction was followed via LC–MS for 3 h. Upon incomplete conversion, another portion of (**45**) (13.3 mg, 46.3 μmol, 1.0 equiv) was activated with HATU (17.6 mg, 46.3 μmol, 1.0 equiv) and DIPEA (24.0 μL, 17.9 mg, 139 μmol, 3.0 equiv) in anhydrous DMF (1 mL) and stirred for 20 min before adding it to the reaction mixture. After 3 h, more solvent was evaporated, and the mixture taken up in ACN/H_2_O (v/v 1/1), filtered, and purified by reversed-phase preparative HPLC (40–95% ACN/H_2_O). The title compound was obtained as a highly viscous oil (18.0 mg, 30.7 μmol, 60%) after lyophilization. The cyclopropylmethoxy moiety appeared to be acid labile. Strong acidic conditions during the reaction should be avoided. ^1^H NMR (600 MHz, MeOD) δ (ppm): 8.59 (d, ^4^*J* = 2.4 Hz, 1H), 8.09 (d, ^4^*J* = 2.4 Hz, 1H), 7.70–7.63 (m, 2H), 7.21–7.14 (m, 2H), 4.28 (dd, ^3^*J* = 7.1, ^4^*J* = 1.5 Hz, 2H), 3.60–3.52 (m, 6H), 3.48–3.42 (m, 4H), 3.18 (t, ^3^*J* = 5.7 Hz, 2H), 2.27 (dq, ^2^*J* = 14.8, ^3^*J* = 7.6 Hz, 2H), 1.98 (dq, ^2^*J* = 14.7, ^3^*J* = 7.4 Hz, 2H), 1.42 (s, 9H), 1.32–1.24 (m, 1H), 0.83 (t, ^3^*J* = 7.4 Hz, 6H), 0.61–0.54 (m, 2H), 0.37–0.32 (m, 2H). ^13^C NMR (151 MHz, MeOD) δ (ppm): 175.51, 166.76, 163.94 (d, ^1^*J*_C–F_ = 245.9 Hz), 163.81, 158.41, 146.65, 138.61, 133.47 (d, ^4^*J*_C–F_ = 3.1 Hz) 132.26 (d, ^3^*J*_C–F_ = 8.1 Hz), 132.29, 132.24, 125.53, 124.52, 116.05 (d, ^2^*J*_C–F_ = 21.7 Hz). 80.09, 72.55, 71.27, 71.25, 71.05, 70.65, 66.09, 41.21, 40.63, 28.76, 27.90, 10.93, 8.32, 3.60. HR-MS (ESI+) *m*/*z*: [M + Na]^+^ calcd for C_33_H_47_FN_4_O_7_, 653.3321; found, 653.3330.

#### *tert*-Butyl (1-(6-(4-Chlorophenyl)-5-(2-methoxyethoxy)pyrazin-2-yl)-3,3-diethyl-1,4-dioxo-8,11-dioxa-2,5-diazatridecan-13-yl)carbamate (**26**)

The Fmoc-protected amine (**21b**) (52.6 mg, 90.1 μmol, 1.0 equiv) was dissolved in anhydrous DMF (0.5 mL) and DBU (27.0 μL, 20.6 mg, 135 μmol, 1.5 equiv). After 30 min, HOAt (13.5 mg, 144 μmol, 1.6 equiv) was added to the mixture and stirred for another 10 min. In a separate flask, (**46**) (33.3 mg, 108 μmol, 1.2 equiv) was activated with HATU (41.1 mg, 108 μmol, 1.2 equiv) and DIPEA (76.5 μL, 41.1 mg, 451 μmol, 5.0 equiv) in anhydrous DMF (0.5 mL) and stirred for 20 min. The activated acid was added to the amine component, and the reaction was followed via LC–MS for 3 h. Due to incomplete conversion, EDCI (20.7 mg, 108 μmol, 1.2 equiv) was added and the solution was stirred for 24 h. The solvent was evaporated, and the mixture was taken up in ACN/H_2_O (v/v 1/1), filtered, and purified by reversed-phase preparative HPLC (40–95% ACN + 0.1% TFA/H_2_O + 0.1% TFA). The title compound was obtained as a highly viscous clear oil (29.2 mg, 44.8 μmol, 49%) after lyophilization. ^1^H NMR (600 MHz, MeOD) δ (ppm): 9.34 (s, 1H), 8.74 (s, 1H), 8.30–8.25 (m, 2H), 7.55–7.49 (m, 2H), 4.70–4.64 (m, 2H), 3.85–3.80 (m, 2H), 3.64–3.57 (m, 6H), 3.52–3.47 (m, 4H), 3.45–3.40 (m, 3H), 3.21 (t, ^3^*J* = 5.7 Hz, 2H), 2.63 (dq, ^2^*J* = 14.8, ^3^*J* = 7.5 Hz, 2H), 1.87 (dq, ^2^*J* = 14.5, ^3^*J* = 7.3 Hz, 2H), 1.42 (s, 10H), 0.79 (t, ^3^*J* = 7.4 Hz, 6H). ^13^C NMR (151 MHz, MeOD) δ (ppm): 175.03, 163.68, 160.49, 158.41, 140.91, 140.22, 138.75, 137.00, 134.72, 131.97, 129.53, 80.09, 71.55, 71.33, 71.31, 71.07, 70.57, 67.70, 66.75, 59.23, 41.24, 40.80, 29.41, 28.76, 8.60. HR-MS (ESI+) *m*/*z*: [M + Na]^+^ calcd for C_31_H_46_ClN_5_O_8_, 674.2927; found, 674.2937.

#### *tert*-Butyl (3,3-Diethyl-1-(1-(3-fluorobenzyl)-1*H*-indazol-3-yl)-1,4-dioxo-8,11-dioxa-2,5-diazatridecan-13-yl)carbamate (**27**)

The Fmoc-protected amine (**21b**) (52.0 mg, 89.0 μmol, 1.0 equiv) was dissolved in anhydrous ACN (0.5 mL) and DBU (17.0 μL, 18.0 mg, 134 μmol, 1.5 equiv). After 30 min, HOAt (19.2 mg, 142 μmol, 1.6 equiv) was added to the mixture and stirred for another 10 min. To the mixture were added DIPEA (60.4 μL, 46.0 mg, 356 μmol, 4.0 equiv) and then (**47**) (36.2 mg, 134 μmol, 1.5 equiv) together with EDCI (27.3 mg, 142.4 μmol, 1.6 equiv). The reaction was stirred for 14 h, and the conversion was followed via LC–MS. Upon incomplete conversion, more EDCI (20 mg, 104.3 μmol, 1.2 equiv) was added and the reaction was stirred for another 24 h. After completion of the reaction, the solvent was evaporated, and the mixture was taken up in ACN/H_2_O (v/v 1/1), filtered, and purified by reversed-phase preparative HPLC (40–95% ACN + 0.1% TFA/H_2_O + 0.1% TFA). The title compound was obtained as a white powder (31.8 mg, 51.8 μmol, 58%) after lyophilization. ^1^H NMR (600 MHz, MeOD) δ (ppm): 8.28–8.18 (m, 1H), 7.62–7.52 (m, 1H), 7.48–7.38 (m, 1H), 7.37–7.21 (m, 2H), 7.10–6.93 (m, 3H), 5.72 (s, 2H), 3.66–3.51 (m, 6H), 3.51–3.37 (m, 4H), 3.19 (t, ^3^*J* = 5.6 Hz, 2H), 2.59 (dq, ^2^*J* = 14.7, ^3^*J* = 7.4 Hz, 2H), 1.91 (dq, ^2^*J* = 14.5, ^3^*J* = 7.3 Hz, 2H), 1.41 (s, 9H), 0.84 (t, ^3^*J* = 7.3 Hz, 6H). ^13^C NMR (151 MHz, MeOD) δ (ppm): 175.17, 164.33 (d, ^1^*J*_C–F_ = 245.5 Hz), 163.15, 158.38, 142.46, 140.64 (d, ^2^*J*_C–F_ = 7.2 Hz), 139.07, 131.66 (d, ^2^*J*_C–F_ = 8.3 Hz), 128.24, 124.18, 124.16 (d, ^3^*J*_C–F_ = 2.9 Hz), 123.95, 123.18, 115.71 (d, ^3^*J*_C–F_ = 21.3 Hz), 115.15 (d, ^4^*J*_C–F_ = 22.4 Hz), 111.17, 80.06, 71.25, 71.02, 70.57, 66.48, 53.51, 41.19, 40.71, 29.28, 28.75, 8.57. HR-MS (ESI+) *m*/*z*: [M + Na]^+^ calcd for C_32_H_44_FN_5_O_6_, 636.3168; found, 636.3175.

#### (*R*)-5-(2,5-Bis(trifluoromethyl)phenyl)-2-methyl-*N*-(3-((2-(2-(2-((7-nitrobenzo[*c*][1,2,5]oxadiazol-4-yl)amino)ethoxy)ethoxy)ethyl)carbamoyl)pentan-3-yl)-1-((tetrahydrofuran-2-yl)methyl)-1*H*-pyrrole-3-carboxamide (**28**)

The Boc-protected amine (**22b**) was dissolved in HFIP in a microwave vial. The vial was capped and submitted to a microwave reactor (80 min, 150 °C). The solvent was removed under reduced pressure, and the crude amine intermediate was subjected to NBD coupling without further purification. The amine intermediate (8.2 mg, 12.4 μmol, 1.0 equiv) was dissolved in anhydrous DMF (1 mL) and anhydrous DIPEA (6.3 μL, 4.8 mg, 37.1 μmol, 3.0 equiv). To the solution, NBD-F (4.5 mg, 23.7 μmol, 2.0 equiv) was added. The reaction was stirred for 18 h at room temperature protected from light. The solvent was evaporated under reduced pressure, and the crude mixture was taken up in ACN/H_2_O (v/v 1/1), filtered, and purified by reversed-phase preparative HPLC (50–95% ACN/H_2_O). Fractions containing the product were combined and lyophilized to yield a yellow powder (5.6 mg, 7.0 μmol, 57%). ^1^H NMR (750 MHz, MeOD) δ (ppm): 8.50 (d, ^3^*J* = 8.8 Hz, 1H), 8.07–7.75 (m, 3H), 6.45–6.39 (m, 2H), 4.16–3.37 (m, 17H), 2.58 (s, 3H), 2.41–2.34 (m, 2H), 2.06–1.61 (m, 5H), 1.36 (s, 1H), 0.79 (t, ^3^*J* = 6.4 Hz, 6H). ^13^C NMR (189 MHz, MeOD) δ 175.87, 167.04, 146.74, 145.90, 145.53, 138.36, 135.28, 135.12, 134.24, 134.03, 133.62, 132.28, 128.64, 128.13, 126.93, 126.79, 125.52, 125.34, 124.07, 123.89, 123.39, 122.43, 116.70, 110.67, 110.19, 100.06, 79.02, 71.64, 71.32, 70.67, 69.84, 68.60, 65.98, 50.12, 40.55, 29.98, 28.81, 28.77, 26.38, 11.94, 8.45. HR-MS (ESI+) *m*/*z*: [M + H]^+^ calcd for C_37_H_43_F_6_N_7_O_8_, 828.3150; found, 828.3155.

#### (*R*)-*N*-(9-(4-((1-(5-(2,5-Bis(trifluoromethyl)phenyl)-2-methyl-1-((tetrahydrofuran-2-yl)methyl)-1*H*-pyrrol-3-yl)-3,3-diethyl-1,4-dioxo-8,11-dioxa-2,5-diazatridecan-13-yl)carbamoyl)-2-carboxyphenyl)-6-(dimethylamino)-3*H*-xanthen-3-ylidene)-*N*-methylmethanaminium 2,2,2-trifluoroacetate and (*R*)-*N*-(9-(5-((1-(5-(2,5-Bis(trifluoromethyl)phenyl)-2-methyl-1-((tetrahydrofuran-2-yl)methyl)-1*H*-pyrrol-3-yl)-3,3-diethyl-1,4-dioxo-8,11-dioxa-2,5-diazatridecan-13-yl)carbamoyl)-2-carboxyphenyl)-6-(dimethylamino)-3*H*-xanthen-3-ylidene)-*N*-methylmethanaminium 2,2,2-trifluoroacetate (**29**)

In a microwave vial, (**22b**) (25.0 mg, 32.7 μmol) was dissolved in HFIP (15 mL). The vial was capped and placed in a microwave reactor (150 °C, 90 min). The solvent was removed under reduced pressure. The crude amine was used for the next step without further purification (95% purity). The amine intermediate (7.10 mg, 9.63 μmol, 1.0 equiv) was dissolved together with 5/6-TAMRA-NHS (9.70 mg, 18.4 μmol, 1.9 equiv) in anhydrous DMF (0.5 mL) before anhydrous DIPEA (7.40 μL, 5.80 mg, 45.1 μmol, 4.7 equiv) was added. The reaction was stirred for 15 h covered from light. The solvent was removed under reduced pressure and the crude was taken up in ACN/H_2_O (v/v, 1/1), filtered, and purified by reversed-phase preparative HPLC (50–95% ACN + 0.1% TFA/H_2_O + 0.1% TFA). The title compound was obtained as a dark-violet powder after lyophilization (12.9 mg, 10.8 μmol, 89%). Mixture of 5- and 6-isomer. ^1^H NMR (750 MHz cryo, MeOD) δ (ppm): 8.79 (d, ^4^*J* = 1.8 Hz, 1H), 8.39 (d, ^3^*J* = 8.3 Hz, 1H), 8.26 (dd, ^3^*J* = 7.8, ^4^*J* = 1.9 Hz, 1H), 8.20 (dd, ^3^*J* = 8.3, ^4^*J* = 1.8 Hz, 1H), 8.10–7.76 (m, 7H), 7.50 (d, ^3^*J* = 7.8 Hz, 1H), 7.17–7.12 (m, 4H), 7.08–7.03 (m, 4H), 6.99–6.96 (m, 4H), 6.47–6.36 (m, 2H), 4.12–3.38 (m, 34H), 3.30–3.29 (m, 24H), 2.57 (s, 6H), 2.41–2.31 (m, 4H), 2.01–1.63 (m, 10H), 1.38–1.33 (m, 2H), 0.78 (t, ^3^*J* = 7.4 Hz, 6H), 0.75 (t, ^3^*J* = 7.4 Hz, 6H). ^13^C NMR (189 MHz cryo, MeOD) δ (ppm): 175.82, 175.78, 168.26, 167.96, 167.33, 166.98, 166.95, 162.74, 162.56, 162.37, 162.18, 160.69, 160.65, 159.09, 159.06, 158.99, 139.35, 138.14, 137.68, 135.54, 135.44, 135.29, 135.13, 135.01, 134.97, 134.83, 134.21, 134.01, 133.59, 132.84, 132.38, 132.09, 131.98, 131.93, 131.39, 130.28, 130.11, 128.63, 128.20, 126.96, 126.79, 125.53, 125.34, 124.08, 123.89, 122.44, 116.71, 115.60, 115.58, 114.89, 114.73, 110.67, 110.20, 97.46, 97.45, 78.98, 71.46, 71.32, 71.31, 71.27, 70.69, 70.61, 70.45, 70.36, 68.60, 65.96, 65.94, 50.15, 41.14, 41.08, 40.93, 40.63, 40.55, 29.98, 28.80, 28.77, 26.38, 12.06, 8.47. HR-MS (ESI+) *m*/*z*: [M + H]^+^ calcd for C_56_H_62_F_6_N_6_O_9_, 1077.4555; found, 1077.4561.

#### 5-(4-Chlorophenyl)-1-(2,4-dichlorophenyl)-4-methyl-*N*-(3-((2-(2-(2-((7-nitrobenzo[*c*][1,2,5]oxadiazol-4-yl)amino)ethoxy)ethoxy)ethyl)carbamoyl)pentan-3-yl)-1*H*-pyrazole-3-carboxamide (**30**)

The Boc-protected amine (**23c**) was dissolved in DCM (0.9 mL), and TFA (0.1 mL) was added. The solution was stirred for 2 h, and toluene (1 mL) was added. Then, the solvent mixture was removed under reduced pressure and subsequently coevaporated with toluene (×2). A crude product was subjected to NBD coupling without further purification. The TFA-ammonium intermediate (16.0 mg, 21.7 μmol, 1.0 equiv) was dissolved in anhydrous DMF (1 mL) and anhydrous DIPEA (14.0 μL, 11.0 mg, 86.8 μmol, 4.0 equiv). To the solution, NBD-F (6.0 mg, 32.6 μmol, 1.5 equiv) was added. The reaction was stirred for 19 h at room temperature protected from light. The solvent was evaporated under reduced pressure, and the crude mixture was taken up in ACN/H_2_O (v/v 1/1), filtered, and purified by reversed-phase preparative HPLC (50–95% ACN + 0.1% TFA/H_2_O + 0.1% TFA). Fractions containing the product were combined and lyophilized to yield an orange powder (10.2 mg, 12.9 μmol, 60%). ^1^H NMR (600 MHz, MeOD) δ (ppm): 8.49 (d, ^3^*J* = 8.7 Hz, 1H), 7.52–7.48 (m, 2H), 7.39 (dd, ^3^*J* = 8.5, ^4^*J* = 2.1 Hz, 1H), 7.38–7.32 (m, 2H), 7.21–7.16 (m, 2H), 6.39 (dd, ^3^*J* = 8.9, 2.1 Hz, 1H), 3.78 (t, ^3^*J* = 5.1 Hz, 2H), 3.71 (s, 2H), 3.66–3.62 (m, 2H), 3.62–3.59 (m, 2H), 3.55 (t, ^3^*J* = 5.6 Hz, 2H), 3.42 (t, ^3^*J* = 5.6 Hz, 2H), 2.50 (dq, ^2^*J* = 14.8, ^3^*J* = 7.4 Hz, 2H), 1.83 (dq, ^2^*J* = 14.5, ^3^*J* = 7.3 Hz, 2H), 0.81 (t, ^3^*J* = 7.4 Hz, 6H). ^13^C NMR (151 MHz, CDCl_3_) δ (ppm): 175.11, 163.36, 146.31, 145.84, 145.50, 144.76, 138.40, 137.33, 137.26, 136.17, 134.10, 132.50, 132.47, 130.99, 129.85, 129.16, 128.67, 123.32, 118.24, 100.21, 71.68, 71.30, 70.65, 69.88, 66.44, 44.81, 40.58, 29.22, 9.59, 8.53. HR-MS (ESI+) *m*/*z*: [M + H]^+^ calcd for C_35_H_37_Cl_3_N_8_O_7_, 789.1895; found, 789.1936.

#### *N*-(9-(2-Carboxy-4-((1-(5-(4-chlorophenyl)-1-(2,4-dichlorophenyl)-4-methyl-1*H*-pyrazol-3-yl)-3,3-diethyl-1,4-dioxo-8,11-dioxa-2,5-diazatridecan-13-yl)carbamoyl)phenyl)-6-(dimethylamino)-3*H*-xanthen-3-ylidene)-*N*-methylmethanaminium 2,2,2-trifluoroacetate and *N*-(9-(2-Carboxy-5-((1-(5-(4-chlorophenyl)-1-(2,4-dichlorophenyl)-4-methyl-1*H*-pyrazol-3-yl)-3,3-diethyl-1,4-dioxo-8,11-dioxa-2,5-diazatridecan-13-yl)carbamoyl)phenyl)-6-(dimethylamino)-3*H*-xanthen-3-ylidene)-*N*-methylmethanaminium 2,2,2-trifluoroacetate (**31**)

The Boc-protected amine (**23b**) was dissolved in DCM (0.9 mL), and TFA (0.1 mL) was added. The reaction was stirred for 2 h. Toluene (ca. 1 mL) was added, and the solvent was removed under reduced pressure. This procedure was repeated two more times to remove residual TFA. The crude material was used for the fluorescent dye coupling without further purification. The TFA-ammonium intermediate (16.0 mg, 21.6 μmol, 1.0 equiv) was dissolved together with 5/6-TAMRA-COOH (14.0 mg, 32.4 μmol, 1.5 equiv), EDCI (6.60 mg, 34.6 μmol, 1.6 equiv), and HOAt (4.70 mg, 34.6 μmol, 1.6 equiv) in anhydrous DMF (0.5 mL) before anhydrous DIPEA (15.0 μL, 11.1 mg, 86.4 μmol, 4.0 equiv) was added. The reaction was stirred for 20 h covered from light. The solvent was removed under reduced pressure, and the crude was taken up in ACN/H_2_O (v/v, 1/1), filtered, and purified by reversed-phase preparative HPLC (50–95% ACN + 0.1%TFA/H_2_O + 0.1%TFA). The title compound was obtained as a dark-violet powder after lyophilization (11.0 mg, 10.6 μmol, 49%). Mixture of 5- and 6-isomer. ^1^H NMR (600 MHz, MeOD) δ (ppm): 8.78 (d, ^4^*J* = 1.8 Hz, 1H), 8.40–8.37 (m, 1H), 8.27 (dd, ^3^*J* = 7.9, ^4^*J* = 1.9 Hz, 1H), 8.21 (dd, ^3^*J* = 8.2, ^4^*J* = 1.8 Hz, 1H), 7.85–7.82 (m, 1H), 7.53–7.48 (m, 3H), 7.45 (d, ^3^*J* = 8.5 Hz, 2H), 7.40–7.32 (m, 6H), 7.20–7.10 (m, 8H), 7.08–7.02 (m, 4H), 6.98–6.93 (m, 4H), 3.72 (t, ^3^*J* = 5.3 Hz, 2H), 3.69–3.61 (m, 7H), 3.61–3.54 (m, 10H), 3.49 (t, ^3^*J* = 5.6 Hz, 2H), 3.42 (t, ^3^*J* = 5.5 Hz, 2H), 3.30–3.25 (m, 24H), 2.58–2.44 (m, 4H), 2.29–2.26 (m, 6H), 1.89–1.75 (m, 4H), 0.79 (t, ^3^*J* = 7.4 Hz, 6H), 0.75 (t, ^3^*J* = 7.4 Hz, 6H). ^13^C NMR (151 MHz, MeOD) δ (ppm): 175.00, 174.99, 168.23, 167.93, 167.34, 167.33, 163.28, 163.25, 160.67, 160.62, 159.04, 158.95, 146.33, 146.29, 144.78, 144.75, 139.36, 138.12, 137.68, 137.30, 137.29, 137.26, 137.24, 136.19, 135.55, 134.76, 134.03, 132.83, 132.50, 132.48, 132.45, 132.44, 132.38, 132.06, 132.00, 131.95, 131.38, 131.02, 130.26, 130.16, 129.88, 129.22, 129.20, 128.64, 118.22, 115.65, 114.89, 114.74, 97.45, 71.53, 71.32, 71.29, 71.23, 70.69, 70.52, 70.43, 70.33, 66.46, 66.41, 41.17, 41.12, 40.96, 40.94, 40.69, 40.55, 29.24, 9.61, 8.59, 8.58. HR-MS (ESI+) *m*/*z*: [M + H]^+^ calcd for, C_54_H_56_Cl_3_N_7_O_8_; found, 1036.3320.

#### 8-Chloro-1-(2,4-dichlorophenyl)-*N*-(3-((2-(2-(2-((7-nitrobenzo[*c*][1,2,5]oxadiazol-4-yl)amino)ethoxy)ethoxy)ethyl)carbamoyl)pentan-3-yl)-1,4,5,6-tetrahydrobenzo[6,7]cyclohepta[1,2-*c*]pyrazole-3-carboxamide (**32**)

The Boc-protected amine (**24**) was dissolved in DCM (0.9 mL), and TFA (0.1 mL) was added. The solution was stirred for 2 h, and toluene (1 mL) was added. Then the solvent mixture was removed under reduced pressure and subsequently coevaporated with toluene (×2). A crude product was subjected to NBD coupling without further purification. The TFA-ammonium intermediate (9.89 mg, 15.2 μmol, 1.0 equiv) was dissolved in anhydrous DMF (1 mL) and anhydrous DIPEA (8.00 μL, 5.90 mg, 45.6 μmol, 3.0 equiv). To the solution, NBD-F (5.60 mg, 30.4 μmol, 2.0 equiv) was added. The reaction was stirred for 21 h at room temperature protected from light. The solvent was evaporated under reduced pressure, and the crude mixture was taken up in ACN/H_2_O (v/v 1/1), filtered, and purified by reversed-phase preparative HPLC (50–95% ACN + 0.1% TFA/H_2_O + 0.1% TFA). Fractions containing the product were combined and lyophilized to yield an orange powder (6.10 mg, 7.43 μmol, 49%). ^1^H NMR (750 MHz, MeOD) δ (ppm): 8.50 (d, ^3^*J* = 8.8 Hz, 1H), 7.66 (d, ^3^*J* = 8.4 Hz, 1H), 7.54 (d, ^4^*J* = 2.3 Hz, 1H), 7.51 (dd, ^3^*J* = 8.5, ^4^*J* = 2.3 Hz, 1H), 7.38 (d, ^4^*J* = 2.2 Hz, 1H), 7.06 (dd, *J* = 8.3, 2.2 Hz, 1H), 6.69 (d, ^3^*J* = 8.3 Hz, 1H), 6.40 (d, ^3^*J* = 8.9 Hz, 1H), 3.84–3.58 (m, 6H), 3.56 (t, ^3^*J* = 5.6 Hz, 2H), 3.42 (t, ^3^*J* = 5.6 Hz, 2H), 2.69 (t, ^3^*J* = 6.6 Hz, 2H), 2.49 (dq, ^2^*J* = 14.8, ^3^*J* = 7.4 Hz, 2H), 2.25 (t, ^3^*J* = 7.6 Hz, 2H), 1.84 (dq, ^2^*J* = 14.5, ^3^*J* = 7.3 Hz, 2H), 0.81 (t, ^3^*J* = 7.4 Hz, 6H). One bridging CH_2_-group signal was not resolved (Compare **24**). ^13^C NMR (151 MHz, MeOD) δ (ppm): 175.10, 163.33, 146.73, 145.85, 145.51, 145.41, 145.21, 143.94, 138.40, 137.32, 137.25, 135.49, 133.57, 132.32, 131.21, 130.88, 129.57, 129.56, 129.12, 127.27, 123.34, 122.86, 100.14, 71.68, 71.30, 70.66, 69.88, 66.46, 44.81, 40.58, 33.21, 32.72, 29.18, 21.19, 8.54. HR-MS (ESI+) *m*/*z*: [M + H]^+^ calcd for C_37_H_39_Cl_3_N_8_O_7_, 813.2080; found, 813.2077.

#### *N*-(9-(2-Carboxy-4-((1-(8-chloro-1-(2,4-dichlorophenyl)-1,4,5,6-tetrahydrobenzo[6,7]cyclohepta[1,2-*c*]pyrazol-3-yl)-3,3-diethyl-1,4-dioxo-8,11-dioxa-2,5-diazatridecan-13-yl)carbamoyl)phenyl)-6-(dimethylamino)-3*H*-xanthen-3-ylidene)-*N*-methylmethanaminium 2,2,2-trifluoroacetate and *N*-(9-(2-Carboxy-5-((1-(8-chloro-1-(2,4-dichlorophenyl)-1,4,5,6-tetrahydrobenzo[6,7]cyclohepta[1,2-*c*]pyrazol-3-yl)-3,3-diethyl-1,4-dioxo-8,11-dioxa-2,5-diazatridecan-13-yl)carbamoyl)phenyl)-6-(dimethylamino)-3*H*-xanthen-3-ylidene)-*N*-methylmethanaminium 2,2,2-trifluoroacetate (**33**)

The Boc-protected amine (**24**) was dissolved in DCM (0.9 mL) and TFA (0.1 mL) was added. The reaction was stirred for 2 h. Toluene (ca. 1 mL) was added, and the solvent was removed under reduced pressure. This procedure was repeated two more times to remove residual TFA. The crude material was used for the fluorescent dye coupling without further purification. TFA-ammonium intermediate (12.4 mg, 16.3 μmol, 1.0 equiv) was dissolved together with 5/6-TAMRA-NHS (9.40 mg, 17.9 μmol, 1.1 equiv) in anhydrous DMF (0.2 mL) before anhydrous DIPEA (14.0 μL, 10.5 mg, 81.4 μmol, 5.0 equiv) was added. The reaction was stirred for 16 h covered from light. The solvent was removed under reduced pressure, and the crude was taken up in ACN/H_2_O (v/v, 1/1), filtered, and purified by reversed-phase preparative HPLC (50–95% ACN + 0.1%TFA/H_2_O + 0.1%TFA). The title compound was obtained as a dark-violet powder after lyophilization (5.02 mg, 4.72 μmol, 38%). Mixture of 5- and 6-isomer. ^1^H NMR (600 MHz, MeOD) δ (ppm): 8.78 (s, 1H), 8.40 (d, ^3^*J* = 8.3 Hz, 1H), 8.27 (d, ^3^*J* = 7.9 Hz, 1H), 8.21 (d, ^3^*J* = 8.5 Hz, 1H), 7.83 (s, 1H), 7.60 (dd, ^3^*J* = 8.5, ^4^*J* = 3.5 Hz, 2H), 7.55 (d, ^3^*J* = 2.2 Hz, 2H), 7.52–7.46 (m, 3H), 7.37 (s, 2H), 7.17–7.10 (m, 4H), 7.07–7.02 (m, 6H), 6.95 (s, 4H), 6.67 (t, ^3^*J* = 8.7 Hz, 2H), 3.72 (t, ^3^*J* = 5.4 Hz, 2H), 3.67 (s, 8H), 3.61–3.53 (m, 8H), 3.50 (t, ^3^*J* = 5.6 Hz, 2H), 3.43 (t, ^3^*J* = 5.4 Hz, 2H), 3.35–3.31 (m, 2H), 3.28 (s, 24H), 2.70–2.65 (m, 4H), 2.55–2.44 (m, 4H), 2.22 (s, 4H), 1.89–1.77 (m, 4H), 0.80 (t, ^3^*J* = 7.4 Hz, 6H), 0.76 (t, ^3^*J* = 7.4 Hz, 6H). ^13^C NMR (151 MHz, MeOD) δ (ppm): 175.00, 174.98, 168.26, 167.96, 167.38, 163.24, 160.61, 160.57, 159.04, 159.02, 158.93, 145.43, 145.39, 145.19, 143.96, 139.33, 138.12, 137.66, 137.26, 137.23, 135.54, 135.51, 134.79, 133.54, 133.47, 132.85, 132.83, 132.38, 132.31, 132.29, 132.06, 131.98, 131.96, 131.40, 131.21, 130.90, 130.27, 130.15, 129.65, 129.62, 129.54, 129.07, 127.28, 122.84, 115.65, 114.88, 114.72, 97.47, 71.51, 71.31, 71.28, 71.21, 70.68, 70.53, 70.42, 70.31, 66.48, 66.45, 41.17, 41.10, 40.97, 40.95, 40.68, 40.55, 33.21, 32.69, 29.21, 21.19, 8.61, 8.59. HR-MS (ESI+) *m*/*z*: [M + H]^+^ calcd for C_56_H_58_Cl_3_N_7_O_8_, 1062.3485; found, 1062.3491.

#### 6-(4-Chlorophenyl)-5-(2-methoxyethoxy)-*N*-(3-((2-(2-(2-((7-nitrobenzo[*c*][1,2,5]oxadiazol-4-yl)amino)ethoxy)ethoxy)ethyl)carbamoyl)pentan-3-yl)pyrazine-2-carboxamide (**34**)

The Boc-protected amine (**26**) was dissolved in DCM (0.9 mL), and TFA (0.1 mL) was added. The solution was stirred for 2 h, and toluene (1 mL) was added. Then the solvent mixture was removed under reduced pressure and subsequently coevaporated with toluene (×2). The crude product was subjected to NBD coupling without further purification. The TFA-ammonium intermediate (7.83 mg, 11.8 μmol, 1.0 equiv) was dissolved in anhydrous DMF (1 mL) and anhydrous DIPEA (6.0 μL, 4.5 mg, 35.3 μmol, 3.0 equiv). To the solution, NBD-F (4.30 mg, 23.5 μmol, 2.0 equiv) was added. The reaction was stirred for 21 h at room temperature protected from light. The solvent was evaporated under reduced pressure, and the crude mixture was taken up in ACN/H_2_O (v/v 1/1), filtered, and purified by reversed-phase preparative HPLC (40–95% ACN + 0.1% TFA/H_2_O + 0.1% TFA). Fractions containing the product were combined and lyophilized to yield an orange-brown powder (3.75 mg, 5.24 μmol, 45%). ^1^H NMR (750 MHz, MeOD) δ (ppm): 8.72 (s, 1H), 8.40 (d, ^3^*J* = 8.8 Hz, 1H), 8.26–8.17 (m, 2H), 7.46–7.42 (m, 2H), 6.33 (d, ^3^*J* = 8.8 Hz, 1H), 4.68–4.64 (m, 2H), 3.85–3.81 (m, 2H), 3.78 (t, ^3^*J* = 5.4 Hz, 2H), 3.74–3.62 (m, 6H), 3.60 (t, ^3^*J* = 5.7 Hz, 2H), 3.49 (t, ^3^*J* = 5.7 Hz, 2H), 3.43 (s, 3H), 2.60 (dq, ^2^*J* = 14.9, ^3^*J* = 7.6 Hz, 2H), 1.84 (dq, ^2^*J* = 14.6, ^3^*J* = 7.3 Hz, 2H), 0.79 (t, ^3^*J* = 7.4 Hz, 6H). ^13^C NMR (189 MHz, MeOD) δ (ppm): 175.07, 163.70, 160.46, 146.67, 145.77, 145.40, 140.71, 140.23, 138.64, 138.32, 136.95, 134.54, 131.85, 129.45, 123.29, 100.00, 71.75, 71.53, 71.31, 70.72, 69.75, 67.71, 66.72, 59.24, 44.72, 40.68, 29.41, 8.61. HR-MS (ESI+) *m*/*z*: [M + H]^+^ calcd for C_32_H_39_ClN_8_O_9_, 715.2601; found, 715.2594.

#### *N*-(9-(2-Carboxy-4-((1-(6-(4-chlorophenyl)-5-(2-methoxyethoxy)pyrazin-2-yl)-3,3-diethyl-1,4-dioxo-8,11-dioxa-2,5-diazatridecan-13-yl)carbamoyl)phenyl)-6-(dimethylamino)-3*H*-xanthen-3-ylidene)-*N*-methylmethanaminium 2,2,2-trifluoroacetate and *N*-(9-(2-Carboxy-5-((1-(6-(4-chlorophenyl)-5-(2-methoxyethoxy)pyrazin-2-yl)-3,3-diethyl-1,4-dioxo-8,11-dioxa-2,5-diazatridecan-13-yl)carbamoyl)phenyl)-6-(dimethylamino)-3*H*-xanthen-3-ylidene)-*N*-methylmethanaminium 2,2,2-trifluoroacetate (**35**)

The Boc-protected amine (**26**) was dissolved in DCM (0.9 mL), and TFA (0.1 mL) was added. The reaction was stirred for 2 h. Toluene (ca. 1 mL) was added, and the solvent was removed under reduced pressure. This procedure was repeated two more times to remove residual TFA. The crude material was used for the fluorescent dye coupling without further purification. TFA-ammonium intermediate (14.0 mg, 21.0 μmol, 1.0 equiv) was dissolved together with 5/6-TAMRA-COOH (13.6 mg, 31.5 μmol, 1.5 equiv), EDCI (6.50 mg, 33.7 μmol, 1.6 equiv), and HOAT (4.60 mg, 33.7 μmol, 1.6 equiv) in anhydrous DMF (0.5 mL) before anhydrous DIPEA (18.0 μL, 13.6 mg, 81.4 μmol, 5.0 equiv) was added. The reaction was stirred for 17 h covered from light. The solvent was removed under reduced pressure, and the crude was taken up in ACN/H_2_O (v/v, 1/1), filtered, and purified by reversed-phase preparative HPLC (50–95% ACN + 0.1% TFA/H_2_O + 0.1% TFA). The title compound was obtained as a dark-violet powder after lyophilization (7.62 mg, 7.90 μmol, 38%). Mixture of 5- and 6-isomer. ^1^H NMR (600 MHz cryo, MeOD) δ (ppm): 8.75 (d, ^4^*J* = 1.8 Hz, 1H), 8.71 (s, 1H), 8.63 (s, 1H), 8.38 (d, ^3^*J* = 8.3 Hz, 1H), 8.27 (dd, ^3^*J* = 7.9, ^4^*J* = 1.8 Hz, 1H), 8.23–8.18 (m, 5H), 7.83 (d, ^4^*J* = 1.8 Hz, 1H), 7.53–7.48 (m, 1H), 7.48–7.42 (m, 2H), 7.39–7.33 (m, 2H), 7.12 (d, ^3^*J* = 9.5 Hz, 2H), 7.06–7.02 (m, 4H), 6.98–6.95 (m, 1H), 6.93 (d, ^4^*J* = 2.4 Hz, 2H), 6.84 (d, ^4^*J* = 2.5 Hz, 2H), 4.75–4.70 (m, 2H), 4.70–4.65 (m, 2H), 3.91–3.85 (m, 2H), 3.85–3.81 (m, 2H), 3.75–3.71 (m, 2H), 3.69 (s, 4H), 3.68–3.64 (m, 6H), 3.64–3.57 (m, 6H), 3.54 (t, ^3^*J* = 5.6 Hz, 2H), 3.50 (t, ^3^*J* = 5.7 Hz, 2H), 3.47 (s, 2H), 3.43 (s, 3H), 3.38 (t, ^3^*J* = 5.6 Hz, 2H), 3.28–3.26 (m, 24H), 2.63–2.52 (m, 4H), 1.87–1.77 (m, 4H), 0.77–0.70 (m, 12H). ^13^C NMR (151 MHz cryo, MeOD) δ (ppm): 174.95, 174.93, 168.29, 167.99, 167.36, 167.32, 163.62, 163.47, 160.58, 160.51, 160.44, 160.36, 159.05, 158.93, 158.84, 158.83, 158.80, 158.79, 140.92, 140.36, 140.28, 140.13, 139.36, 138.74, 138.60, 137.94, 137.73, 136.96, 136.91, 135.50, 134.67, 134.36, 132.80, 132.27, 132.06, 131.94, 131.91, 131.83, 131.70, 131.67, 131.49, 130.33, 130.05, 129.53, 129.50, 115.59, 115.52, 114.88, 114.62, 97.48, 97.46, 97.43, 71.64, 71.59, 71.56, 71.45, 71.42, 71.31, 70.65, 70.57, 70.46, 70.32, 67.82, 67.74, 66.77, 66.68, 59.29, 59.23, 49.57, 49.43, 49.28, 49.14, 49.00, 48.86, 48.72, 48.58, 41.22, 41.16, 40.92, 40.73, 29.45, 29.35, 8.72, 8.62. HR-MS (ESI+) *m*/*z*: [M + H]^+^ calcd for C_51_H_58_ClN_7_O_10_, 964.4006; found, 964.4013.

#### 1-(3-Fluorobenzyl)-*N*-(3-((2-(2-(2-((7-nitrobenzo[*c*][1,2,5]oxadiazol-4-yl)amino)ethoxy)ethoxy)ethyl)carbamoyl)pentan-3-yl)-1*H*-indazole-3-carboxamide (**36**)

The Boc-protected amine (**27**) was dissolved in DCM (0.9 mL), and TFA (0.1 mL) was added. The solution was stirred for 2 h, and toluene (1 mL) was added. Then the solvent mixture was removed under reduced pressure and subsequently coevaporated with toluene (×2). The crude product was subjected to NBD coupling without further purification. The TFA-ammonium intermediate (10.2 mg, 16.2 μmol, 1.0 equiv) was dissolved in anhydrous DMF (1 mL) and anhydrous DIPEA (8.2 μL, 6.3 mg, 48.5 μmol, 3.0 equiv). To the solution, NBD-F (5.9 mg, 32.3 μmol, 2.0 equiv) was added. The reaction was stirred for 21 h at room temperature protected from light. The solvent was evaporated under reduced pressure, and the crude mixture was taken up in ACN/H_2_O (v/v 1/1), filtered, and purified by reversed-phase preparative HPLC (40–95% ACN + 0.1% TFA/H_2_O + 0.1% TFA). Fractions containing the product were combined and lyophilized to yield an orange powder (3.06 mg, 4.52 μmol, 28%). ^1^H NMR (600 MHz, MeOD) δ (ppm): 8.49 (d, ^3^*J* = 8.8 Hz, 1H), 8.22 (d, ^3^*J* = 8.2 Hz, 1H), 7.57 (d, ^3^*J* = 8.5 Hz, 1H), 7.47–7.37 (m, 1H), 7.34–7.25 (m, 2H), 7.05 (d, ^3^*J* = 7.7 Hz, 1H), 7.01–6.93 (m, 2H), 6.38 (d, ^3^*J* = 8.8 Hz, 1H), 2.54 (dq, ^2^*J* = 14.8, ^3^*J* = 7.5 Hz, 2H), 1.90 (dq, ^2^*J* = 14.6, ^3^*J* = 7.4 Hz, 2H), 0.84 (t, ^3^*J* = 7.4 Hz, 6H). ^13^C NMR (151 MHz, MeOD) δ (ppm): 175.23, 165.00, 163.70, 163.22, 146.72, 145.87, 145.50, 142.49, 140.68, 140.65, 139.07, 138.36, 131.67, 131.63, 128.27, 124.14, 124.12, 124.06, 123.98, 123.30, 123.17, 115.74, 115.63, 115.16, 115.04, 111.16, 100.05, 71.63, 71.31, 70.68, 69.82, 66.43, 53.50, 44.74, 40.62, 29.23, 8.53. HR-MS (ESI+) *m*/*z*: [M + Na]^+^ calcd for C_32_H_44_FN_5_O_6_, 699.2661; found, 699.2654.

#### *N*-(9-(2-Carboxy-4-((3,3-diethyl-1-(1-(3-fluorobenzyl)-1*H*-indazol-3-yl)-1,4-dioxo-8,11-dioxa-2,5-diazatridecan-13-yl)carbamoyl)phenyl)-6-(dimethylamino)-3*H*-xanthen-3-ylidene)-*N*-methylmethanaminium 2,2,2-trifluoroacetate and *N*-(9-(2-Carboxy-5-((3,3-diethyl-1-(1-(3-fluorobenzyl)-1*H*-indazol-3-yl)-1,4-dioxo-8,11-dioxa-2,5-diazatridecan-13-yl)carbamoyl)phenyl)-6-(dimethylamino)-3*H*-xanthen-3-ylidene)-*N*-methylmethanaminium 2,2,2-trifluoroacetate (**37**)

The Boc-protected amine (**27**) was dissolved in DCM (0.9 mL) and TFA (0.1 mL) was added. The reaction was stirred for 2 h. Toluene (ca. 1 mL) was added and the solvent was removed under reduced pressure. This procedure was repeated two more times to remove residual TFA. The TFA-ammonium intermediate (11.3 mg, 18.0 μmol, 1.0 equiv) was dissolved together with 5/6-TAMRA-COOH (11.6 mg, 27.0 μmol, 1.5 equiv), EDCI (5.5 mg, 28.8 μmol, 1.6 equiv), and HOAt (3.90 mg, 28.8 μmol, 1.6 equiv) in anhydrous DMF (0.5 mL) before anhydrous DIPEA (12.2 μL, 9.30 mg, 72.0 μmol, 4.0 equiv) was added. The reaction was stirred for 20 h covered from light. The solvent was removed under reduced pressure, and the crude was taken up in ACN/H_2_O (v/v, 1/1), filtered, and purified by reversed-phase preparative HPLC (50–95% ACN + 0.1%TFA/H_2_O + 0.1%TFA). The fractions were pooled based on purity determined via LC–MS. The title compound was obtained as a red-violet powder after lyophilization (2.72 mg, 2.94 μmol, 16%). Mixture of 5- and 6-isomer. ^1^H NMR (600 MHz, MeOD) δ (ppm): 8.80 (d, ^4^*J* = 1.8 Hz, 1H), 8.40 (d, ^3^*J* = 8.2 Hz, 1H), 8.28 (dd, ^3^*J* = 7.9, ^4^*J* = 1.9 Hz, 1H), 8.24–8.18 (m, 2H), 8.19–8.14 (m, 1H), 7.86 (d, ^3^*J* = 1.7 Hz, 1H), 7.60–7.54 (m, 2H), 7.51 (d, ^3^*J* = 7.9 Hz, 1H), 7.46–7.40 (m, 2H), 7.33–7.23 (m, 4H), 7.13 (d, *J* = 9.4 Hz, 4H), 7.05–6.92 (m, 14H), 5.70 (s, 2H), 5.67 (s, 2H), 3.71 (t, ^3^*J* = 5.2 Hz, 2H), 3.66 (s, 3H), 3.65–3.59 (m, 4H), 3.57 (d, ^3^*J* = 7.8 Hz, 9H), 3.52 (t, ^3^*J* = 5.5 Hz, 2H), 3.47 (t, ^3^*J* = 5.5 Hz, 2H), 3.35 (t, *J* = 5.5 Hz, 2H), 3.26 (d, ^3^*J* = 1.6 Hz, 24H), 2.57–2.46 (m, 4H), 1.92–1.80 (m, 4H), 0.80 (t, ^3^*J* = 7.4 Hz, 6H), 0.77 (t, ^3^*J* = 7.4 Hz, 6H). HR-MS (ESI+) *m*/*z*: [M + H]^+^ calcd for C_52_H_56_FN_7_O_8_, 926.4247; found, 926.4259.

#### (9*H*-Fluoren-9-yl)methyl (3-((2-(2-(2-((7-Nitrobenzo[*c*][1,2,5]oxadiazol-4-yl)amino)ethoxy)ethoxy)ethyl)carbamoyl)pentan-3-yl)carbamate (**38**)

The Fmoc-protected amine (**21b**) (77.2 mg, 132.3 μmol) was dissolved in DCM (4.5 mL) before TFA (0.5 mL) was added. The reaction was stirred for 2 h. Toluene (ca. 1 mL) was added, and the solvent was removed under reduced pressure. This procedure was repeated two more times to remove residual TFA. A portion of the crude material was used without further purification for the NBD-coupling. The Boc-deprotected TFA-ammonium intermediate (45.1 mg, 75.5 μmol. 1.0 equiv) was dissolved in DMF (1 mL) together with NBD-F (20.7 mg, 113 μmol, 1.3 equiv) and DIPEA (64.0 μL, 49.0 mg, 378 μmol, 5.0 equiv). The reaction was stirred for 17 h before the solvent was removed under reduced pressure. The residue was taken up in ACN/H_2_O (v/v, 1/1), filtered, and purified by reversed-phase preparative HPLC (50–95% ACN + 0.1% TFA/H_2_O + 0.1% TFA). The title compound was obtained as a yellow powder (19.1 mg, 29.5 μmol, 39%) after lyophilization. ^1^H NMR (600 MHz, MeOD) δ (ppm): 8.47 (d, ^3^*J* = 8.8 Hz, 1H), 7.75 (d, ^3^*J* = 7.5 Hz, 2H), 7.63 (d, ^3^*J* = 7.5 Hz, 2H), 7.35 (t, ^3^*J* = 7.4 Hz, 2H), 7.28 (t, ^3^*J* = 1.2 Hz, 2H), 6.34 (d, ^3^*J* = 8.8 Hz, 1H), 4.35 (s, 2H), 4.18 (t, ^3^*J* = 6.3 Hz, 1H), 3.74 (t, ^3^*J* = 5.2 Hz, 2H), 3.70–3.56 (m, 6H), 3.51 (s, 2H), 3.36 (s, 2H), 1.97 (s, 2H), 1.77 (s, 2H), 0.72 (s, 6H). ^13^C NMR (151 MHz, MeOD) δ (ppm):175.58, 156.34, 145.84, 145.51, 145.48, 145.29, 142.61, 138.32, 128.76, 128.12, 126.10, 123.41, 120.91, 99.92, 71.58, 71.26, 70.65, 69.78, 67.26, 64.87, 49.57, 40.42, 28.02, 8.11. HR-MS (ESI+) *m*/*z*: [M + H]^+^ calcd for C_33_H_38_N_6_O_8_, 647.2824; found, 647.2833.

#### 6-(Cyclopropylmethoxy)-5-(4-fluorophenyl)-*N*-(3-((2-(2-(2-((7-nitrobenzo[*c*][1,2,5]oxadiazol-4-yl)amino)ethoxy)ethoxy)ethyl)carbamoyl)pentan-3-yl)nicotinamide (**40**)

The Fmoc-protected amine (**38**) (12.0 mg, 18.6 μmol, 1.0 equiv) was dissolved in anhydrous DMF (1 mL) together with DBU (3.50 μL, 3.7 mg, 27.8 μmol, 1.5 equiv). After 30 min, HOAt (4.0 mg, 29.7 μmol, 1.6 equiv) was added to the mixture and stirred for another 10 min. In a separate flask (**45**) (6.4 mg, 22.3 μmol, 1.2 equiv) was activated with HATU (8.4 mg, 22.3 μmol, 1.2 equiv) and DIPEA (15.7 μL, 12.0 mg, 92.8 μmol, 5.0 equiv) in anhydrous DMF (1 mL) and stirred for 20 min. The activated acid was added to the amine component, and the reaction was followed via LC–MS for 3 h. The solvent was removed under reduced pressure, and the mixture was taken up in ACN/H_2_O (v/v 1/1), filtered, and purified by reversed-phase preparative HPLC (50–95% ACN/H_2_O). The title compound was obtained as a highly viscous oil (18.0 mg, 30.7 μmol, 60%) after lyophilization. The cyclopropylmethoxy moiety appeared to be acid labile. Any acidic conditions during the reaction should be avoided. ^1^H NMR (600 MHz, MeOD) δ (ppm): 8.56 (d, ^4^*J* = 2.4 Hz, 1H), 8.48 (d, ^3^*J* = 8.8 Hz, 1H), 8.07 (d, ^4^*J* = 2.4 Hz, 1H), 7.67–7.60 (m, 2H), 7.18–7.11 (m, 2H), 6.38 (d, ^3^*J* = 8.8 Hz, 1H), 4.25 (d, ^3^*J* = 7.1 Hz, 2H), 3.75 (t, ^3^*J* = 5.1 Hz, 2H), 3.69 (br s, 2H), 3.60 (s, 4H), 3.55 (t, ^3^*J* = 5.7 Hz, 2H), 3.41 (t, ^3^*J* = 5.7 Hz, 2H), 2.22 (dq, ^2^*J* = 14.7, ^3^*J* = 7.4 Hz, 2H), 1.96 (dq, ^2^*J* = 14.7, ^3^*J* = 7.4 Hz, 2H), 1.26 (s, 1H), 0.81 (t, ^3^*J* = 7.4 Hz, 6H), 0.60–0.53 (m, 2H), 0.36–0.30 (m, 2H). ^13^C NMR (151 MHz cryo, MeOD) δ (ppm): 175.53, 166.81, 163.91 (d, ^1^*J*_C–F_ = 246.2 Hz), 163.77, 146.79, 146.63, 145.86, 145.51, 138.57, 138.32, 133.39 (d, ^4^*J*_C–F_ = 3.4 Hz), 132.23 (d, ^3^*J*_C–F_ = 8.2 Hz), 125.49, 124.44, 123.39, 116.01 (d, ^2^*J*_C–F_ = 21.7 Hz).100.09, 72.56, 71.63, 71.29, 70.71, 69.81, 66.03, 44.76, 40.56, 27.84, 10.90, 8.29, 3.58. HR-MS (ESI+) *m*/*z*: [M + H]^+^ calcd for C_34_H_40_FN_7_O_8_, 694.2995; found, 694.3015.

#### *N*-(9-(2-Carboxy-4-((5,5-diethyl-1-(9*H*-fluoren-9-yl)-3,6-dioxo-2,10,13-trioxa-4,7-diazapentadecan-15-yl)carbamoyl)phenyl)-6-(dimethylamino)-3*H*-xanthen-3-ylidene)-*N*-methylmethanaminium 2,2,2-Trifluoroacetate and *N*-(9-(2-Carboxy-5-((5,5-diethyl-1-(9*H*-fluoren-9-yl)-3,6-dioxo-2,10,13-trioxa-4,7-diazapentadecan-15-yl)carbamoyl)phenyl)-6-(dimethylamino)-3*H*-xanthen-3-ylidene)-*N*-methylmethanaminium 2,2,2-Trifluoroacetate (**39**)

The Fmoc-protected (**21b**) (77.4 mg, 133 μmol) was dissolved in DCM (4.5 mL) before TFA (0.5 mL) was added. The reaction was stirred for 2 h. Toluene (ca. 1 mL) was added, and the solvent was removed under reduced pressure. This procedure was repeated two more times to remove residual TFA. A portion of the crude material was used without further purification for the TAMRA coupling. The Boc-deprotected TFA-ammonium intermediate (56.3 mg, 94.2 μmol. 1.0 equiv) was dissolved in anhydrous DMF (1 mL) together with 5(6)-TAMRA-COOH (66.7 mg, 155 μmol, 1.5 equiv), EDCI (31.7 mg, 165 μmol, 1.6 equiv), HOAt (22.3 mg, 165 μmol, 1.6 equiv), and anhydrous DIPEA (70.0 μL, 53.0 mg, 411 μmol, 4.0 equiv). The reaction was stirred for 22 h before the solvent was removed under reduced pressure. The residue was taken up in ACN/H_2_O (v/v, 1/1), filtered, and purified by reversed-phase preparative HPLC (50–95% ACN + 0.1% TFA/H_2_O + 0.1% TFA). The title compound was obtained as a red-violet powder (14.8 mg, 14.6 μmol, 16%) after lyophilization. Mixture of 5- and 6 isomer. ^1^H NMR (600 MHz, MeOD) δ 8.79 (d, ^4^*J* = 1.8 Hz, 1H), 8.40 (d, ^3^*J* = 8.2 Hz, 1H), 8.27 (dd, ^3^*J* = 7.9, ^4^*J* = 1.8 Hz, 1H), 8.21 (dd, ^3^*J* = 8.3, ^4^*J* = 1.8 Hz, 1H), 7.83 (d, ^4^*J* = 1.8 Hz, 1H), 7.76 (dd, ^3^*J* = 7.6, ^4^*J* = 2.3 Hz, 4H), 7.60 (d, ^3^*J* = 7.6 Hz, 1H), 7.57 (d, ^3^*J* = 7.5 Hz, 2H), 7.48 (d, ^3^*J* = 7.9 Hz, 1H), 7.35 (td, ^3^*J* = 7.5, ^4^*J* = 3.2 Hz, 4H), 7.26 (q, ^3^*J* = 6.8 Hz, 4H), 7.08 (d, ^3^*J* = 9.5 Hz, 4H), 6.98 (dt, ^3^*J* = 9.5, ^4^*J* = 2.7 Hz, 4H), 6.92–6.88 (m, 4H), 4.4–4.25 (m, 4H), 4.14–4.03 (m, 2H), 3.71 (t, ^3^*J* = 5.2 Hz, 2H), 3.69–3.36 (m, 22H), 3.26 (s, 24H), 2.05–1.91 (m, 2H), 1.77 (s, 2H), 0.72 (s, 12H). LC–MS (ESI+) *m*/*z*: [M + H]^+^ calcd for C_52_H_57_N_5_O_9_, 896.4229; found, 896.4.

#### *N*-(9-(2-Carboxy-4-((1-(6-(cyclopropylmethoxy)-5-(4-fluorophenyl)pyridin-3-yl)-3,3-diethyl-1,4-dioxo-8,11-dioxa-2,5-diazatridecan-13-yl)carbamoyl)phenyl)-6-(dimethylamino)-3*H*-xanthen-3-ylidene)-*N*-methylmethanaminium 2,2,2-Trifluoroacetate and *N*-(9-(2-Carboxy-5-((1-(6-(cyclopropylmethoxy)-5-(4-fluorophenyl)pyridin-3-yl)-3,3-diethyl-1,4-dioxo-8,11-dioxa-2,5-diazatridecan-13-yl)carbamoyl)phenyl)-6-(dimethylamino)-3*H*-xanthen-3-ylidene)-*N*-methylmethanaminium 2,2,2-Trifluoroacetate (**41**)

To a solution of (**39**) (13.0 mg, 14.5 μmol, 1.0 equiv) in DMF (1 mL) was added DBU (2.70 μL, 2.90 μg, 29.8 μmol, 1.5 equiv). After 30 min, HOAt (3.10 μg, 23.3 μmol, 1.6 equiv) was added to the mixture and stirred for another 10 min. (**45**) (5.00 mg, 17.4 μmol, 1.2 equiv) together with EDCI (3.4 mg, 17.4 μmol, 1.2 equiv) and DIPEA (12.4 μL, 9.40 mg, 72.5 μmol, 5.0 equiv) were added, and the reaction was followed via LC–MS. Upon uncompleted conversion, another portion of (**45**) (5.00 mg, 17.4 μmol, 1.2 equiv) together with EDCI (3.40 mg, 17.4 μmol, 1.2 equiv) and DIPEA (12.4 μL, 9.40 mg, 72.5 μmol) was added. After another 21 h, the solvent was evaporated, and the mixture taken up in ACN/H_2_O (v/v 1/1), filtered, and purified by reversed-phase preparative HPLC (50–95% ACN/H_2_O). The title compound was obtained as a violet powder (9.47 mg, 10.0 μmol, 69%) after lyophilization. Mixture of 5 and 6 isomer. ^1^H NMR (600 MHz, MeOD) δ (ppm): 8.77 (d, ^4^*J* = 1.8 Hz, 1H), 8.54 (d, ^4^*J* = 2.4 Hz, 1H), 8.53 (d, ^4^*J* = 2.4 Hz, 1H), 8.39 (d, ^3^*J* = 8.2 Hz, 1H), 8.25 (dd, ^3^*J* = 7.9, ^4^*J* = 1.8 Hz, 1H), 8.19 (dd, ^3^*J* = 8.3, ^4^*J* = 1.8 Hz, 1H), 8.05 (d, ^4^*J* = 2.4 Hz, 1H), 8.03 (d, ^4^*J* = 2.4 Hz, 1H), 7.81 (d, ^4^*J* = 1.8 Hz, 1H), 7.67–7.59 (m, 4H), 7.49 (d, ^3^*J* = 7.9 Hz, 1H), 7.18–7.09 (m, 8H), 7.05–6.99 (m, 4H), 6.97–6.91 (m, 4H), 4.29–4.22 (m, 4H), 3.69 (t, ^3^*J* = 5.4 Hz, 3H), 3.66–3.61 (m, 9H), 3.61–3.54 (m, 7H), 3.51 (t, ^3^*J* = 5.5 Hz, 2H), 3.43 (t, ^3^*J* = 5.6 Hz, 3H), 3.36–3.33 (m, 2H), 3.28 (s, 24H), 2.27–2.16 (m, 4H), 2.01–1.91 (m, 4H), 1.30–1.21 (m, 2H), 0.84–0.76 (m, 12H), 0.60–0.52 (m, 4H), 0.36–0.29 (m, 4H). ^13^C NMR (151 MHz, CDCl_3_) δ (ppm): 175.48, 168.20, 167.99, 167.33, 166.71, 164.72, 163.73, 163.09, 160.63, 160.59, 159.06, 159.01, 158.95, 146.64, 139.41, 138.56, 138.52, 138.09, 137.66, 135.50, 134.83, 133.37, 132.87, 132.82, 132.35, 132.26, 132.21, 132.05, 131.94, 131.38, 130.37, 130.03, 125.54, 124.35, 116.14, 116.13, 116.00, 115.97, 115.57, 114.88, 114.71, 97.47, 72.59, 71.43, 71.33, 71.29, 70.68, 70.45, 70.34, 66.06, 65.98, 41.12, 40.92, 40.64, 40.58, 27.88, 27.79, 10.93, 8.35, 8.32, 3.62. HR-MS (ESI+) *m*/*z*: [M + H]^+^ calcd. for C_53_H_59_FN_6_O_9_, 943.4400; found, 943.4405.

#### (*R*)-5-(2,5-Bis(trifluoromethyl)phenyl)-2-methyl-*N*-(3-((2-(2-(2-((7-nitrobenzo[*c*][1,2,5]thiadiazol-4-yl)amino)ethoxy)ethoxy)ethyl)carbamoyl)pentan-3-yl)-1-((tetrahydrofuran-2-yl)methyl)-1*H*-pyrrole-3-carboxamide (**51**)

The Boc-protected amine (**22b**) was dissolved in HFIP in a microwave vial. The vial was capped and submitted to a microwave reactor (80 min, 150 °C). The solvent was removed under reduced pressure, and the crude amine intermediate was subjected to fluorescent dye coupling without further purification. The amine intermediate (4.82 mg, 7.26 μmol, 1.0 equiv) was dissolved in MeOH (0.5 mL) and anhydrous DIPEA (4.90 μL, 3.70 mg, 29.0 μmol, 4.0 equiv) together with 4-fluoro-7-nitrobenzo[*c*][1,2,5]thiadiazole (2.89 mg, 14.5 μmol, 2.0 equiv). The reaction was stirred for 18 h at room temperature protected from light. The solvent was evaporated under reduced pressure, and the crude mixture was taken up in ACN/H_2_O (v/v 1/1), filtered, and purified by reversed-phase preparative HPLC (50–95% ACN/H_2_O). Fractions containing the product were combined and lyophilized to yield a bright yellow powder (1.28 mg, 1.52 μmol, 21%). ^1^H NMR (6000 MHz, MeOD) δ (ppm): 8.67–8.61 (m, 1H), 8.10–7.75 (m, 3H), 6.62–6.57 (m, 1H), 6.43 (s, 1H), 4.09–3.38 (m, 17H), 2.57 (s, 3H), 2.37 (dq, ^2^*J* = 17.6, ^3^*J* = 8.7 Hz, 2H), 2.04–1.58 (m, 5H), 1.39–1.31 (m, 1H), 0.78 (t, ^3^*J* = 7.4 Hz, 6H). HR-MS (ESI+) *m*/*z*: [M + H]^+^ calcd for C_37_H_43_F_6_N_7_O_7_S, 844.2922; found, 844.2959.

#### (*R*)-5-(2,5-Bis(trifluoromethyl)phenyl)-2-methyl-*N*-(3-((2-(2-(2-((7-nitro-2*H*-benzo[*d*][1,2,3]triazol-4-yl)amino)ethoxy)ethoxy)ethyl)carbamoyl)pentan-3-yl)-1-((tetrahydrofuran-2-yl)methyl)-1*H*-pyrrole-3-carboxamide (**52**)

The Boc-protected amine (**22b**) was dissolved in HFIP in a microwave vial. The vial was capped and submitted to a microwave reactor (80 min, 150 °C). The solvent was removed under reduced pressure, and the crude amine intermediate was subjected to fluorescent dye coupling without further purification. The amine intermediate (6.00 mg, 9.03 μmol, 1.0 equiv) was dissolved in DMF (1 mL) and anhydrous DIPEA (4.60 μL, 3.50 mg, 27.10 μmol, 3.0 equiv) together with 4-fluoro-7-nitro-2*H*-benzo[*d*][1,2,3]triazole (3.30 mg, 18.1 μmol, 2.0 equiv). The reaction was stirred for 18 h at room temperature protected from light. The solvent was evaporated under reduced pressure, and the crude mixture was taken up in ACN/H_2_O (v/v 1/1), filtered, and purified by reversed-phase preparative HPLC (50–95% ACN/H_2_O). Fractions containing the product were combined and lyophilized to yield a yellow powder (1.19 mg, 1.44 μmol, 16%). ^1^H NMR (6000 MHz, MeOD) δ (ppm): 8.21 (d, ^3^*J* = 9.0 Hz, 1H), 8.08–7.72 (m, 3H), 6.53 (d, ^3^*J* = 9.0 Hz, 1H), 6.41 (s, 1H), 4.13–3.51 (m, 14H), 3.43 (t, ^3^*J* = 5.5 Hz, 3H), 2.57 (s, 3H), 2.35 (dq, ^2^*J* = 14.8, ^3^*J* = 7.4 Hz, 2H), 2.08–1.53 (m, 5H), 1.37–1.25 (m, 1H), 0.78 (t, ^3^*J* = 7.4 Hz, 7H). HR-MS (ESI+) *m*/*z*: [M + H]^+^ calcd for C_37_H_44_F_6_N_8_O_7_, 827.3310; found, 827.3387.

## Supporting Information Available

Molecular formula strings (CSV)
Binding data (radioligand assay, CB_1_R and CB_2_R) and functional activity (cAMP, CB_1_R) expressed as p*K*_i_ ± SEM and pEC_50_ or pIC_50_ ± SEM values; antagonist activity (cAMP, CB_1_R); TR-FRET binding data (CB_1_R) expressed as p*K*_i_ ± SEM or p*K*_D_ ± SEM; live cell confocal imaging of **29** on CB_2_R-HEK293TR, HEK293TR and CB_1_R-HEK293TR cells (at 1 and 3 min time point); brightfield images and cell membrane staining images; superimposed docking structures of compounds **14**, **15**, **16**, **17**, **18**, **19,** and **22b**, **23b**, **24**, **25**, **26**, and **27** in the CB_1_R X-ray crystal structure (PDB ID: 5TGZ); superimposed docking structures of compound **14** with cocrystallized ligand AM6538 in CB_1_R X-ray crystal structure (PDB ID: 5TGZ); experimental description for conformational MD simulation and clustered representation of most frequent conformations of **29** as 5-isomer and 6-isomer in open and closed rhodamine equilibrium in water and *n*-octane respectively; mean 3D PSA and mean intramolecular hydrogen bonds values for each of the isomers of **29** in water and *n*-octane respectively; photophysical characterization of fluorescent probes in PBS; comparison of ^1^H NMR spectra of **24** at different temperatures; ^1^H NMR spectrum of side product **S53**; and analytical HPLC chromatograms of compounds **28**, **29**, **30**, **31**, **32**, **33**, **34**, **35**, **36**, **37**, **40,** and **41**. (PDF)
Representative video of CB_1_R staining on live CB_1_R expressing HEK293TR cells with **29** (250 nm, red, at 63× magnification) over 10 min (MP4)
Docking pose of CB1R ligand **14** in PDB 5TGZ (PDB)
Docking pose of CB1R ligand **15** in PDB 5TGZ, 2.80 Å (PDB)
Docking pose of CB1R ligand **16** in PDB 5TGZ, 2.80 Å (PDB)
Docking pose of CB1R ligand **17** in PDB 5TGZ, 2.80 Å (PDB)
Docking pose of CB1R ligand **18** in PDB 5TGZ, 2.80 Å (PDB)
Docking pose of CB1R ligand **19** in PDB 5TGZ, 2.80 Å (PDB)
Docking pose of CB1R ligand **22b** in PDB 5TGZ, 2.80 Å (PDB)
Docking pose of CB1R ligand **23b** in PDB 5TGZ, 2.80 Å (PDB)
Docking pose of CB1R ligand **24** in PDB 5TGZ, 2.80 Å (PDB)
Docking pose of CB1R ligand **25** in PDB 5TGZ, 2.80 Å (PDB)
Docking pose of CB1R ligand **26** in PDB 5TGZ, 2.80 Å (PDB)
Docking pose of CB1R ligand **27** in PDB 5TGZ, 2.80 Å(PDB)

## Supplementary Material

jm4c00465_si_003.mp4

jm4c00465_si_004.pdb

jm4c00465_si_005.pdb

jm4c00465_si_006.pdb

jm4c00465_si_007.pdb

jm4c00465_si_008.pdb

jm4c00465_si_009.pdb

jm4c00465_si_010.pdb

jm4c00465_si_011.pdb

jm4c00465_si_012.pdb

jm4c00465_si_013.pdb

jm4c00465_si_014.pdb

jm4c00465_si_015.pdb

## Abbreviations

cAMP: cyclic adenosine monophosphate
CB_1_R: cannabinoid receptor type 1
CB_2_R: cannabinoid receptor type 2
DEG: α,α-diethyl glycine
DIPEA: *N*,*N*-diisopropylethylamine
ECS: endocannabinoid system
EDCI: 1-ethyl-3-(3-(dimethylamino)propyl)carbodiimide hydrochloride
HATU: 1-[bis(dimethylamino)methylene]-1*H*-1,2,3-triazolo[4,5-*b*]pyridinium 3-oxide hexafluorophosphate
HEK: human embryonic kidney cells
HFIP: 1,1,1,3,3,3-hexafluoroisopropanol
HOAt: 1-hydroxy-7-azabenzotriazole
HTRF: homogeneous time-resolved fluorescence
IMHB: intramolecular hydrogen bonds
KHMDS: potassium bis(trimethylsilyl)amide
mCB: mouse cannabinoid receptor
MD: molecular dynamics
NBD-F: 4-fluoro-7-nitro-2,1,3-benzoxadiazole
PEG: polyethylene glycol
PSA: polar surface area
rCB: rat cannabinoid receptor
SOCl_2_: thionyl chloride
TAMRA-SE: carboxy-tetramethylrhodamin-*N*-succinimidylester
TEA: triethylamine
TM: trans membrane helix
TR-FRET: time-resolved Förster resonance energy transfer

## References

[ref1] Battista N.; Di Tommaso M.; Bari M.; Maccarrone M. The endocannabinoid system: an overview. Front. Behav. Neurosci. 2012, 6, 9 10.3389/fnbeh.2012.00009. 22457644 PMC3303140

[ref2] Zou S.; Kumar U. Cannabinoid Receptors and the Endocannabinoid System: Signaling and Function in the Central Nervous System. Int. J. Mol. Sci. 2018, 19 (3), 833 10.3390/ijms19030833. 29533978 PMC5877694

[ref3] Chou S.; Ranganath T.; Fish K. N.; Lewis D. A.; Sweet R. A. Cell type specific cannabinoid CB1 receptor distribution across the human and non-human primate cortex. Sci. Rep. 2022, 12 (1), 9605 10.1038/s41598-022-13724-x. 35688916 PMC9187707

[ref4] O’Sullivan S. E.; Yates A. S.; Porter R. K. The Peripheral Cannabinoid Receptor Type 1 (CB1) as a Molecular Target for Modulating Body Weight in Man. Molecules 2021, 26 (20), 6178 10.3390/molecules26206178. 34684760 PMC8538448

[ref5] Maccarrone M.; Bab I.; Biro T.; Cabral G. A.; Dey S. K.; Di Marzo V.; Konje J. C.; Kunos G.; Mechoulam R.; Pacher P.; et al. Endocannabinoid signaling at the periphery: 50 years after THC. Trends Pharmacol. Sci. 2015, 36 (5), 277–296. 10.1016/j.tips.2015.02.008. 25796370 PMC4420685

[ref6] Di Marzo V.; Goparaju S. K.; Wang L.; Liu J.; Bátkai S.; Járai Z.; Fezza F.; Miura G. I.; Palmiter R. D.; Sugiura T.; et al. Leptin-regulated endocannabinoids are involved in maintaining food intake. Nature 2001, 410 (6830), 822–825. 10.1038/35071088. 11298451

[ref7] Benard G.; Massa F.; Puente N.; Lourenco J.; Bellocchio L.; Soria-Gomez E.; Matias I.; Delamarre A.; Metna-Laurent M.; Cannich A.; et al. Mitochondrial CB(1) receptors regulate neuronal energy metabolism. Nat. Neurosci. 2012, 15 (4), 558–564. 10.1038/nn.3053. 22388959

[ref8] Toyoda H. CB1 cannabinoid receptor-mediated plasticity of GABAergic synapses in the mouse insular cortex. Sci. Rep. 2020, 10 (1), 7187 10.1038/s41598-020-64236-5. 32346039 PMC7189234

[ref9] Milligan A. L.; Szabo-Pardi T. A.; Burton M. D. Cannabinoid Receptor Type 1 and Its Role as an Analgesic: An Opioid Alternative?. J. Dual Diagn. 2020, 16 (1), 106–119. 10.1080/15504263.2019.1668100. 31596190 PMC7007359

[ref10] Marzo V. D.; Bifulco M.; Petrocellis L. D. The endocannabinoid system and its therapeutic exploitation. Nat. Rev. Drug Discovery 2004, 3 (9), 771–784. 10.1038/nrd1495. 15340387

[ref11] Pacher P.; Bátkai S.; Kunos G. The endocannabinoid system as an emerging target of pharmacotherapy. Pharmacol. Rev. 2006, 58 (3), 389–462. 10.1124/pr.58.3.2. 16968947 PMC2241751

[ref12] Amato G.; Khan N. S.; Maitra R. A patent update on cannabinoid receptor 1 antagonists (2015–2018). Expert Opin. Ther. Pat. 2019, 29 (4), 261–269. 10.1080/13543776.2019.1597851. 30889997 PMC6476312

[ref13] Vemuri V. K.; Makriyannis A. Medicinal chemistry of cannabinoids. Clin. Pharmacol. Ther. 2015, 97 (6), 553–558. 10.1002/cpt.115. 25801236 PMC4918805

[ref14] European Medicines Agency. Questions and Answers on the Recommendation to Suspend the Marketing Authorisation of Acomplia (Rimonabant), 2008, 23 October 2008. https://www.ema.europa.eu/en/documents/medicine-qa/questions-answers-recommendation-suspend-marketing-authorisation-acomplia-rimonabant_en.pdf (accessed Oct 31, 2023).

[ref15] Christensen R.; Kristensen P. K.; Bartels E. M.; Bliddal H.; Astrup A. Efficacy and safety of the weight-loss drug rimonabant: a meta-analysis of randomised trials. Lancet 2007, 370 (9600), 1706–1713. 10.1016/S0140-6736(07)61721-8. 18022033

[ref16] Cristino L.; Imperatore R.; Di Marzo V. Techniques for the Cellular and Subcellular Localization of Endocannabinoid Receptors and Enzymes in the Mammalian Brain. Methods Enzymol. 2017, 593, 61–98. 10.1016/bs.mie.2017.05.003. 28750816

[ref17] Bilic S.; Dagon Y.; Gustafson T.; Johnson L.; Lawler J.; Rudolph-Own L.; Gaich G.; Tucker E. OR03–2 Phase 1, Randomized, Controlled Trial of GFB-024, a Once-Monthly CB1 Inverse Agonist, in Healthy Overweight and Obese Participants and in Participants with Type 2 Diabetes Mellitus. J. Endocr. Soc. 2022, 6, A348 10.1210/jendso/bvac150.723.

[ref18] Crater G. D.; Lalonde K.; Ravenelle F.; Harvey M.; Despres J. P. Effects of CB1R inverse agonist, INV-202, in patients with features of metabolic syndrome. A randomized, placebo-controlled, double-blind phase 1b study. Diabetes Obes. Metab. 2024, 26 (2), 642–649. 10.1111/dom.15353. 37941317

[ref19] Galaj E.; Hempel B.; Moore A.; Klein B.; Bi G. H.; Gardner E. L.; Seltzman H. H.; Xi Z. X. Therapeutic potential of PIMSR, a novel CB1 receptor neutral antagonist, for cocaine use disorder: evidence from preclinical research. Transl. Psychiatry 2022, 12 (1), 286 10.1038/s41398-022-02059-w. 35851573 PMC9293959

[ref20] Cinar R.; Iyer M. R.; Kunos G. The therapeutic potential of second and third generation CB(1)R antagonists. Pharmacol. Ther. 2020, 208, 107477 10.1016/j.pharmthera.2020.107477. 31926199 PMC8605822

[ref21] Bosquez-Berger T.; Szanda G.; Straiker A. Requiem for Rimonabant: Therapeutic Potential for Cannabinoid CB1 Receptor Antagonists after the Fall. Drugs Drug Candidates 2023, 2 (3), 689–707. 10.3390/ddc2030035.

[ref22] Zawatsky C. N.; Park J. K.; Abdalla J.; Kunos G.; Iyer M. R.; Cinar R. Peripheral Hybrid CB(1)R and iNOS Antagonist MRI-1867 Displays Anti-Fibrotic Efficacy in Bleomycin-Induced Skin Fibrosis. Front. Endocrinol. 2021, 12, 744857 10.3389/fendo.2021.744857. PMC8505776 34650521

[ref23] Jacquot L.; Pointeau O.; Roger-Villeboeuf C.; Passilly-Degrace P.; Belkaid R.; Regazzoni I.; Leemput J.; Buch C.; Demizieux L.; Verges B.; et al. Therapeutic potential of a novel peripherally restricted CB1R inverse agonist on the progression of diabetic nephropathy. Front. Nephrol. 2023, 3, 1138416 10.3389/fneph.2023.1138416. 37675364 PMC10479578

[ref24] Monte A.; Gorbenko A.; Heuberger J.; Cundy K. C.; Klumpers L.; Groeneveld G. J. 304 Randomized Controlled Trial of ANEB-001 as an Antidote for Acute Cannabinoid Intoxication in Healthy Adults. Ann. Emerg. Med. 2023, 82 (4), S133 10.1016/j.annemergmed.2023.08.329.

[ref25] Busquets Garcia A.; Soria-Gomez E.; Bellocchio L.; Marsicano G. Cannabinoid receptor type-1: breaking the dogmas. F1000Res. 2016, 5, 990 10.12688/f1000research.8245.1. PMC4879932 27239293

[ref26] Stoddart L. A.; Kilpatrick L. E.; Briddon S. J.; Hill S. J. Probing the pharmacology of G protein-coupled receptors with fluorescent ligands. Neuropharmacology 2015, 98, 48–57. 10.1016/j.neuropharm.2015.04.033. 25979488

[ref27] Soave M.; Briddon S. J.; Hill S. J.; Stoddart L. A. Fluorescent ligands: Bringing light to emerging GPCR paradigms. Br. J. Pharmacol. 2020, 177 (5), 978–991. 10.1111/bph.14953. 31877233 PMC7042119

[ref28] Stoddart L. A.; Vernall A. J.; Denman J. L.; Briddon S. J.; Kellam B.; Hill S. J. Fragment screening at adenosine-A(3) receptors in living cells using a fluorescence-based binding assay. Chem. Biol. 2012, 19 (9), 1105–1115. 10.1016/j.chembiol.2012.07.014. 22999879 PMC3456874

[ref29] Cooper S. L.; Soave M.; Jorg M.; Scammells P. J.; Woolard J.; Hill S. J. Probe dependence of allosteric enhancers on the binding affinity of adenosine A(1) -receptor agonists at rat and human A(1) -receptors measured using NanoBRET. Br. J. Pharmacol. 2019, 176 (7), 864–878. 10.1111/bph.14575. 30644086 PMC6433648

[ref30] Bruno A.; Lembo F.; Novellino E.; Stornaiuolo M.; Marinelli L. Beyond radio-displacement techniques for identification of CB1 ligands: the first application of a fluorescence-quenching assay. Sci. Rep. 2014, 4, 3757 10.1038/srep03757. 24441508 PMC3895875

[ref31] Stoddart L. A.; Vernall A. J.; Briddon S. J.; Kellam B.; Hill S. J. Direct visualisation of internalization of the adenosine A3 receptor and localization with arrestin3 using a fluorescent agonist. Neuropharmacology 2015, 98, 68–77. 10.1016/j.neuropharm.2015.04.013. 25937210

[ref32] Hern J. A.; Baig A. H.; Mashanov G. I.; Birdsall B.; Corrie J. E.; Lazareno S.; Molloy J. E.; Birdsall N. J. Formation and dissociation of M1 muscarinic receptor dimers seen by total internal reflection fluorescence imaging of single molecules. Proc. Natl. Acad. Sci. U.S.A. 2010, 107 (6), 2693–2698. 10.1073/pnas.0907915107. 20133736 PMC2823895

[ref33] Cordeaux Y.; Briddon S. J.; Alexander S. P.; Kellam B.; Hill S. J. Agonist-occupied A3 adenosine receptors exist within heterogeneous complexes in membrane microdomains of individual living cells. FASEB J. 2008, 22 (3), 850–860. 10.1096/fj.07-8180com. 17959910

[ref34] Hamilton A. J.; Payne A. D.; Mocerino M.; Gunosewoyo H. Imaging Cannabinoid Receptors: A Brief Collection of Covalent and Fluorescent Probes for CB. Aust. J. Chem. 2021, 74 (6), 416–432. 10.1071/CH21007.

[ref35] Amenta A.; Caprioglio D.; Minassi A.; Panza L.; Passarella D.; Fasano V.; Imperio D. Recent advances in the development of CB1R selective probes. Front. Nat. Prod. 2023, 2, 1196321 10.3389/fntpr.2023.1196321.

[ref36] Prokop S.; Abranyi-Balogh P.; Barti B.; Vamosi M.; Zoldi M.; Barna L.; Urban G. M.; Toth A. D.; Dudok B.; Egyed A.; et al. PharmacoSTORM nanoscale pharmacology reveals cariprazine binding on Islands of Calleja granule cells. Nat. Commun. 2021, 12 (1), 6505 10.1038/s41467-021-26757-z. 34764251 PMC8586358

[ref37] Martin-Fontecha M.; Angelina A.; Ruckert B.; Rueda-Zubiaurre A.; Martin-Cruz L.; van de Veen W.; Akdis M.; Ortega-Gutierrez S.; Lopez-Rodriguez M. L.; Akdis C. A.; et al. A Fluorescent Probe to Unravel Functional Features of Cannabinoid Receptor CB(1) in Human Blood and Tonsil Immune System Cells. Bioconjugate Chem. 2018, 29 (2), 382–389. 10.1021/acs.bioconjchem.7b00680. 29314831

[ref38] Grant P. S.; Kahlcke N.; Govindpani K.; Hunter M.; MacDonald C.; Brimble M. A.; Glass M.; Furkert D. P. Divalent cannabinoid-1 receptor ligands: A linker attachment point survey of SR141716A for development of high-affinity CB1R molecular probes. Bioorg. Med. Chem. Lett. 2019, 29 (21), 126644 10.1016/j.bmcl.2019.126644. 31564385

[ref39] Daly C. J.; Ross R. A.; Whyte J.; Henstridge C. M.; Irving A. J.; McGrath J. C. Fluorescent ligand binding reveals heterogeneous distribution of adrenoceptors and ’cannabinoid-like’ receptors in small arteries. Br. J. Pharmacol. 2010, 159 (4), 787–796. 10.1111/j.1476-5381.2009.00608.x. 20136833 PMC2829204

[ref40] Coutts A. A.; Anavi-Goffer S.; Ross R. A.; MacEwan D. J.; Mackie K.; Pertwee R. G.; Irving A. J. Agonist-induced internalization and trafficking of cannabinoid CB1 receptors in hippocampal neurons. J. Neurosci. 2001, 21 (7), 2425–2433. 10.1523/JNEUROSCI.21-07-02425.2001. 11264316 PMC6762401

[ref41] Guberman M.; Kosar M.; Omran A.; Carreira E. M.; Nazaré M.; Grether U. Reverse-Design toward Optimized Labeled Chemical Probes - Examples from the Endocannabinoid System. Chimia 2022, 76 (5), 425 10.2533/chimia.2022.425. 38069714

[ref42] Gazzi T.; Brennecke B.; Atz K.; Korn C.; Sykes D.; Forn-Cuni G.; Pfaff P.; Sarott R. C.; Westphal M. V.; Mostinski Y.; et al. Detection of cannabinoid receptor type 2 in native cells and zebrafish with a highly potent, cell-permeable fluorescent probe. Chem. Sci. 2022, 13 (19), 5539–5545. 10.1039/D1SC06659E. 35694350 PMC9116301

[ref43] Slavik R.; Grether U.; Müller Herde A.; Gobbi L.; Fingerle J.; Ullmer C.; Krämer S. D.; Schibli R.; Mu L. J.; Ametamey S. M. Discovery of a High Affinity and Selective Pyridine Analog as a Potential Positron Emission Tomography Imaging Agent for Cannabinoid Type 2 Receptor. J. Med. Chem. 2015, 58 (10), 4266–4277. 10.1021/acs.jmedchem.5b00283. 25950914

[ref44] Haider A.; Kretz J.; Gobbi L.; Ahmed H.; Atz K.; Burkler M.; Bartelmus C.; Fingerle J.; Guba W.; Ullmer C.; et al. Structure-Activity Relationship Studies of Pyridine-Based Ligands and Identification of a Fluorinated Derivative for Positron Emission Tomography Imaging of Cannabinoid Type 2 Receptors. J. Med. Chem. 2019, 62 (24), 11165–11181. 10.1021/acs.jmedchem.9b01280. 31751140

[ref45] Haider A.; Gobbi L.; Kretz J.; Ullmer C.; Brink A.; Honer M.; Woltering T. J.; Muri D.; Iding H.; Bürkler M.; et al. Identification and Preclinical Development of a 2,5,6-Trisubstituted Fluorinated Pyridine Derivative as a Radioligand for the Positron Emission Tomography Imaging of Cannabinoid Type 2 Receptors. J. Med. Chem. 2020, 63 (18), 10287–10306. 10.1021/acs.jmedchem.0c00778. 32787079

[ref46] Sasmal P. K.; Reddy D. S.; Talwar R.; Venkatesham B.; Balasubrahmanyam D.; Kannan M.; Srinivas P.; Kyasa S. K.; Devi B. N.; Jadhav V. P.; et al. Novel pyrazole-3-carboxamide derivatives as cannabinoid-1 (CB1) antagonists: journey from non-polar to polar amides. Bioorg. Med. Chem. Lett. 2011, 21 (1), 562–568. 10.1016/j.bmcl.2010.10.055. 21075633

[ref47] Mayweg A.; Narquizian R.; Pflieger P.; Roever S. Heterocyclic cannabinoid receptor antagonists. U.S. Patent 7,563,910 B2, 2009.

[ref48] Rinaldi-Carmona M.; Barth F.; Heaulme M.; Shire D.; Calandra B.; Congy C.; Martinez S.; Maruani J.; Neliat G.; Caput D.; et al. SR141716A, a potent and selective antagonist of the brain cannabinoid receptor. FEBS Lett. 1994, 350 (2–3), 240–244. 10.1016/0014-5793(94)00773-X. 8070571

[ref49] Lazzari P.; Distinto R.; Manca I.; Baillie G.; Murineddu G.; Pira M.; Falzoi M.; Sani M.; Morales P.; Ross R.; et al. A critical review of both the synthesis approach and the receptor profile of the 8-chloro-1-(2 ’,4 ’-dichlorophenyl)-N-piperidin-1-yl-1,4,5,6-tetrahydrobenzo[6,7]cyclohepta[1,2-c]pyrazole-3-carboxamide and analogue derivatives. Eur. J. Med. Chem. 2016, 121, 194–208. 10.1016/j.ejmech.2016.05.011. 27240274

[ref50] Rover S.; Andjelkovic M.; Benardeau A.; Chaput E.; Guba W.; Hebeisen P.; Mohr S.; Nettekoven M.; Obst U.; Richter W. F.; et al. 6-Alkoxy-5-aryl-3-pyridinecarboxamides, a new series of bioavailable cannabinoid receptor type 1 (CB1) antagonists including peripherally selective compounds. J. Med. Chem. 2013, 56 (24), 9874–9896. 10.1021/jm4010708. 24175572

[ref51] Hebeisen P.; Iding H.; Nettekoven Matthias H.; Sander Ulrike O.; Roever S.; Weiss U. R. S.; Wirz B. Pyrazinecarboxamide derivatives as CB1 antagonists. U.S. Patent 7,629,346 B2, 2009.

[ref52] Schoeder C. T.; Hess C.; Madea B.; Meiler J.; Muller C. E. Pharmacological evaluation of new constituents of ″Spice″: synthetic cannabinoids based on indole, indazole, benzimidazole and carbazole scaffolds. Forensic Toxicol. 2018, 36 (2), 385–403. 10.1007/s11419-018-0415-z. 29963207 PMC6002460

[ref53] Katritzky A. R.; Todadze E.; Angrish P.; Draghici B. Efficient Peptide Coupling Involving Sterically Hindered Amino Acids. J. Org. Chem. 2007, 72 (15), 5794–5801. 10.1021/jo0704255. 17580899

[ref54] Benson S.; Fernandez A.; Barth N. D.; de Moliner F.; Horrocks M. H.; Herrington C. S.; Abad J. L.; Delgado A.; Kelly L.; Chang Z.; et al. SCOTfluors: Small, Conjugatable, Orthogonal, and Tunable Fluorophores for In Vivo Imaging of Cell Metabolism. Angew. Chem., Int. Ed. 2019, 58 (21), 6911–6915. 10.1002/anie.201900465. PMC6563150 30924239

[ref55] Choy J.; Jaime-Figueroa S.; Jiang L.; Wagner P. Novel Practical Deprotection of N-Boc Compounds Using Fluorinated Alcohols. Synth. Commun. 2008, 38 (21), 3840–3853. 10.1080/00397910802238718.

[ref56] Hua T.; Vemuri K.; Pu M.; Qu L.; Han G. W.; Wu Y.; Zhao S.; Shui W.; Li S.; Korde A.; et al. Crystal Structure of the Human Cannabinoid Receptor CB(1). Cell 2016, 167 (3), 750–762.e14. 10.1016/j.cell.2016.10.004. 27768894 PMC5322940

[ref57] Banister S. D.; Moir M.; Stuart J.; Kevin R. C.; Wood K. E.; Longworth M.; Wilkinson S. M.; Beinat C.; Buchanan A. S.; Glass M.; et al. Pharmacology of Indole and Indazole Synthetic Cannabinoid Designer Drugs AB-FUBINACA, ADB-FUBINACA, AB-PINACA, ADB-PINACA, 5F-AB-PINACA, 5F-ADB-PINACA, ADBICA, and 5F-ADBICA. ACS Chem. Neurosci. 2015, 6 (9), 1546–1559. 10.1021/acschemneuro.5b00112. 26134475

[ref58] Degorce F.; Card A.; Soh S.; Trinquet E.; Knapik G. P.; Xie B. HTRF: A technology tailored for drug discovery - a review of theoretical aspects and recent applications. Curr. Chem. Genomics 2009, 3, 22–32. 10.2174/1875397300903010022. 20161833 PMC2802762

[ref59] Singh S.; Oyagawa C. R. M.; Macdonald C.; Grimsey N. L.; Glass M.; Vernall A. J. Chromenopyrazole-based High Affinity, Selective Fluorescent Ligands for Cannabinoid Type 2 Receptor. ACS Med. Chem. Lett. 2019, 10 (2), 209–214. 10.1021/acsmedchemlett.8b00597. 30783505 PMC6378661

[ref60] Cooper A. G.; Oyagawa C. R. M.; Manning J. J.; Singh S.; Hook S.; Grimsey N. L.; Glass M.; Tyndall J. D. A.; Vernall A. J. Development of selective, fluorescent cannabinoid type 2 receptor ligands based on a 1,8-naphthyridin-2-(1*H*)-one-3-carboxamide scaffold. MedChemComm 2018, 9 (12), 2055–2067. 10.1039/C8MD00448J. 30647881 PMC6301273

[ref61] Spinelli F.; Giampietro R.; Stefanachi A.; Riganti C.; Kopecka J.; Abatematteo F. S.; Leonetti F.; Colabufo N. A.; Mangiatordi G. F.; Nicolotti O.; et al. Design and synthesis of fluorescent ligands for the detection of cannabinoid type 2 receptor (CB2R). Eur. J. Med. Chem. 2020, 188, 112037 10.1016/j.ejmech.2020.112037. 31954990

[ref62] Baker J. G.; Middleton R.; Adams L.; May L. T.; Briddon S. J.; Kellam B.; Hill S. J. Influence of fluorophore and linker composition on the pharmacology of fluorescent adenosine A1 receptor ligands. Br. J. Pharmacol. 2010, 159 (4), 772–786. 10.1111/j.1476-5381.2009.00488.x. 20105183 PMC2829203

[ref63] Yates A. S.; Doughty S. W.; Kendall D. A.; Kellam B. Chemical modification of the naphthoyl 3-position of JWH-015: in search of a fluorescent probe to the cannabinoid CB2 receptor. Bioorg. Med. Chem. Lett. 2005, 15 (16), 3758–3762. 10.1016/j.bmcl.2005.05.049. 15993070

[ref64] Lipinski C. A.; Lombardo F.; Dominy B. W.; Feeney P. J. Experimental and computational approaches to estimate solubility and permeability in drug discovery and development settings 1PII of original article: S0169-409X(96)00423-1. The article was originally published in Advanced Drug Delivery Reviews 23 (1997) 3–25. 1. Adv. Drug Delivery Rev. 2001, 46 (1–3), 3–26. 10.1016/S0169-409X(00)00129-0. 11259830

[ref65] Veber D. F.; Johnson S. R.; Cheng H. Y.; Smith B. R.; Ward K. W.; Kopple K. D. Molecular properties that influence the oral bioavailability of drug candidates. J. Med. Chem. 2002, 45 (12), 2615–2623. 10.1021/jm020017n. 12036371

[ref66] Wang L.; Frei M. S.; Salim A.; Johnsson K. Small-Molecule Fluorescent Probes for Live-Cell Super-Resolution Microscopy. J. Am. Chem. Soc. 2019, 141 (7), 2770–2781. 10.1021/jacs.8b11134. 30550714

[ref67] Wang L.; Tran M.; D’Este E.; Roberti J.; Koch B.; Xue L.; Johnsson K. A general strategy to develop cell permeable and fluorogenic probes for multicolour nanoscopy. Nat. Chem. 2020, 12 (2), 165–172. 10.1038/s41557-019-0371-1. 31792385

[ref68] Matsson P.; Doak B. C.; Over B.; Kihlberg J. Cell permeability beyond the rule of 5. Adv. Drug Delivery Rev. 2016, 101, 42–61. 10.1016/j.addr.2016.03.013. 27067608

[ref69] David L.; Wenlock M.; Barton P.; Ritzen A. Prediction of Chameleonic Efficiency. ChemMedChem 2021, 16 (17), 2669–2685. 10.1002/cmdc.202100306. 34240561

[ref70] Poongavanam V.; Wieske L. H. E.; Peintner S.; Erdelyi M.; Kihlberg J. Molecular chameleons in drug discovery. Nat. Rev. Chem 2024, 8 (1), 45–60. 10.1038/s41570-023-00563-1. 38123688

[ref71] Daina A.; Michielin O.; Zoete V. SwissADME: a free web tool to evaluate pharmacokinetics, drug-likeness and medicinal chemistry friendliness of small molecules. Sci. Rep. 2017, 7, 42717 10.1038/srep42717. 28256516 PMC5335600

[ref72] Poongavanam V.; Atilaw Y.; Siegel S.; Giese A.; Lehmann L.; Meibom D.; Erdelyi M.; Kihlberg J. Linker-Dependent Folding Rationalizes PROTAC Cell Permeability. J. Med. Chem. 2022, 65 (19), 13029–13040. 10.1021/acs.jmedchem.2c00877. 36170570 PMC9574858

[ref73] Sykes D. A.; Borrega-Roman L.; Harwood C. R.; Hoare B.; Lochray J. M.; Gazzi T.; Briddon S. J.; Nazaré M.; Grether U.; Hill S. J.; Kinetic Profiling of Ligands and Fragments Binding to GPCRs by TR-FRET. In Biophysical and Computational Tools in Drug Discovery; Saxena A. K., Ed.; Springer International Publishing, 2021; pp 1–32.

[ref74] Navarro G.; Sotelo E.; Raich I.; Loza M. I.; Brea J.; Majellaro M. A Robust and Efficient FRET-Based Assay for Cannabinoid Receptor Ligands Discovery. Molecules 2023, 28 (24), 8107 10.3390/molecules28248107. 38138600 PMC10745346

[ref75] Raich I.; Rivas-Santisteban R.; Lillo A.; Lillo J.; Reyes-Resina I.; Nadal X.; Ferreiro-Vera C.; de Medina V. S.; Majellaro M.; Sotelo E.; et al. Similarities and differences upon binding of naturally occurring Δ9-tetrahydrocannabinol-derivatives to cannabinoid CB1 and CB2 receptors. Pharmacol. Res. 2021, 174, 105970 10.1016/j.phrs.2021.105970. 34758399

[ref76] Schiele F.; Ayaz P.; Fernandez-Montalvan A. A universal homogeneous assay for high-throughput determination of binding kinetics. Anal. Biochem. 2015, 468, 42–49. 10.1016/j.ab.2014.09.007. 25240173

[ref77] Sykes D. A.; Stoddart L. A.; Kilpatrick L. E.; Hill S. J. Binding kinetics of ligands acting at GPCRs. Mol. Cell. Endocr. 2019, 485, 9–19. 10.1016/j.mce.2019.01.018. PMC6406023 30738950

[ref78] Copeland R. A.; Pompliano D. L.; Meek T. D. Drug-target residence time and its implications for lead optimization. Nat. Rev. Drug Discovery 2006, 5 (9), 730–739. 10.1038/nrd2082. 16888652

[ref79] Muccioli G. G.; Wouters J.; Charlier C.; Scriba G. K.; Pizza T.; Di Pace P.; De Martino P.; Poppitz W.; Poupaert J. H.; Lambert D. M. Synthesis and activity of 1,3,5-triphenylimidazolidine-2,4-diones and 1,3,5-triphenyl-2-thioxoimidazolidin-4-ones: characterization of new CB1 cannabinoid receptor inverse agonists/antagonists. J. Med. Chem. 2006, 49 (3), 872–882. 10.1021/jm050484f. 16451053

[ref80] Manera C.; Cascio M. G.; Benetti V.; Allara M.; Tuccinardi T.; Martinelli A.; Saccomanni G.; Vivoli E.; Ghelardini C.; Di Marzo V.; et al. New 1,8-naphthyridine and quinoline derivatives as CB2 selective agonists. Bioorg. Med. Chem. Lett. 2007, 17 (23), 6505–6510. 10.1016/j.bmcl.2007.09.089. 17942307

[ref81] Joharapurkar A.; Raval S.; Patel J. Z.; Soni R.; Raval P.; Gite A.; Goswami A.; Sadhwani N.; Gandhi N.; et al. Diaryl dihydropyrazole-3-carboxamides with significant in vivo antiobesity activity related to CB1 receptor antagonism: synthesis, biological evaluation, and molecular modeling in the homology model. J. Med. Chem. 2007, 50 (24), 5951–5966. 10.1021/jm061490u. 17979261

[ref82] Donohue S. R.; Pike V. W.; Finnema S. J.; Truong P.; Andersson J.; Gulyás B.; Halldin C. Discovery and labeling of high-affinity 3,4-diarylpyrazolines as candidate radioligands for in vivo imaging of cannabinoid subtype-1 (CB1) receptors. J. Med. Chem. 2008, 51 (18), 5608–5616. 10.1021/jm800329z. 18754613 PMC4182912

[ref83] Cowart M.; Gfesser G. A.; Bhatia K.; Esser R.; Sun M.; Miller T. R.; Krueger K.; Witte D.; Esbenshade T. A.; Hancock A. A. Fluorescent benzofuran histamine H(3) receptor antagonists with sub-nanomolar potency. Inflamm. Res. 2006, 55, S47–S48. 10.1007/s00011-005-0036-y. 16705379

[ref84] Sezgin E.; Can F. B.; Schneider F.; Clausen M. P.; Galiani S.; Stanly T. A.; Waithe D.; Colaco A.; Honigmann A.; Wustner D.; et al. A comparative study on fluorescent cholesterol analogs as versatile cellular reporters. J. Lipid Res. 2016, 57 (2), 299–309. 10.1194/jlr.M065326. 26701325 PMC4727425

[ref85] Smith B. A.; O’Neil E. J.; Lampkins A. J.; Johnson J. R.; Lee J. J.; Cole E. L.; Smith B. D. Evaluation of fluorescent phosphatidylserine substrates for the aminophospholipid flippase in mammalian cells. J. Fluoresc. 2012, 22 (1), 93–101. 10.1007/s10895-011-0933-0. 21814762 PMC3703462

[ref86] Rozenfeld R.; Devi L. A. Regulation of CB1 cannabinoid receptor trafficking by the adaptor protein AP-3. FASEB J. 2008, 22 (7), 2311–2322. 10.1096/fj.07-102731. 18267983 PMC3127579

[ref87] Wu D. F.; Yang L. Q.; Goschke A.; Stumm R.; Brandenburg L. O.; Liang Y. J.; Hollt V.; Koch T. Role of receptor internalization in the agonist-induced desensitization of cannabinoid type 1 receptors. J. Neurochem. 2008, 104 (4), 1132–1143. 10.1111/j.1471-4159.2007.05063.x. 17986216

[ref88] Cheng Y.-C.; Prusoff W. H. Relationship between Inhibition Constant (K1) and Concentration of Inhibitor Which Causes 50 Per Cent Inhibition (I50) of an Enzymatic-Reaction. Biochem. Pharmacol. 1973, 22 (23), 3099–3108. 10.1016/0006-2952(73)90196-2. 4202581

[ref89] Fulmer G. R.; Miller A. J. M.; Sherden N. H.; Gottlieb H. E.; Nudelman A.; Stoltz B. M.; Bercaw J. E.; Goldberg K. I. NMR Chemical Shifts of Trace Impurities: Common Laboratory Solvents, Organics, and Gases in Deuterated Solvents Relevant to the Organometallic Chemist. Organometallics 2010, 29 (9), 2176–2179. 10.1021/om100106e.

[ref90] Murineddu G.; Lazzari P.; Ruiu S.; Sanna A.; Loriga G.; Manca I.; Falzoi M.; Dessì C.; Curzu M. M.; Chelucci G.; et al. Tricyclic pyrazoles: 4.: Synthesis and biological evaluation of analogues of the robust and selective CB cannabinoid ligand 1-(2′,4′-dichlorophenyl)-6-methyl-piperidin-1-yl-1,4-dihydroindeno[1,2-c]pyrazole-3-carboxamide. J. Med. Chem. 2006, 49 (25), 7502–7512. 10.1021/jm060920d. 17149879

[ref91] Longworth M.; Banister S. D.; Boyd R.; Kevin R. C.; Connor M.; McGregor I. S.; Kassiou M. Pharmacology of Cumyl-Carboxamide Synthetic Cannabinoid New Psychoactive Substances (NPS) CUMYL-BICA, CUMYL-PICA, CUMYL-5F-PICA, CUMYL-5F-PINACA, and Their Analogues. ACS Chem. Neurosci. 2017, 8 (10), 2159–2167. 10.1021/acschemneuro.7b00267. 28792725

