# Supplementary material for: Highly Selective Drug-Derived Fluorescent Probes for the Cannabinoid Receptor Type 1 (CB1R)
Source: J Med Chem. 2024 Jul 11;67(14):11841–67. doi: 10.1021/acs.jmedchem.4c00465 (PMC11284800; doi:10.1021/acs.jmedchem.4c00465)
Supplement: Supplementary file 2 — jm4c00465_si_002.pdf [file jm4c00465_si_002.pdf]

Supporting Information

Highly Selective Drug-Derived Fluorescent Probes for the Cannabinoid Receptor Type 1 (CB_1R)

Leonard Mach,[a] Anahid Omran,[a] Jara Bouma,[b] Silke Radetzki,[a] David A. Sykes,[c,d] Wolfgang Guba,[e] Xiaoting Li,[f,g] Calvin Höffelmeyer,[a] Axel Hentsch,[a] Thais Gazzi,[a] Yelena Mostinski,[a] Malgorzata Wasinska-Kalwa,[a] Fabio de Molnier,[h] Cas van der Horst,[b] Jens Peter von Kries,[a] Marc Vendrell,[h] Tian Hua,[f,g] Dmitry B. Veprintsev,[c,d] Laura H. Heitman,[b] Uwe Grether,[e] Marc Nazare[a]**

[a] Leibniz-Forschungsinstitut für Molekulare Pharmakologie (FMP), 13125 Berlin (Germany)

[b] Division of Drug Discovery and Safety, Leiden Academic Centre for Drug Research, Leiden University, and Oncode Institute, 2333 CC, Leiden (The Netherlands)

[c] Division of Physiology, Pharmacology & Neuroscience, School of Life Sciences, University of Nottingham, Nottingham, NG7 2UH (United Kingdom)

[d] Centre of Membrane Proteins and Receptors (COMPARE), University of Birmingham and University of Nottingham, Edgbaston, B15 2TT Birmingham, Midlands (United Kingdom)

[e] Roche Pharma Research & Early Development, Roche Innovation Center Basel, F. Hoffmann-La Roche Ltd., 4070 Basel (Switzerland)

[f] iHuman Institute, ShanghaiTech University, Shanghai 201210 (China)

[g] School of Life Science and Technology, ShanghaiTech University, Shanghai 201210 (China)

[h] IRR Chemistry Hub and Centre for Inflammation Research, Institute for Regeneration and Repair, University of Edinburgh, EH16 4UU Edinburgh (United Kingdom)

Corresponding Authors

Uwe Grether - E-mail: uwe.grether@roche.com

Marc Nazare - nazare@fmp-berlin.de

Contents

In vitro pharmacology 4

Imaging Experiments 8

Computational Docking 14

Conformational Molecular Dynamics (MD) Simulations 17

Photophysical Characterization 26

NMR spectra 27

HPLC purity 29

References 35

In vitro Pharmacology

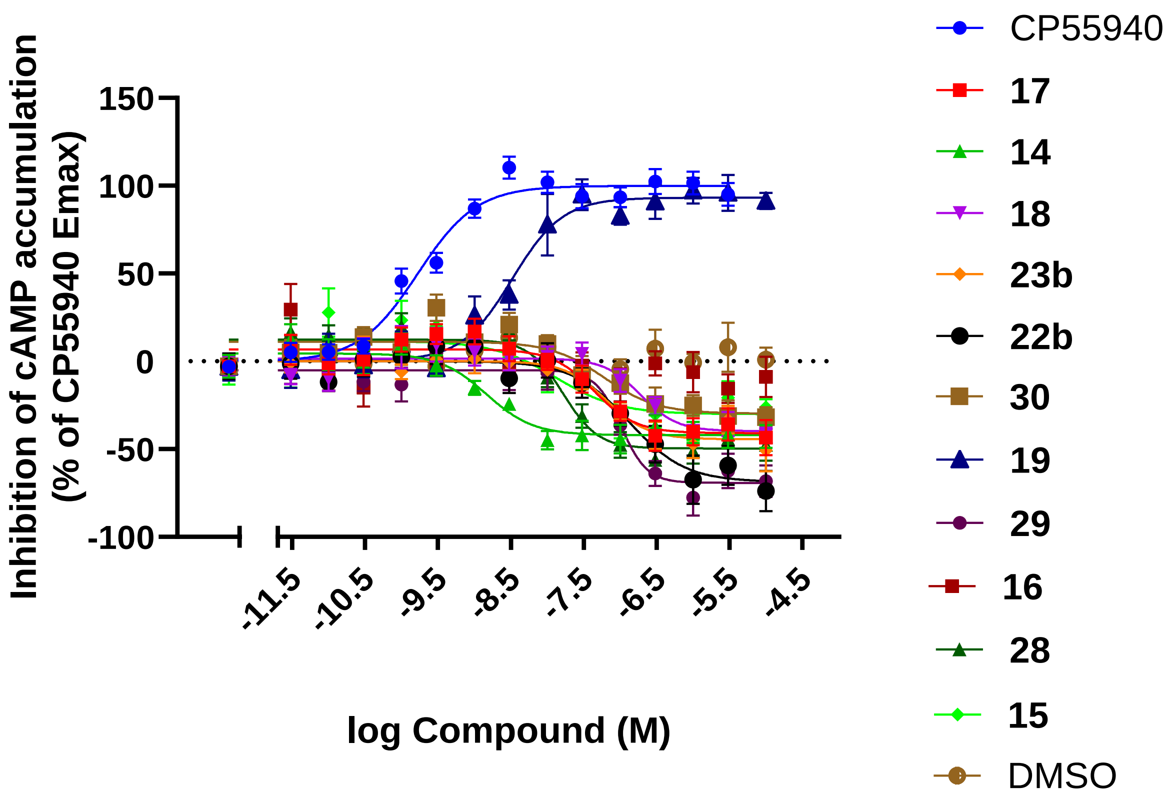

Figure S1. Functional agonist and inverse agonist activity of compounds in the HTRF cAMP assay with CP55,940 as reference.

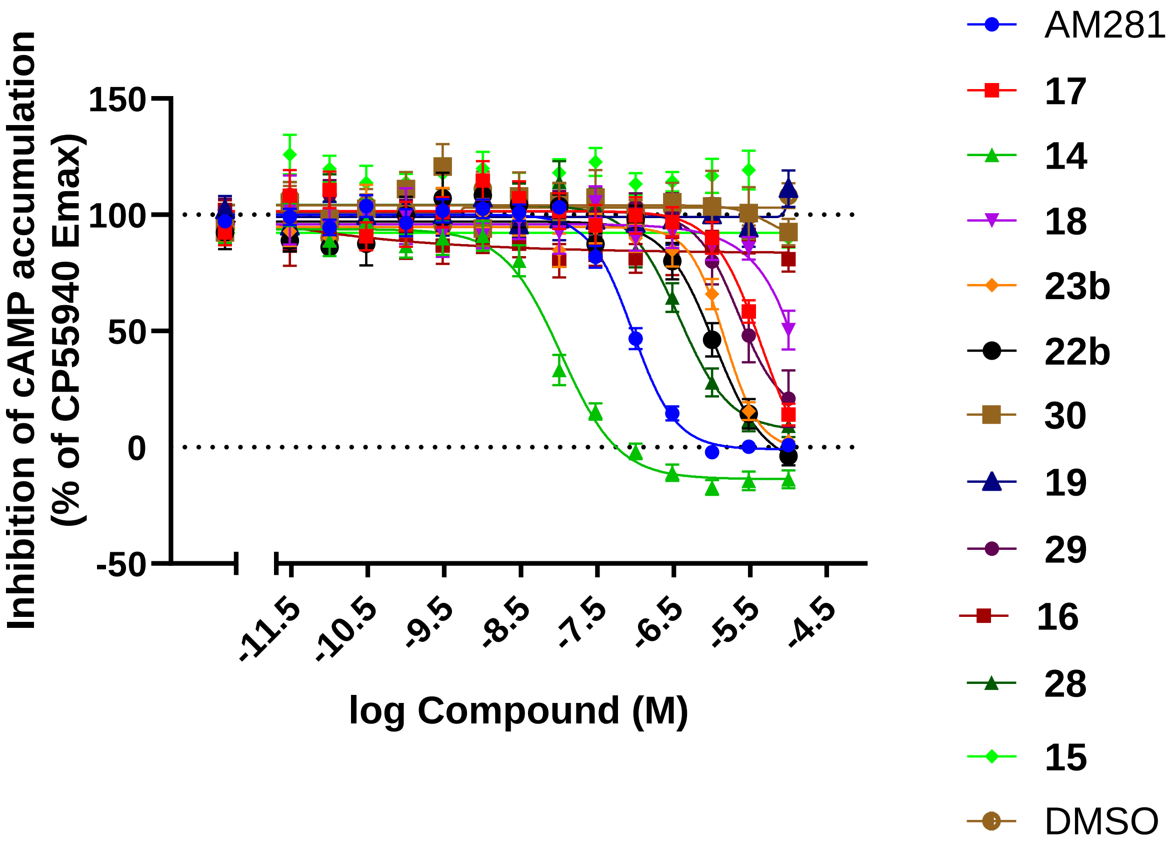

Figure S2. Antagonist activity of compounds in the HTRF cAMP assay with normalization to antagonist AM281 as reference.

Table S1. Overview of binding affinity and activity expressed as pK_i and pEC$_{50}$/pIC$_{50}$.

Compound	Binding affinity (Radioligand competition)		Functional activity CB$_1$R (cAMP assay)
	hCB$_1$R pK_i ± SEM or % displacement at 10 µM[a]	hCB$_2$R pK_i ± SEM or % displacement at 10 µM[a]	pEC$_{50}$ or pIC$_{50}$ ± SEM[b]
**14**	7.96 ± 0.13	6.51 ± 0.04	8.83 ± 0.16
**15**	7.55 ± 0.04	8.07 ± 0.06	7.88 ± 0.32
**16**	7.75 ± 0.07	8.40 ± 0.12	n.a.
**17**	6.51 ± 0.03	8.19 ± 0.01	7.36 ± 0.17
**18**	6.09 ± 0.03	7.95 ± 0.04	6.72 ± 0.17
**19**	7.72 ± 0.04	9.20 ± 0.01	8.49 ± 0.11
**22a**	5.85 ± 0.38	20 ± 3	n.d.
**22b**	6.09 ± 0.34	22 ± 9	6.88 ± 0.20
**22c**	5.91 ± 0.02	23 ± 5	n.d.

**23a**	7.43 ± 0.01	5.80 ± 0.12	n.d.
**23b**	6.86 ± 0.14	44 ± 5	7.19 ± 0.16
**23c**	6.40 ± 0.04	5.67 ± 0.03	n.d.
**23d**	6.22 ± 0.07	5.67 ± 0.05	n.d.
**24**	28 ± 3	6.11 ± 0.2	n.d.
**25**	5.52 ± 0.09	6.09 ± 0.10	n.d.
**26**	32 ± 6	6.15 ± 0.11	n.d.
**27**	48 ± 2	6.97 ± 0.17	n.d.
**28**	7.01 ± 0.18	41 ± 2	7.78 ± 0.10
**29**	5.68 ± 0.29	18 ± 5	6.99 ± 0.09
**30**	6.37 ± 0.08	49 ± 3	7.22 ± 0.21
**31**	37 ± 5	32 ± 6	n.d.
**32**	35 ± 6	6.51 ± 0.15	n.d.
**33**	-5 ± 1	21 ± 3	n.d.
**40**	34 ± 1	6.32 ± 0.01	n.d.
**41**	9 ± 1	27 ± 3	n.d.
**34**	32 ± 6	6.15 ± 0.11	n.d.
**35**	-6 ± 5	2 ± 0	n.d.
**36**	5.93 ± 0.15	7.1 ± 0.02	n.d.
**37**	18 ± 2	6.01 ± 0.08	n.d.
**51**	6.50 ± 0.05	6.31 ± 0.09	n.d.
**52**	6.65 ± 0.09	5.79 ± 0.16	n.d.

[a]pK_i or % displacement values obtained from [^{3}H]CP55,940 displacement assays on CHO membranes stably expressing human CB_1R or human CB_2R. Values are means ± standard error of the mean (SEM) of three independent experiments performed in duplicate. [b]The activity levels (pEC_{50} or pIC_{50}) of compounds were measured using cells stably expressing hCB_1R in homogeneous time-resolved fluorescence (HTRF®) cAMP assay. The data are the means of three independent experiments performed in technical replicates. n.d. is not determined. n.a. is no activity.

Table S2. Overview of antagonist activity expressed as pEC_{50}/pIC_{50}.

**Compound**	**Functional activity CB_1R (cAMP assay)**	
	**pEC_{50} or pIC_{50} ± SEM[a]**	**Variation of Emax**
**AM281**	7.0 ± 0.04	-100
**14**	8.0 ± 0.06	-107
**15**	n.a.	n.a.
**16**	n.a.	n.a.
**17**	5.4 ± 0.37	-116
**18**	n.a.	n.a.

**19**	n.a.	n.a.
**22b**	6.0 ± 0.12	-105
**23b**	5.8 ± 0.08	-96
**28**	6.4 ± 0.10	-97
**29**	5.7 ± 0.16	-88
**30**	n.a.	n.a.

[a]The activity levels (pEC_{50} or pIC_{50}) of compounds were measured using cells stably expressing hCB_1R in homogeneous time-resolved fluorescence (HTRF) cAMP assay. The data are the means ± standard error of the mean (SEM) of three independent experiments performed in technical replicates. [c]Maximum effect (E_{max} in %) was normalized to reference antagonist AM281. n.a. is no activity.

Table S3. Overview of TR-FRET binding experiment results expressed as pK_i/pK_D.

Compd.	Kinetic pK_D (nM)	pK_i (nM)[b]	pK_i (nM)[c]
**6**	8.50 ± 0.07	8.48 ± 0.04	8.68 ± 0.02
HU210	8.52 ± 0.05	8.69 ± 0.05	8.90 ± 0.03

[a]Values are means ± standard error of the mean (SEM) of N = 3. [b]Probe **29** (300 nM) used as tracer. [c]Probe **28** (60 nM) used as tracer.

Imaging Experiments

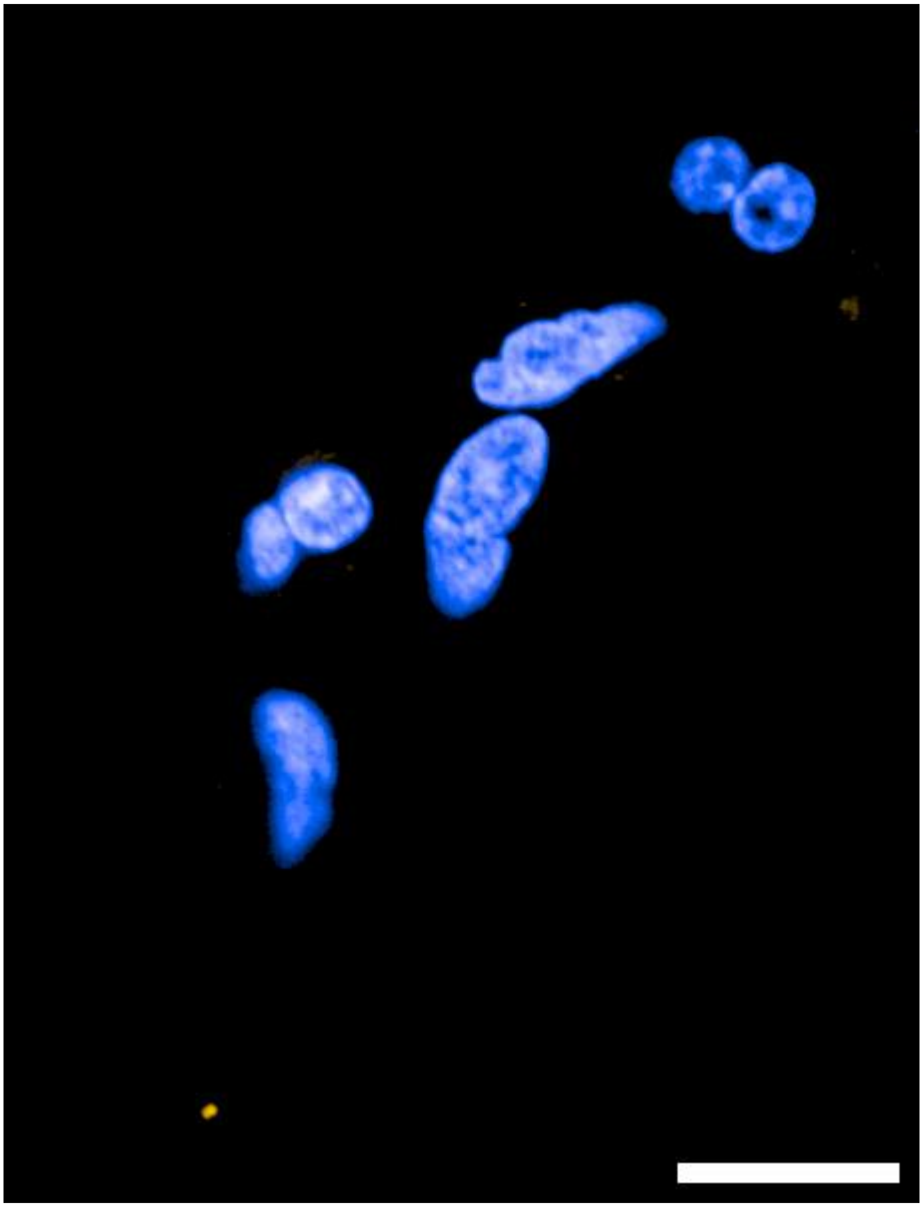

Figure S3. Live cell confocal imaging of uninduced CB_2R-HEK293TR cells (no CB_2R-expression) incubated with **29** (250 nM, yellow) at 63x magnification. Images recorded after 10 min. Nucleus counter stain: Hoechst 33342 (blue). Scale bars 20 µm. Images are representative of two to three independent experiments.

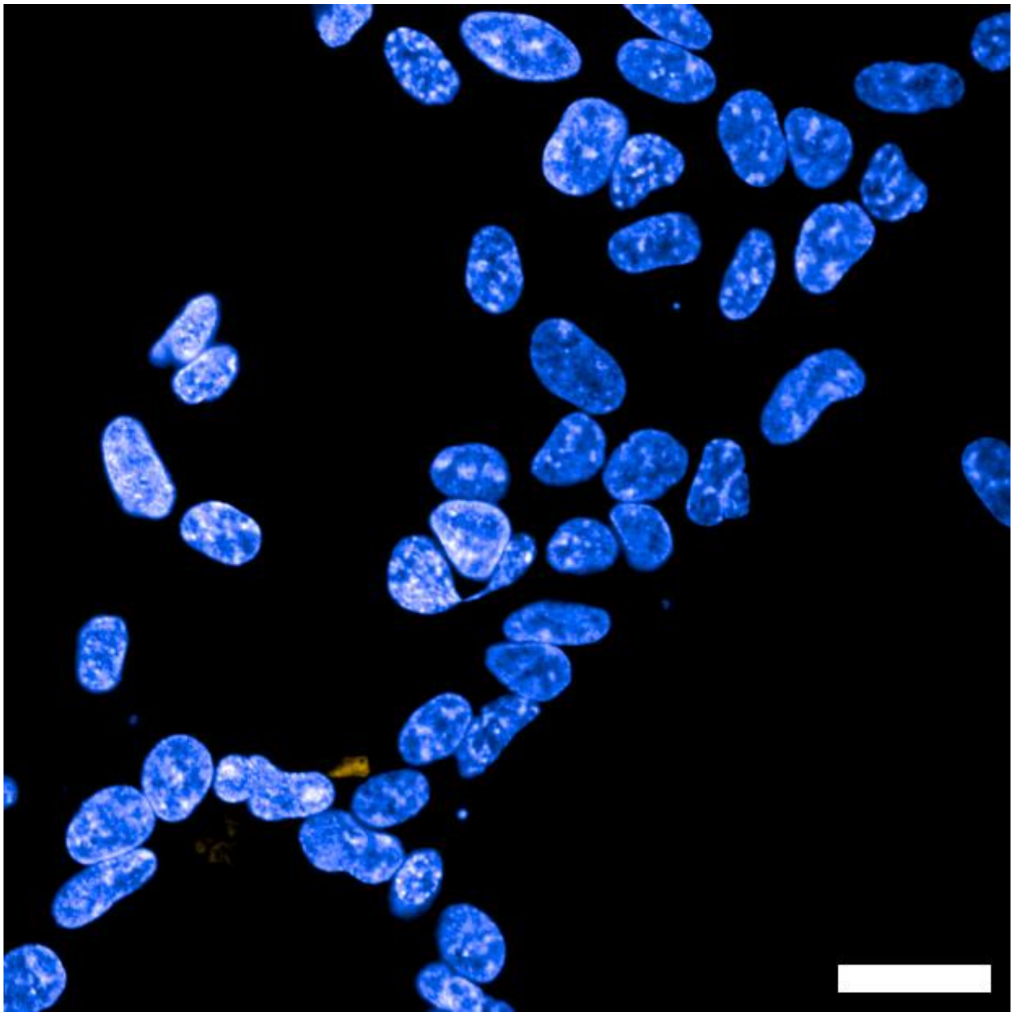

Figure S4. Live cell confocal imaging of HEK293TR cells (no CBR) incubated with **29** (250 nM, yellow) at 63x magnification. Images recorded after 10 min. Nucleus counter stain: Hoechst 33342 (blue). Scale bars 20 μm. Images are representative of two to three independent experiments.

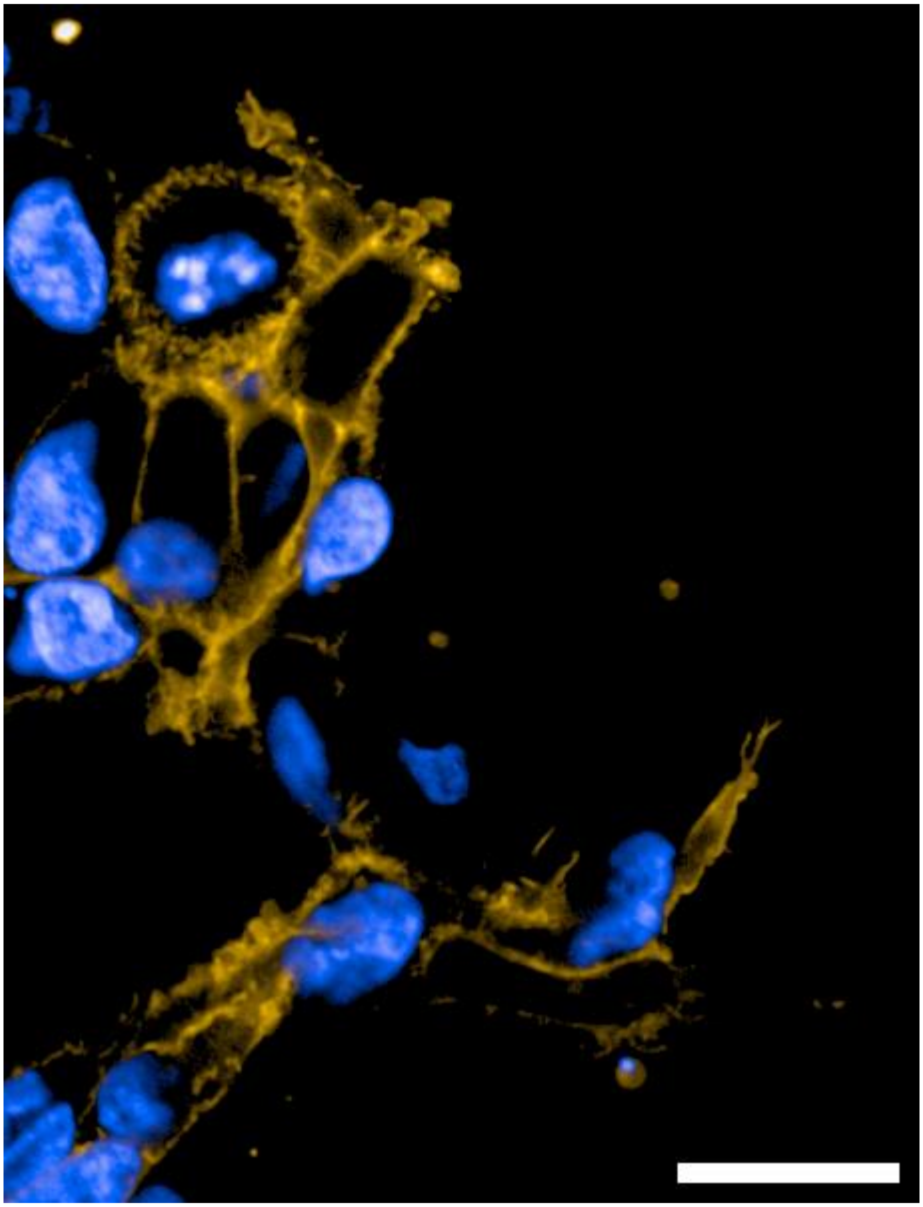

Figure S5. Live cell confocal imaging of induced CB_1R-HEK293TR cells (CB_1R-expression) incubated with **29** (250 nM, yellow) at 63x magnification. Images recorded after 1 min. Nucleus counter stain: Hoechst 33342 (blue). Scale bars 20 μm.

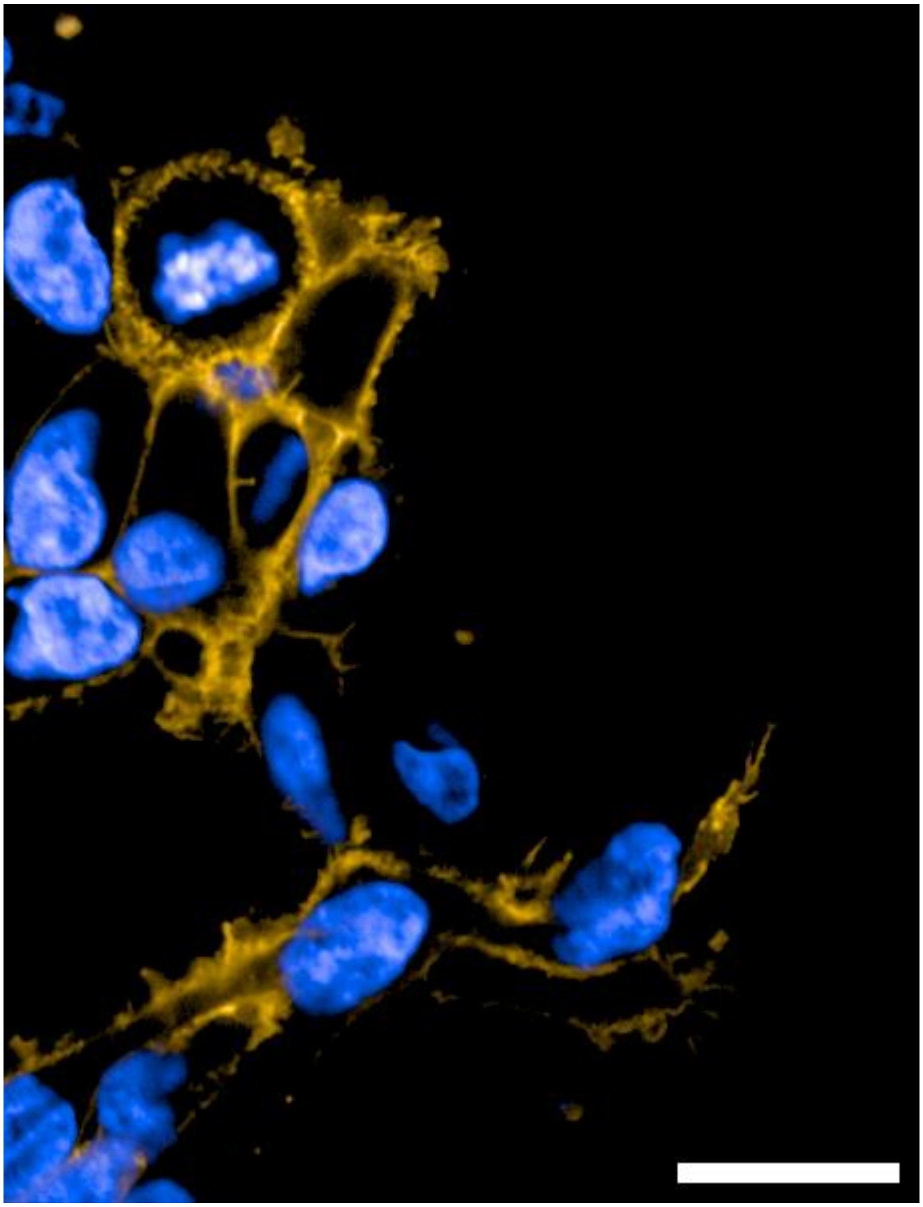

Figure S6. Live cell confocal imaging of induced CB_1R-HEK293TR cells (CB_1R-expression) incubated with **29** (250 nM, yellow) at 63x magnification. Images recorded after 3 min. Nucleus counter stain: Hoechst 33342 (blue). Scale bars 20 µm.

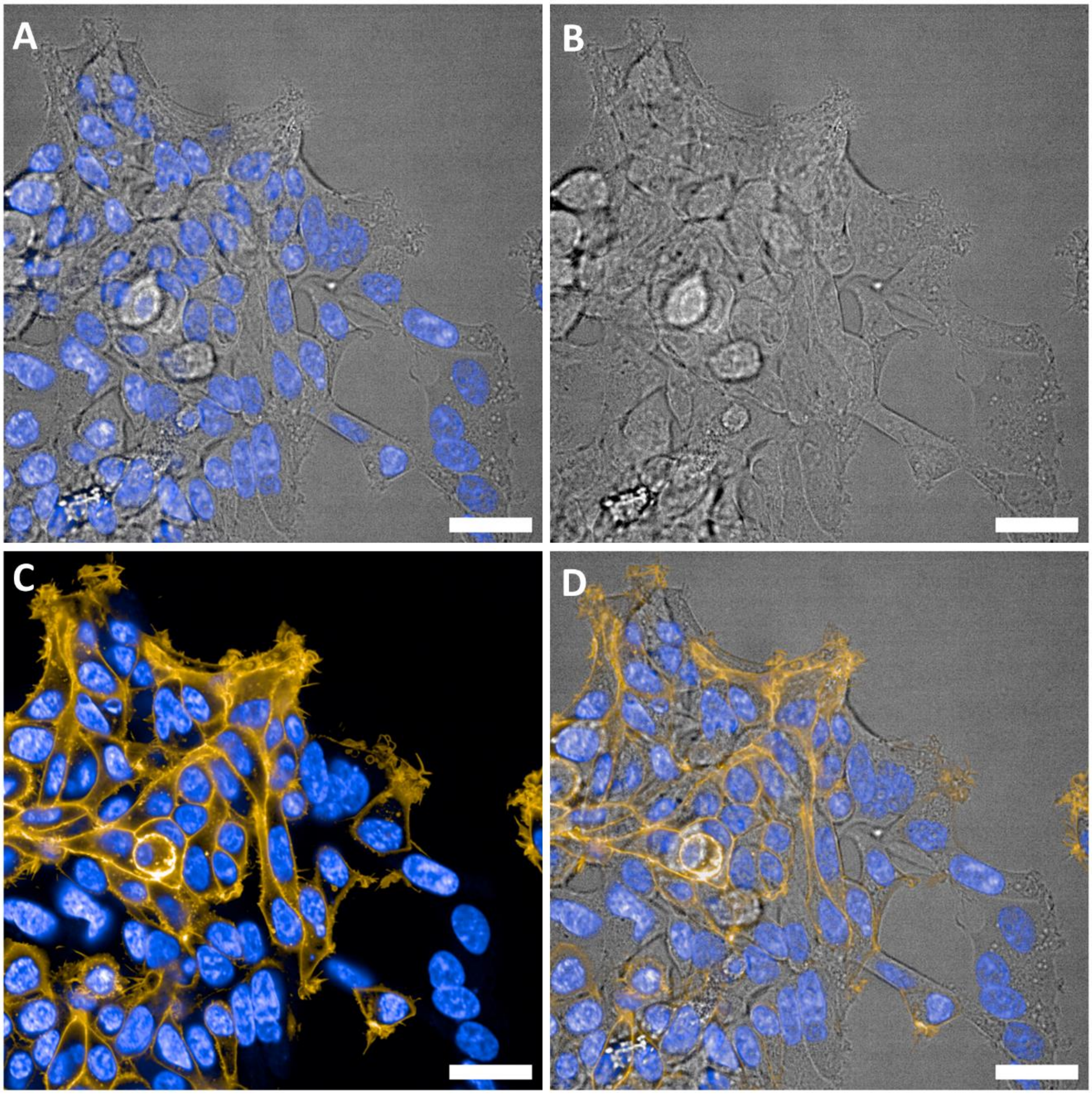

Figure S7. Live cell imaging of induced CB_1R-HEK293TR cells (CB_1R-expression). A) Merged brightfield image with nucleus counter stain Hoechst 33342 (blue). B) Brightfield image. C) Hoechst 33342 and **29** fluorescent staining (250 nM, yellow). D) Merged image of brightfield image with fluorescent staining of Hoechst 33342 and **29**. Images recorded at 63x magnification. Scale bars 20 μm.

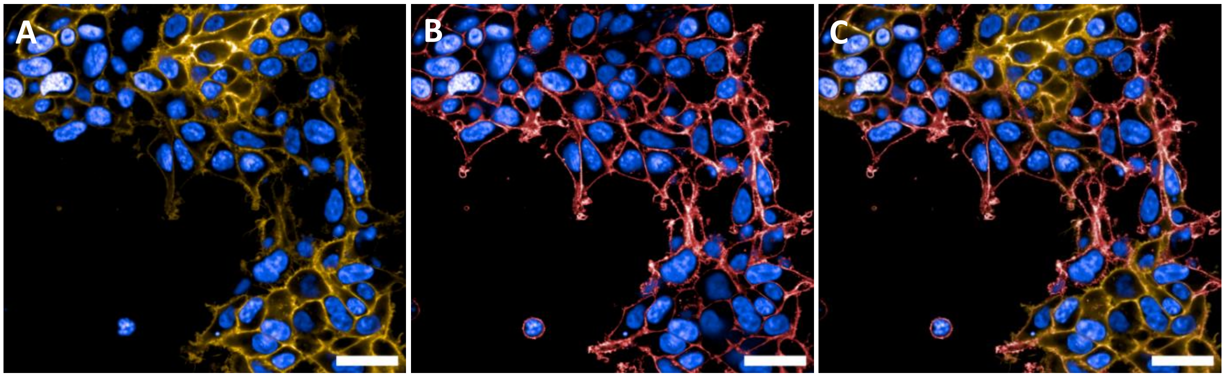

Figure S8. Confocal live cell imaging of induced CB_1R-HEK293TR cells (CB_1R-expression) with plasma membrane stain. A) Fluorescent imaging with **29** (250 nM, yellow) and nucleus counter stain Hoechst 33342 (blue). B) Fluorescent imaging with Cell Mask Deep Red (0.8 µg/mL, 60 min) and nucleus counter stain Hoechst 33342 (blue). C) Fluorescent imaging with Cell Mask Deep Red, nucleus counter stain Hoechst 33342 (blue) and **29** (250 nM, yellow). Images recorded at 63x magnification. Scale bars 20 µm.

Computational Docking

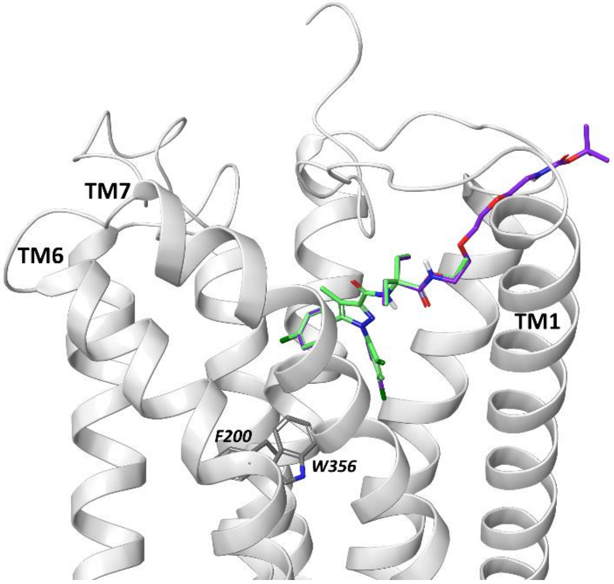

Figure S9. CB_1R X-ray structure (PDB ID: 5TGZ in light gray) with docked pyrazole ligand **15** (green) and pyrazole probe Boc precursor **23b** (purple). Both compounds adopt the same binding poses. Linker of **23b** exits the binding pocket between transmembrane helix TM1 and 2 reaching to the receptor surface. Protein is represented as ribbon-cartoon. Ligands and CB_1R toggle switch F200 and W356 side chains are represented as sticks.

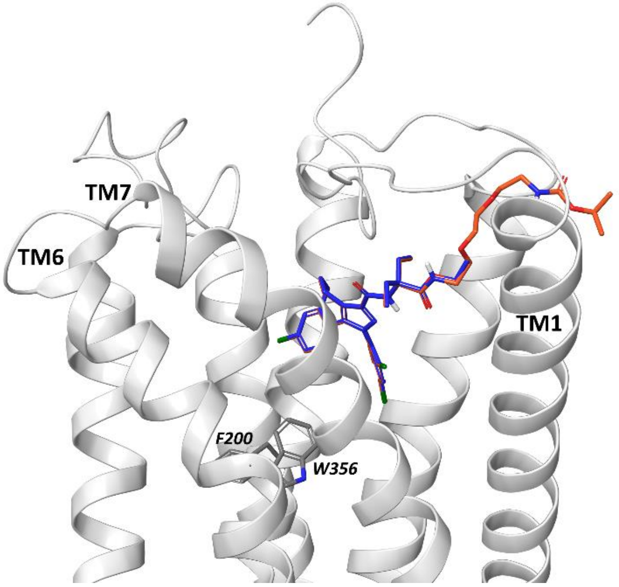

Figure S10. CB_1R X-ray structure (PDB ID: 5TGZ in light gray) with docked tricyclic pyrazole ligand **16** (blue) and tricyclic pyrazole probe Boc precursor **24** (red). Both compounds adopt the same binding poses. Linker of **24** exits the binding pocket between transmembrane helix TM1 and 2 reaching to the receptor surface. Protein is represented as ribbon-cartoon. Ligands and CB_1R toggle switch F200 and W356 side chains are represented as sticks.

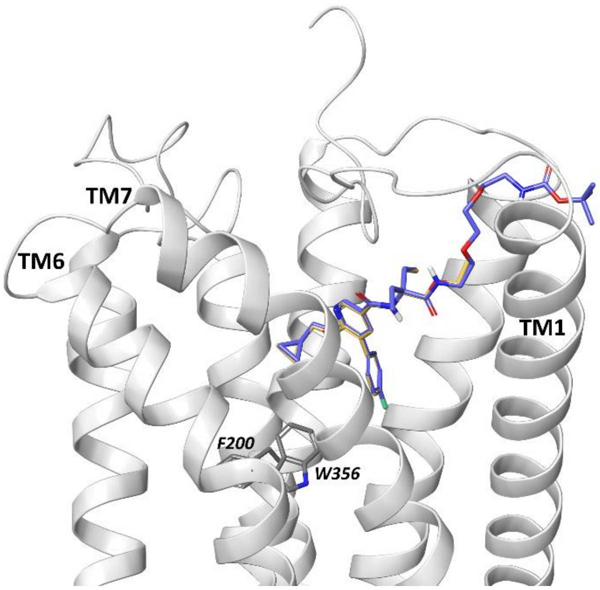

Figure S11. CB_1R X-ray structure (PDB ID: 5TGZ in light gray) with docked pyridine ligand **17** (beige) and pyridine probe Boc precursor **25** (dark blue). Both compounds adopt the same binding poses. Linker of **25** exits the binding pocket between transmembrane helix TM1 and 2 reaching to the receptor surface. Protein is represented as ribbon-cartoon. Ligands and CB_1R toggle switch F200 and W356 side chains are represented as sticks.

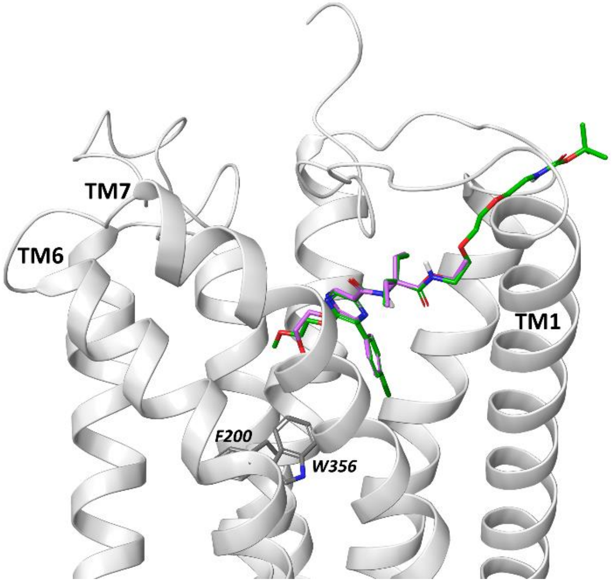

Figure S12. CB_1R X-ray structure (PDB ID: 5TGZ in light gray) with docked pyrazine ligand **18** (purple) and pyrazine probe Boc precursor **26** (green). Both compounds adopt comparable binding poses. Linker of **26** exits the binding pocket between transmembrane helix TM1 and 2 reaching to the receptor surface. Protein is represented as ribbon-cartoon. Ligands and CB_1R toggle switch F200 and W356 side chains are represented as sticks.

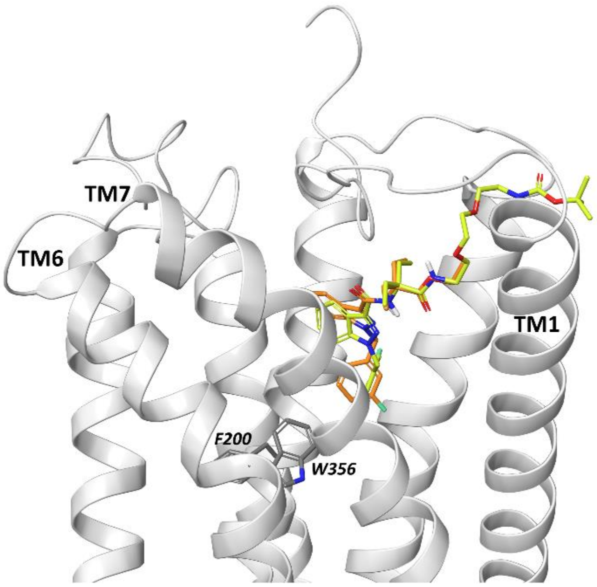

Figure S13. CB_1R X-ray structure (PDB ID: 5TGZ in light gray) with docked indazole ligand **19** (orange) and indazole probe Boc precursor **27** (lime). Both compounds adopt comparable binding poses. Linker of **27** exits the binding pocket between transmembrane helix TM1 and 2 reaching to the receptor surface. Protein is represented as ribbon-cartoon. Ligands and CB_1R toggle switch F200 and W356 side chains are represented as sticks.

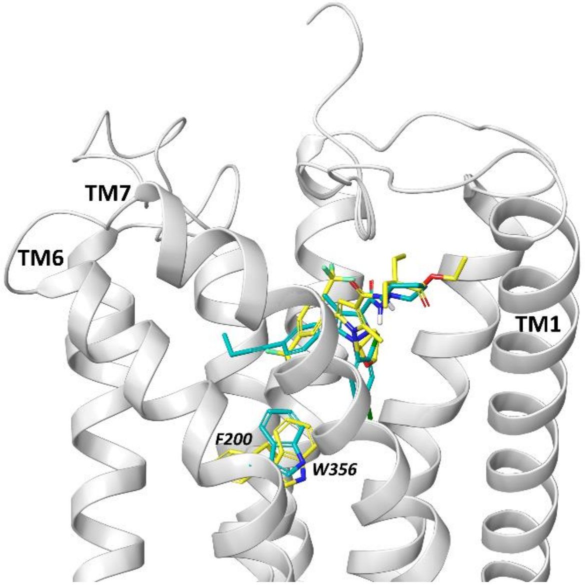

Figure S14. CB_1R X-ray structure (PDB ID: 5TGZ in light gray) with co-crystallized ligand AM6538 (turquois) and superimposed ligand **14**. Orientation of AM6538 aligns with the orientation of ligand **14** (yellow). Piperidine ring of AM6538 and DEG unit of **14** points towards extracellular space. The heterocyclic pharmacophore of AM6538 is oriented similar as pyrazole core in **14**.

Conformational Molecular Dynamics (MD) Simulations

MD simulations were carried out via Desmond v5.1 in Maestro Schrödinger version 13.2.128 (2022-2 release) on the basis of the work from David et al.[1]

The described structures were prepared via LigPrep software, minimized with the OPLS4 force field, and ionized according to Epik at pH 7. The MD simulation solvent systems were set up as a cubic simulation box with a buffer size of 20 Å to prevent the ligand from interacting with its own image. For simulations in water, the TIP3P model was used, and for simulations in *n*-octane, a custom solvent box was constructed as recommended by Schrödinger. All simulations were performed using the OPLS4 force field. Desmond MD simulation was submitted with default parameters for 50 ns, saving a frame every 0.1 ns, resulting in 500 conformers for each structure-solvent-combination. For each conformer, intramolecular hydrogen bonds were detected and exported along with three-dimensional PSA values. Data was analyzed with GraphPad Prism 10.1 software.

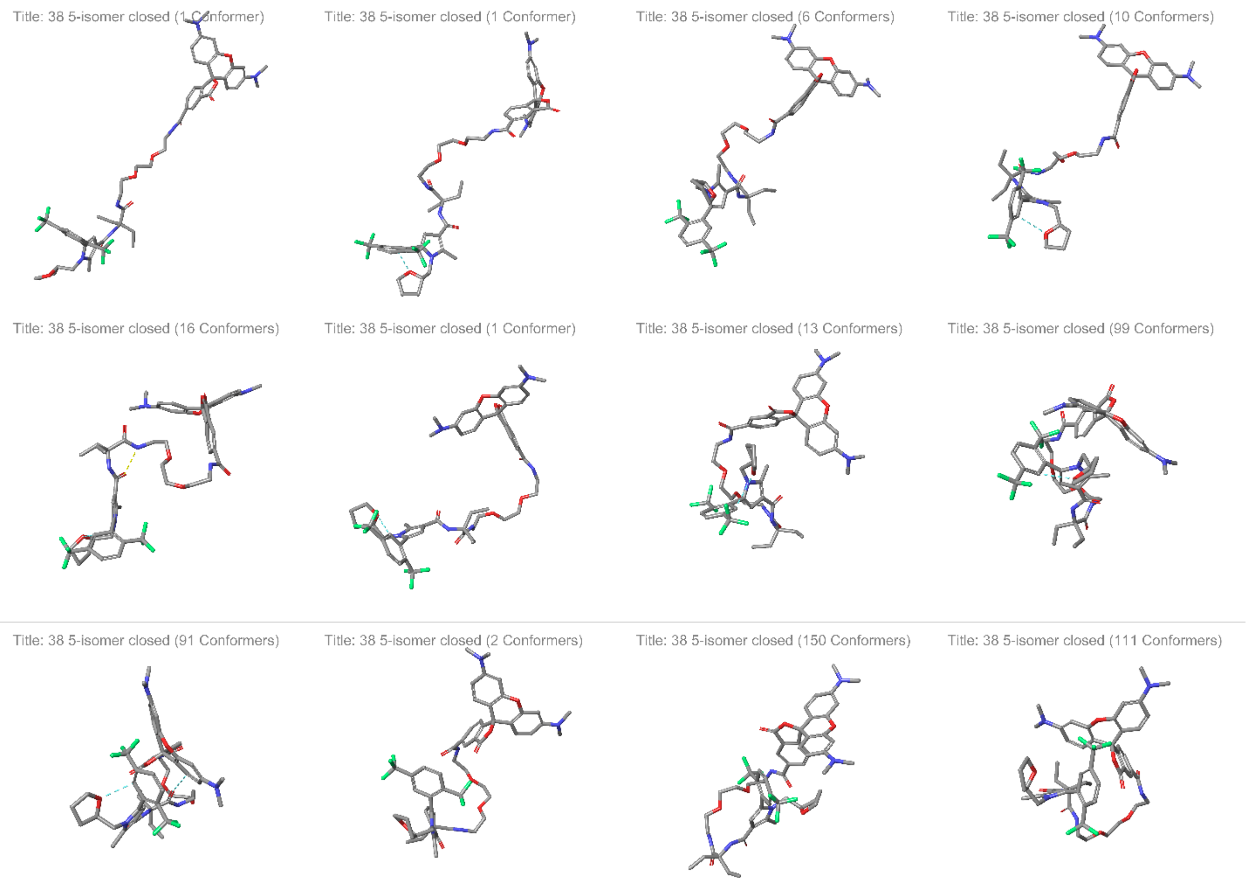

Figure S15. Representative conformers of **29** (5-isomer, closed) observed during 50 ns MD simulation in water. All 500 conformers were clustered based on atomic RMSD. For each of the twelve clusters a representative conformer is depicted. Grey atoms: C, Red atoms: O, Blue atoms: N, Green atoms: F, yellow dashed line: intramolecular hydrogen bond, light blue dashed line: aromatic H-bond.

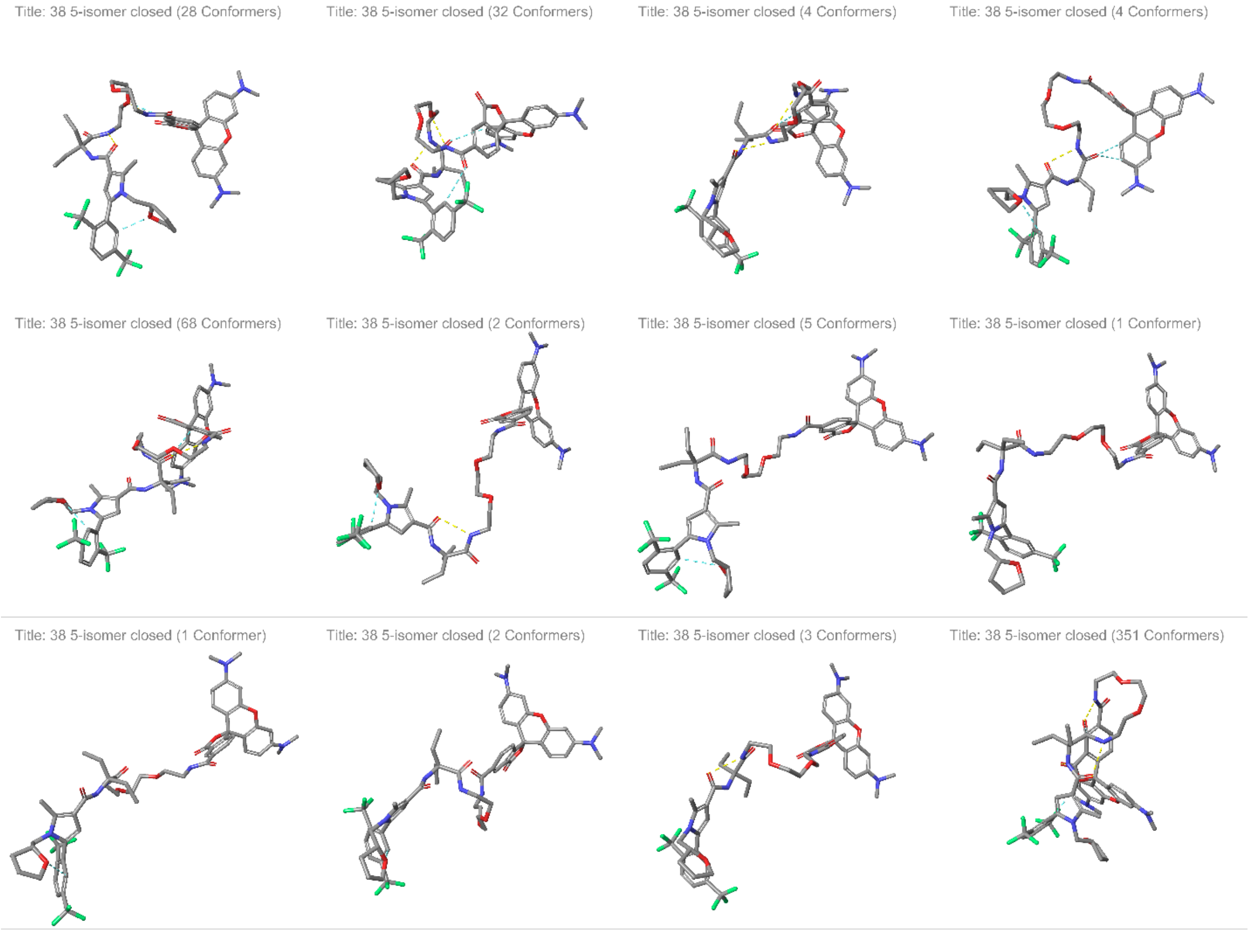

Figure S16. Representative conformers of **29** (5-isomer, closed) observed during 50 ns MD simulation in *n*-octane. All 500 conformers were clustered based on atomic RMSD. For each of the twelve clusters a representative conformer is depicted. Grey atoms: C, Red atoms: O, Blue atoms: N, Green atoms: F, yellow dashed line: intramolecular hydrogen bond, light blue dashed line: aromatic H-bond.

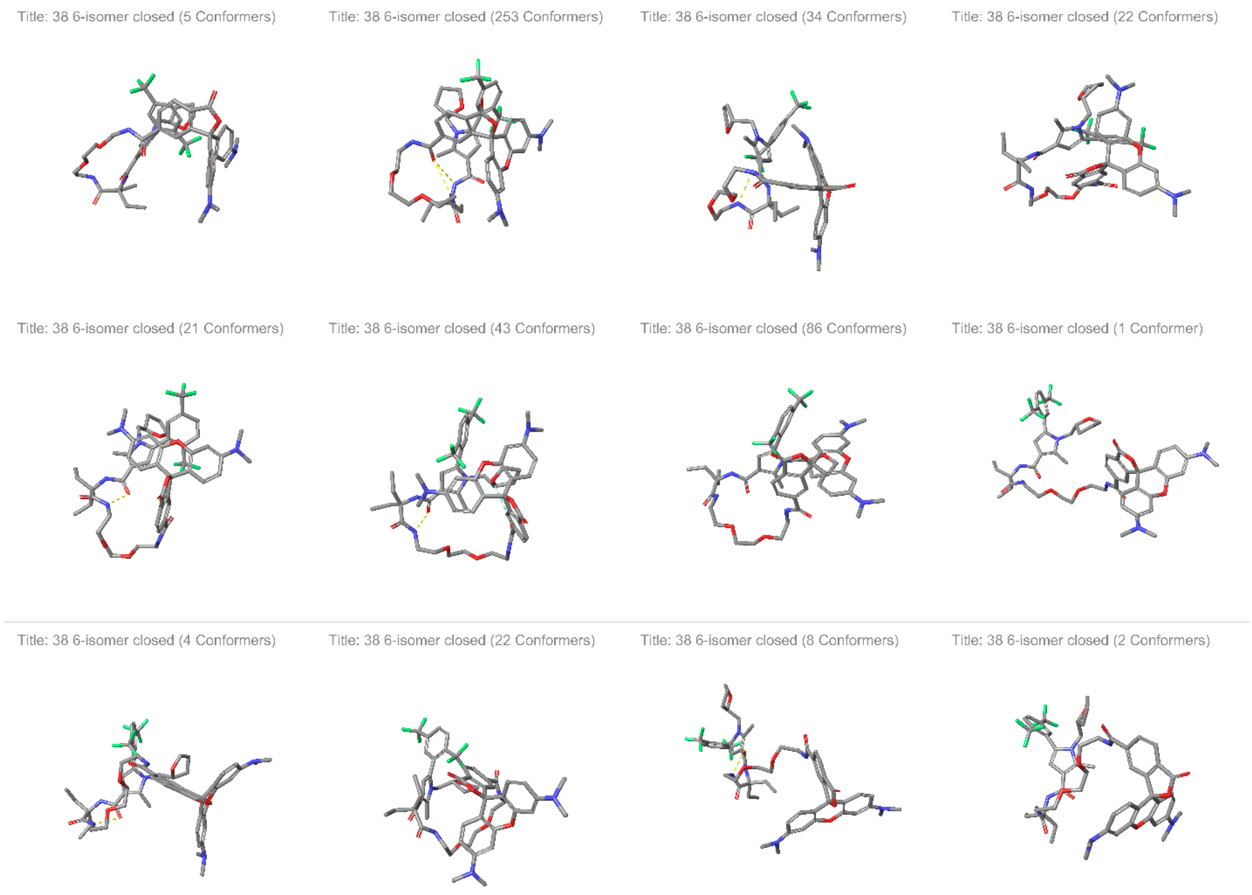

Figure S17. Representative conformers of **29** (6-isomer, closed) observed during 50 ns MD simulation in water. All 500 conformers were clustered based on atomic RMSD. For each of the twelve clusters a representative conformer is depicted. Grey atoms: C, Red atoms: O, Blue atoms: N, Green atoms: F, yellow dashed line: intramolecular hydrogen bond, light blue dashed line: aromatic H-bond.

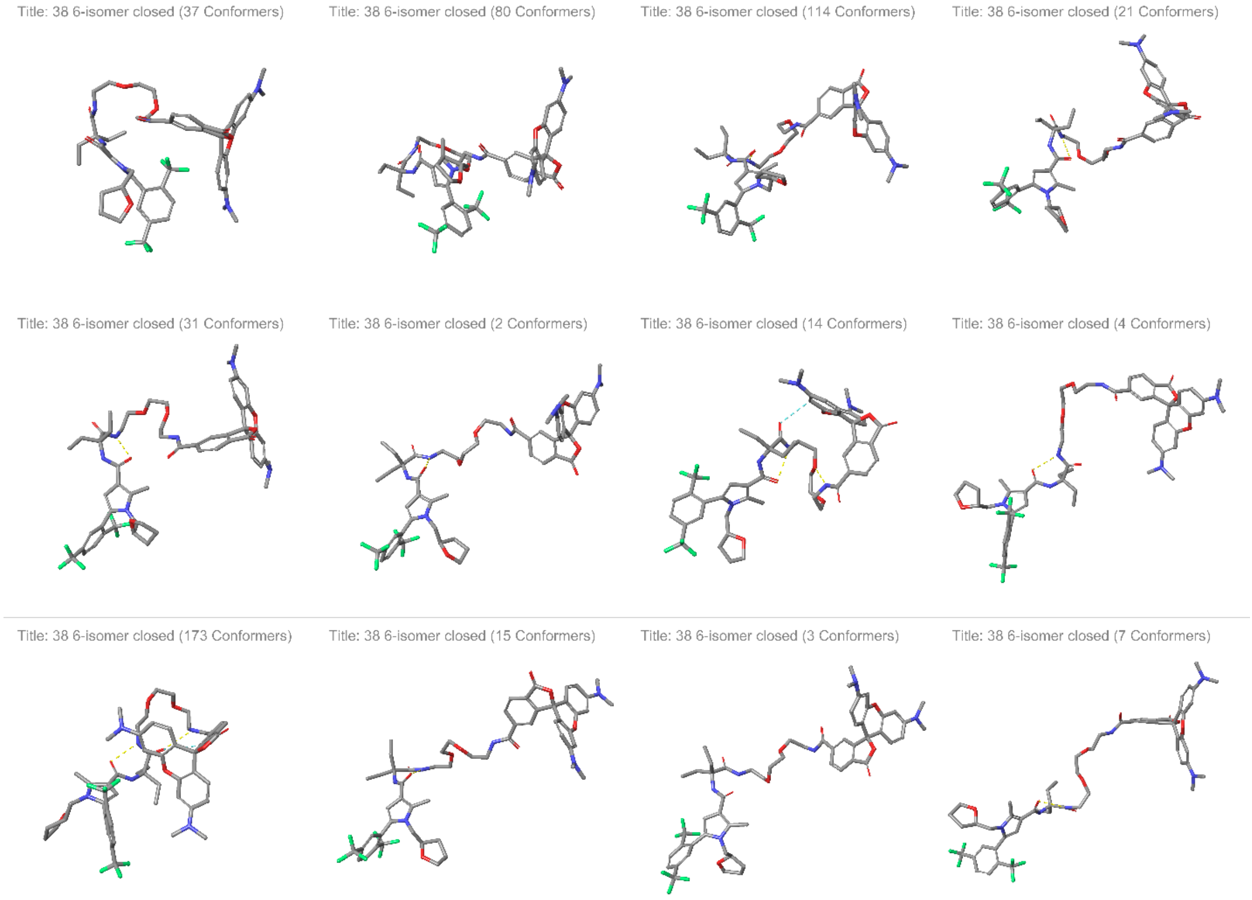

Figure S18. Representative conformers of **29** (6-isomer, closed) observed during 50 ns MD simulation in *n*-octane. All 500 conformers were clustered based on atomic RMSD. For each of the twelve clusters a representative conformer is depicted. Grey atoms: C, Red atoms: O, Blue atoms: N, Green atoms: F, yellow dashed line: intramolecular hydrogen bond, light blue dashed line: aromatic H-bond.

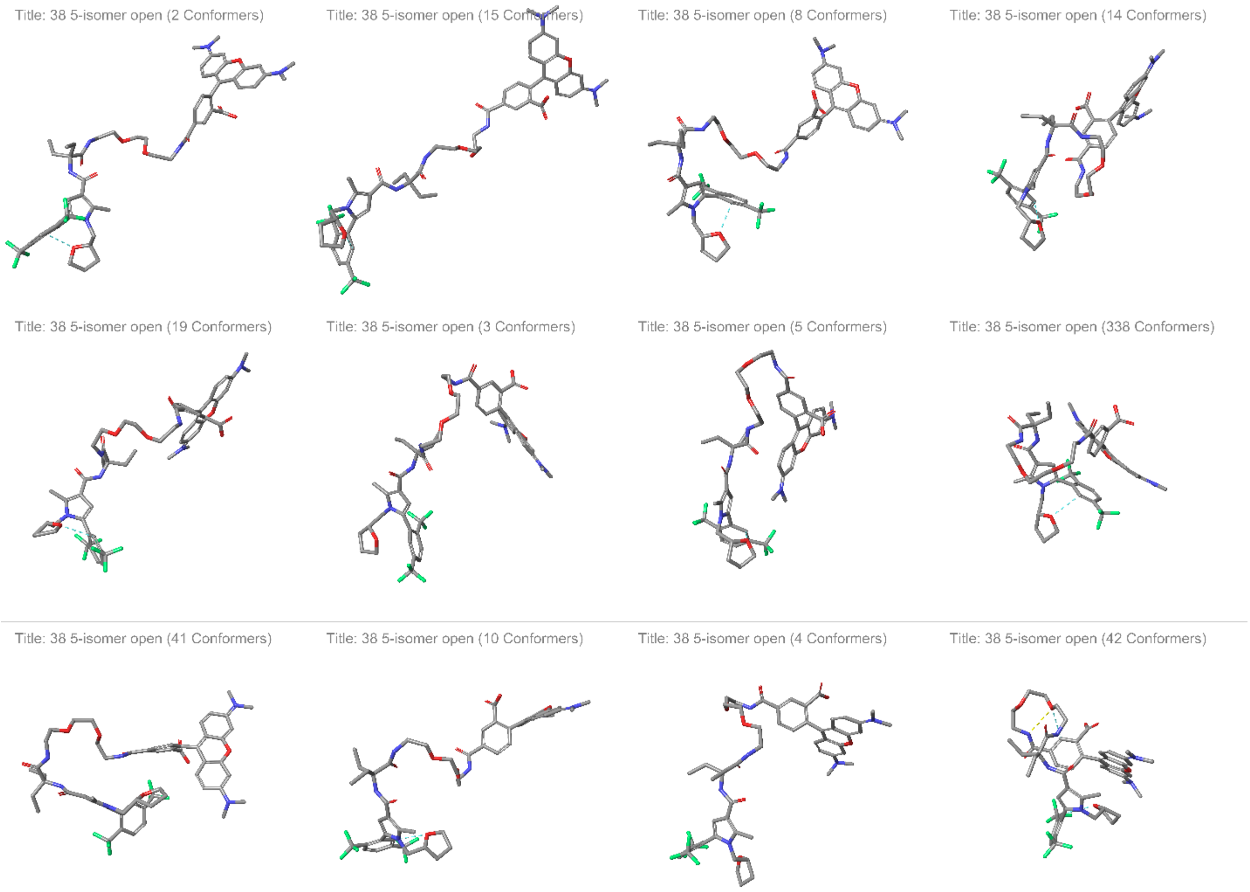

Figure S19. Representative conformers of **29** (5-isomer, open) observed during 50 ns MD simulation in water. All 500 conformers were clustered based on atomic RMSD. For each of the twelve clusters a representative conformer is depicted. Grey atoms: C, Red atoms: O, Blue atoms: N, Green atoms: F, yellow dashed line: intramolecular hydrogen bond, light blue dashed line: aromatic H-bond.

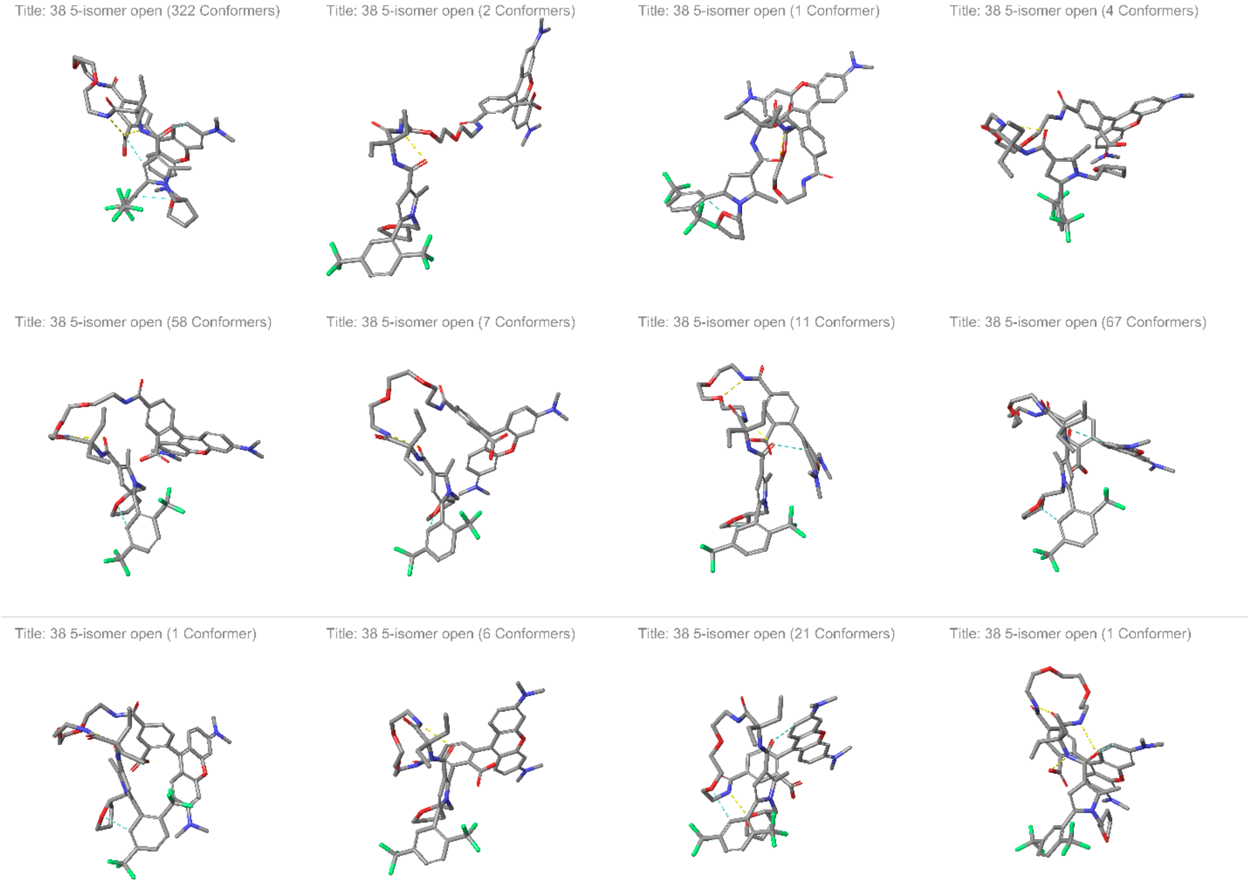

Figure S20. Representative conformers of **29** (5-isomer, open) observed during 50 ns MD simulation in *n*-octane. All 500 conformers were clustered based on atomic RMSD. For each of the twelve clusters a representative conformer is depicted. Grey atoms: C, Red atoms: O, Blue atoms: N, Green atoms: F, yellow dashed line: intramolecular hydrogen bond, light blue dashed line: aromatic H-bond.

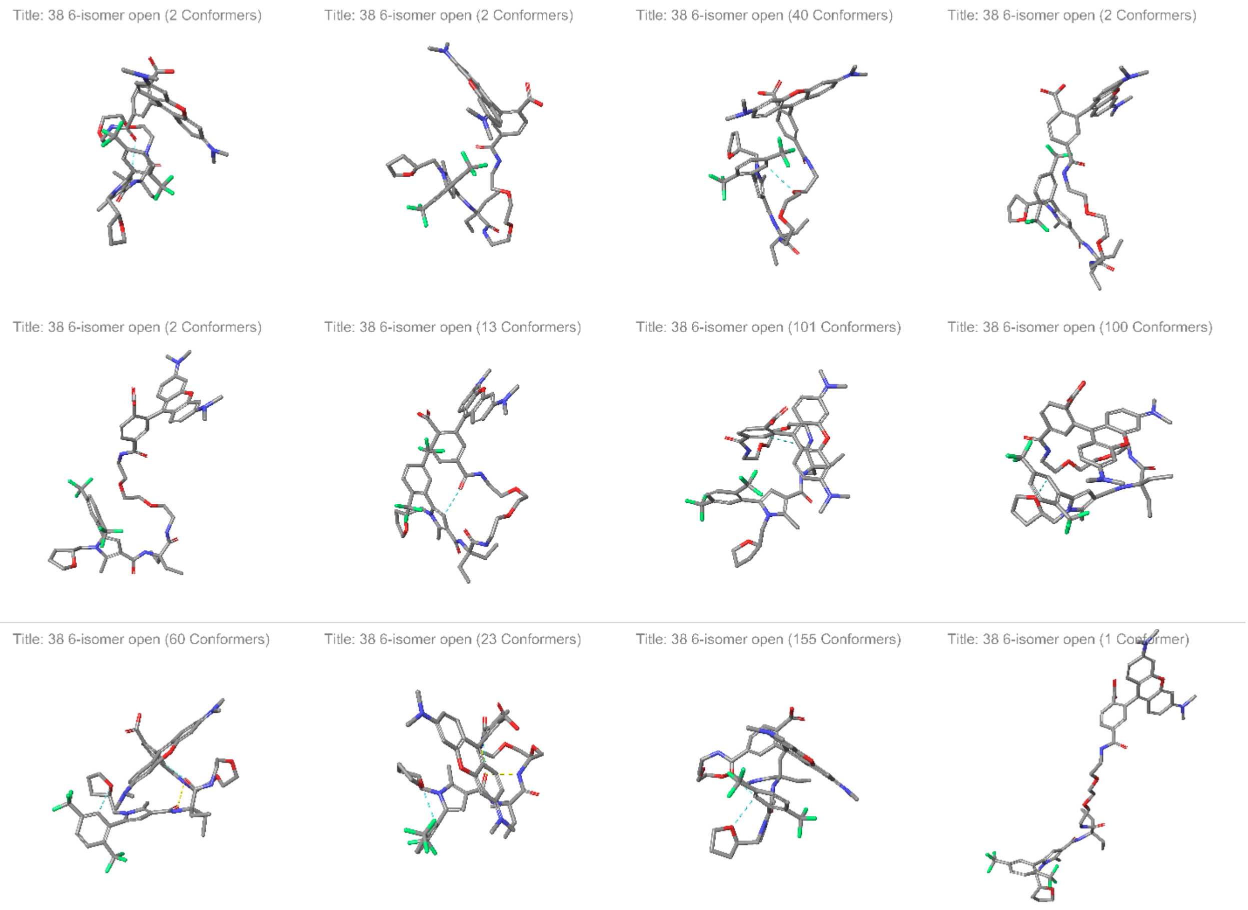

Figure S21. Representative conformers of **29** (6-isomer, open) observed during 50 ns MD simulation in water. All 500 conformers were clustered based on atomic RMSD. For each of the twelve clusters a representative conformer is depicted. Grey atoms: C, Red atoms: O, Blue atoms: N, Green atoms: F, yellow dashed line: intramolecular hydrogen bond, light blue dashed line: aromatic H-bond.

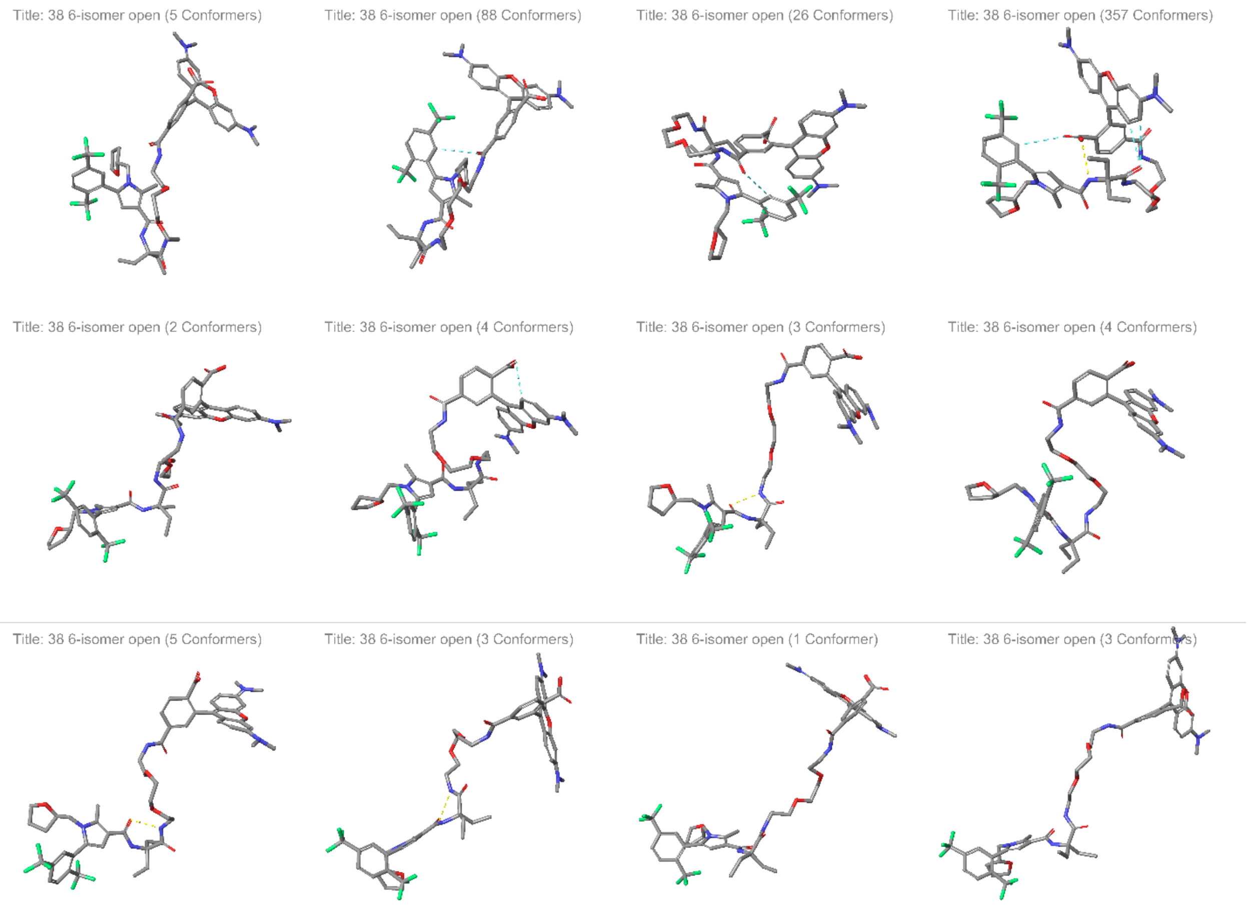

Figure S22. Representative conformers of **29** (6-isomer, open) observed during 50 ns MD simulation in *n*-octane. All 500 conformers were clustered based on atomic RMSD. For each of the twelve clusters a representative conformer is depicted. Grey atoms: C, Red atoms: O, Blue atoms: N, Green atoms: F, yellow dashed line: intramolecular hydrogen bond, light blue dashed line: aromatic H-bond.

Table S4. Calculated mean 3D PSA and mean intramolecular hydrogen bonds (IMHB).

**Compound 29**	**Mean 3D PSA (Å^2)**		**Mean IMHB count**	
	**H_2O**	***n*-octane**	**H_2O**	***n*-octane**
5-isomer spirolactone	141.5	99.8	0.2	2.2
6-isomer spirolactone	132.2	142.7	1	1.2
5-isomer zwitterion	171.7	115.4	0.1	1.7
6-isomer zwitterion	145.5	126.6	0.4	1.6

Photophysical Characterization

Table S5. Absorbance/Emission spectra of fluoroprobes (PBS pH 7.4).

Compound	Dye	Abs/Em (nm)	QY
**28**	NBD	475/550	1.30%
**29**	TAMRA	555/585	36.60%
**30**	NBD	475/570	0.80%
**31**	TAMRA	520/585	17.50%
**32**	NBD	485/570	1.30%
**33**	TAMRA	520/585	11.10%
**40**	NBD	465/550	1.30%
**41**	TAMRA	560/585	47.40%
**34**	NBD	480/545	2.00%
**35**	TAMRA	560/590	56.20%
**36**	NBD	475/565	1.50%
**37**	TAMRA	555/585	49.00%
**51**	S-NBD	465/550	0.30%
**52**	N-NBD	425/495	0.10%

NMR spectra

Coalescence effect of **24** observed for bridging CH_2-groups in ^{1}H-NMR (MeOD). The protons of H_b and H_c were not detectable by ^{1}H-NMR at 295 K due to conformational flexibility of the 1,4,5,6-tetrahydrobenzo[6,7]cyclohepta[1,2-*c*]pyrazole substructure. ^{1}H NMR was recorded at lower temperatures, to detect the two possible atropisomer forms of **24**. This behavior was reported previously for a related structure.[2]

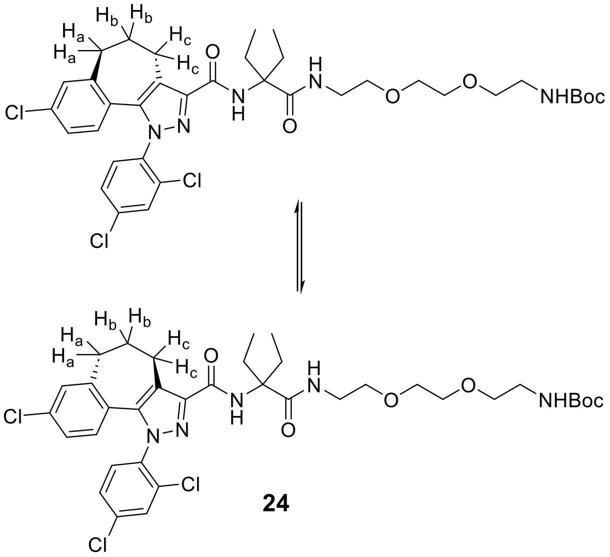

24

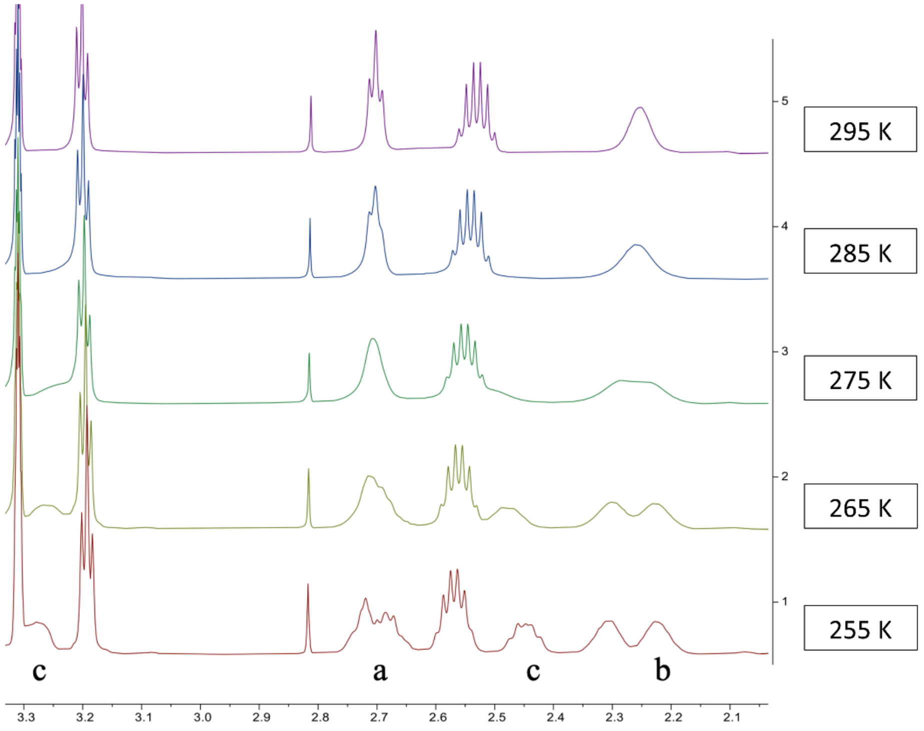

Figure S23. Comparison of ^{1}H NMR spectra of compound **24** at different temperatures. A coalescence effect was observed when temperature was decreased from 295 K to 255 K in 10 K steps.[2]

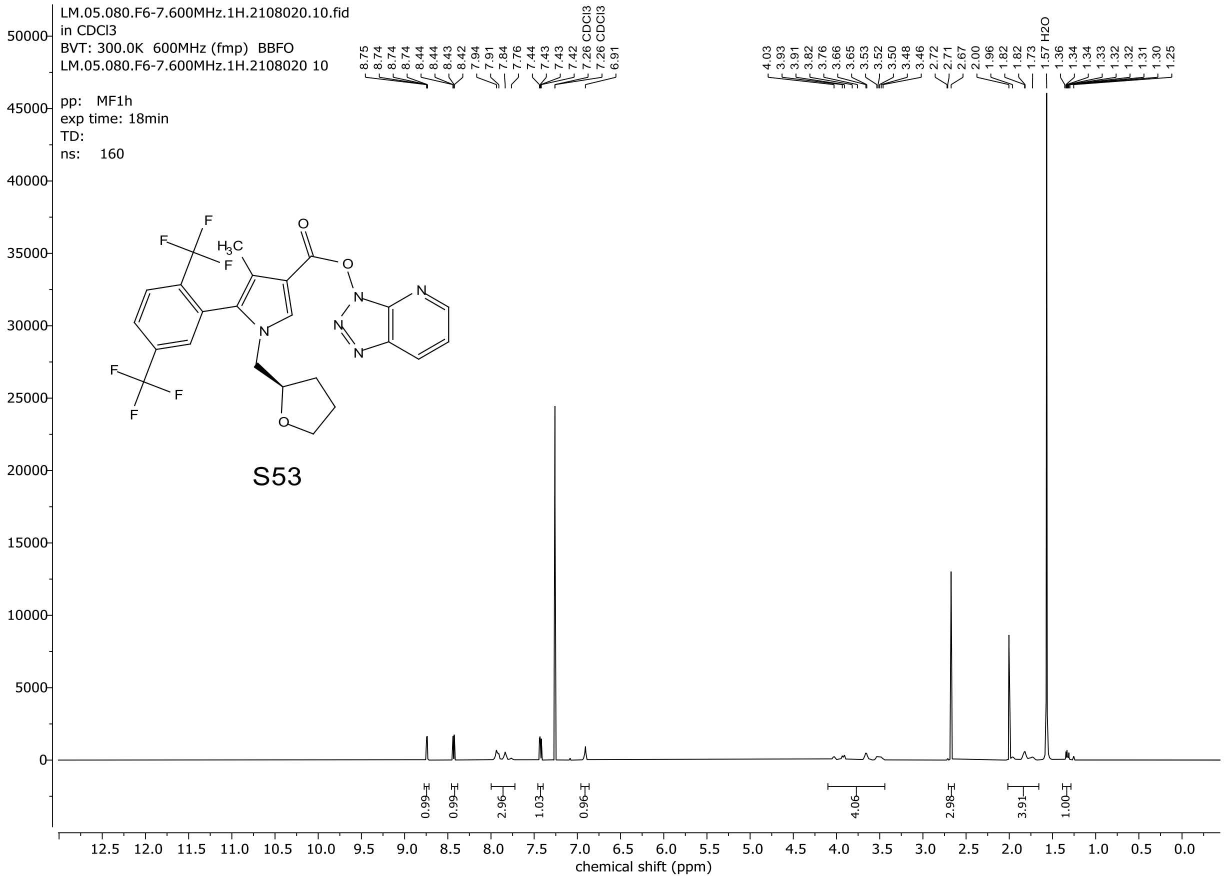

Figure S24. ^{1}H-NMR spectrum ($CDCl_3$) of side product **S53**.

HPLC purity

HPLC traces of final probes measured on an Agilent 1260 series HPLC system employing a DAD detector (254 nm) equipped with Agilent Technologies 6120 Quadrupole LC/MS in electrospray positive and negative ionization modes (ESI-MS). A Thermo Accuore RP-MS (30 × 2.1 mm, 2.6 µm) column was used with a flow rate 0.8 mL/min in combination with the following separation conditions: 0.1% formic acid in water (solvent A); 0.1% formic acid in ACN (solvent B); System (1) 5% B for 0.5 min, from 5 to 95% B in 6.5 min, 95% B for 1 min (stop point at 8 min); System (2) 5% B for 0.2 min, from 5 to 95% B in 0.9 min, 95% B for 1.4 min (stop point at 2.5 min). Data analysis was performed with ChemStation software. The purity of all test compounds was determined to be >95%.

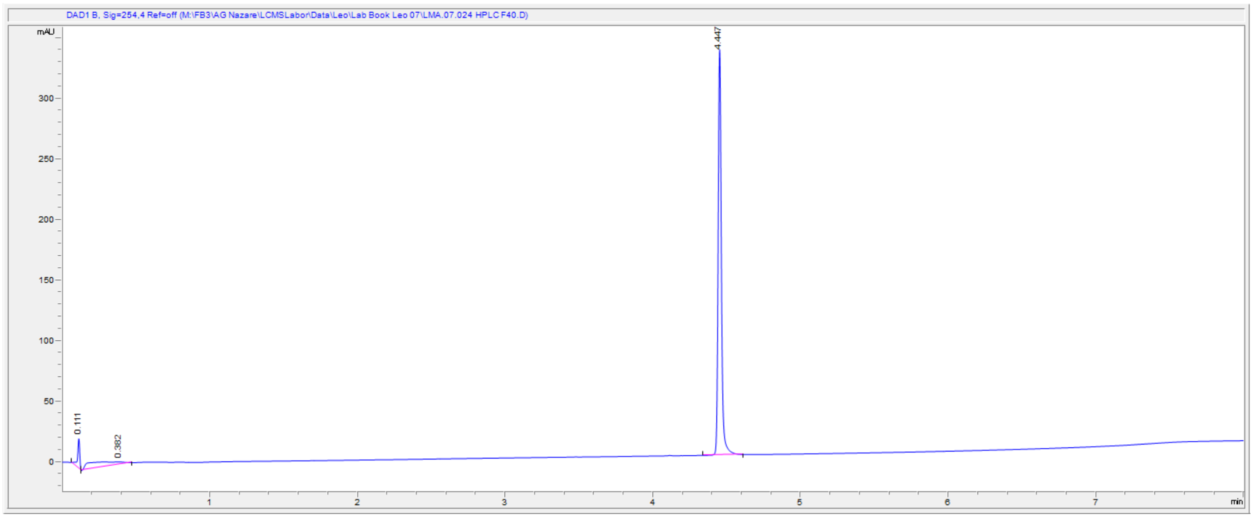

Figure S25. HPLC purity of compound **28**.

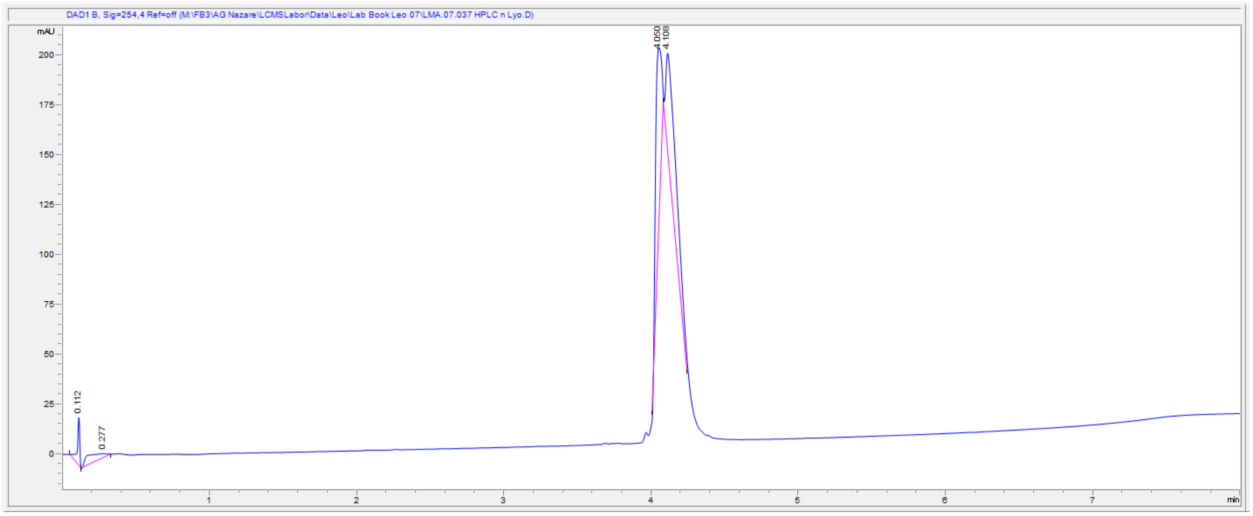

Figure S26. HPLC purity of compound **29** (5/6 TAMRA isomer).

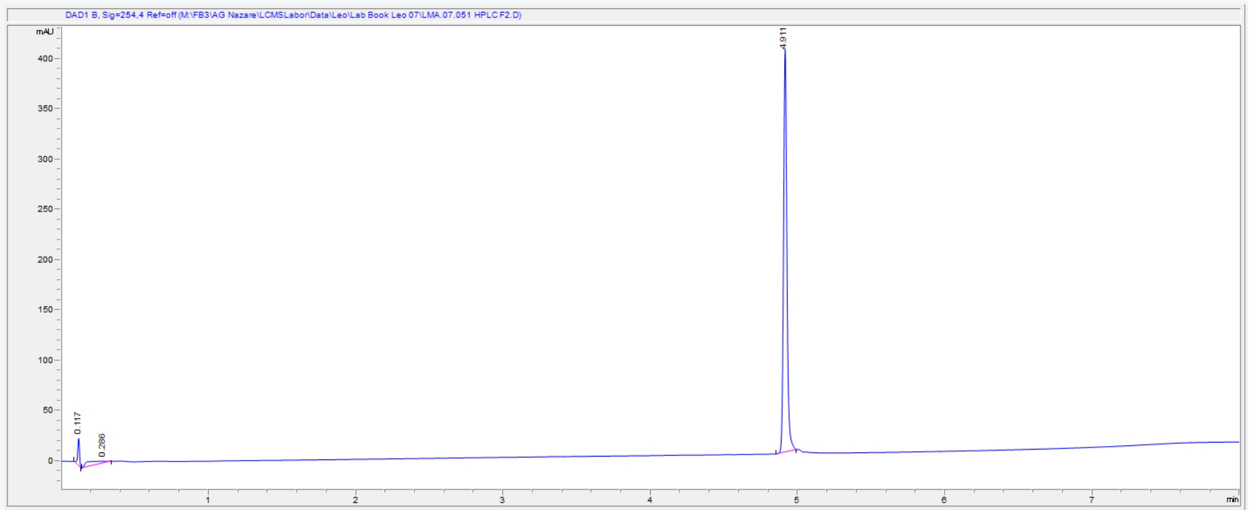

Figure S27. HPLC purity of compound **30**.

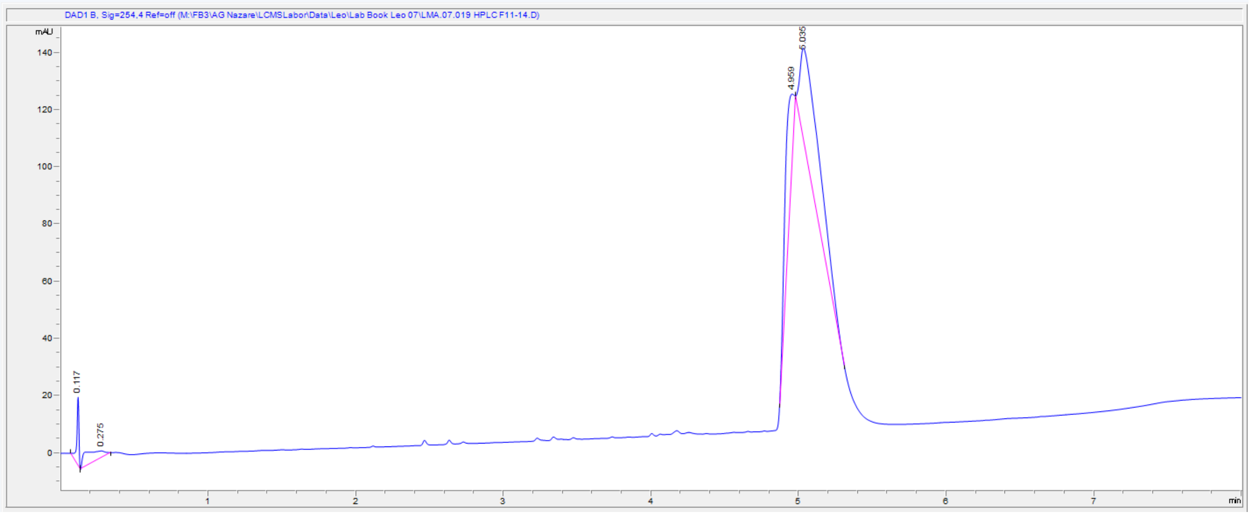

Figure S28. HPLC purity of compound **31** (5/6 TAMRA isomer).

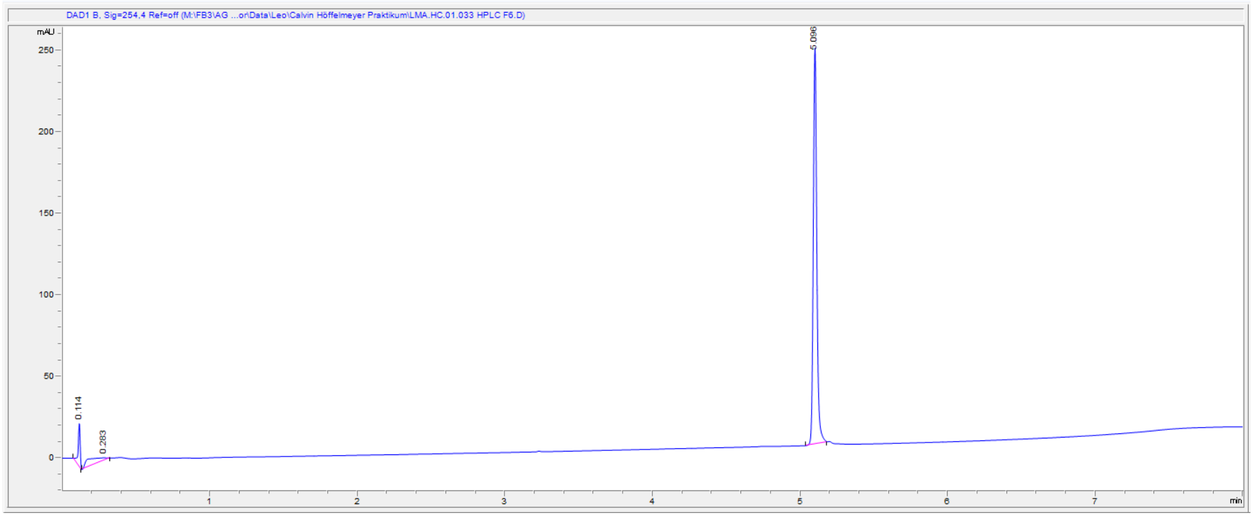

Figure S29. HPLC purity of compound **32**.

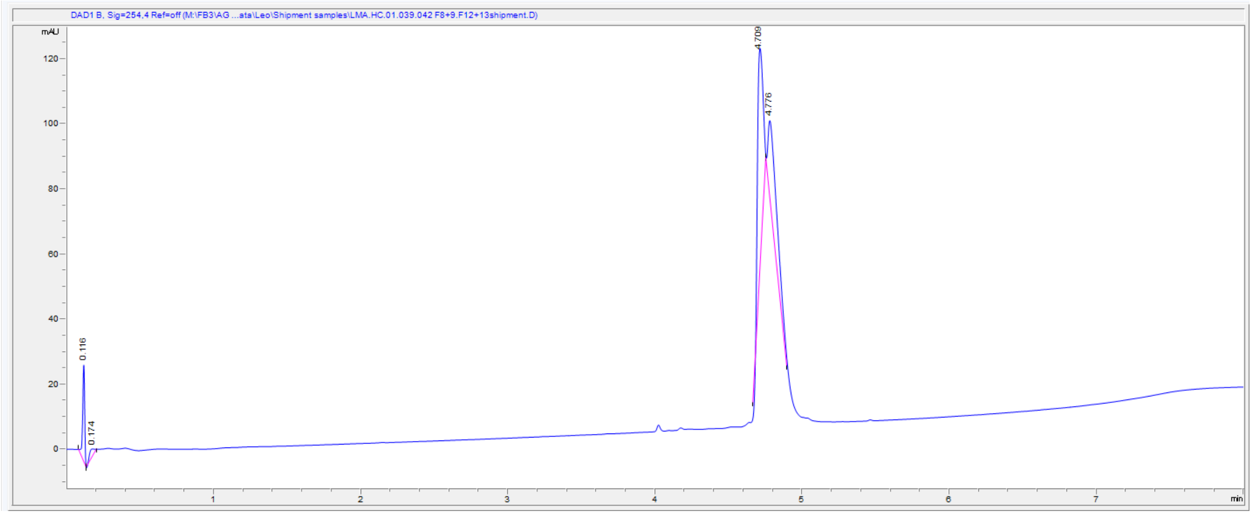

Figure S30. HPLC purity of compound **33** (5/6 TAMRA isomer).

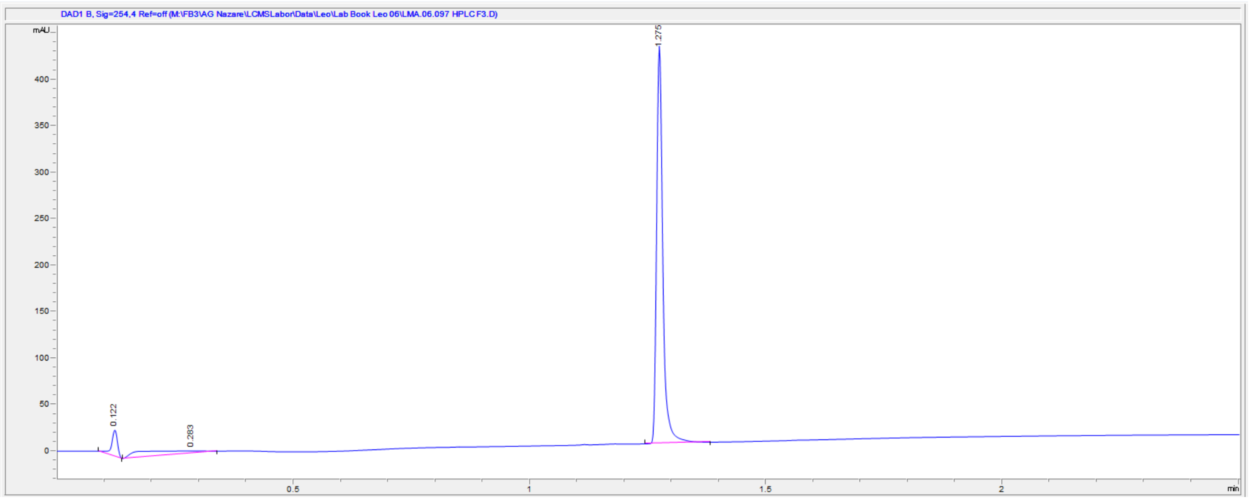

Figure S31. HPLC purity of compound **34**.

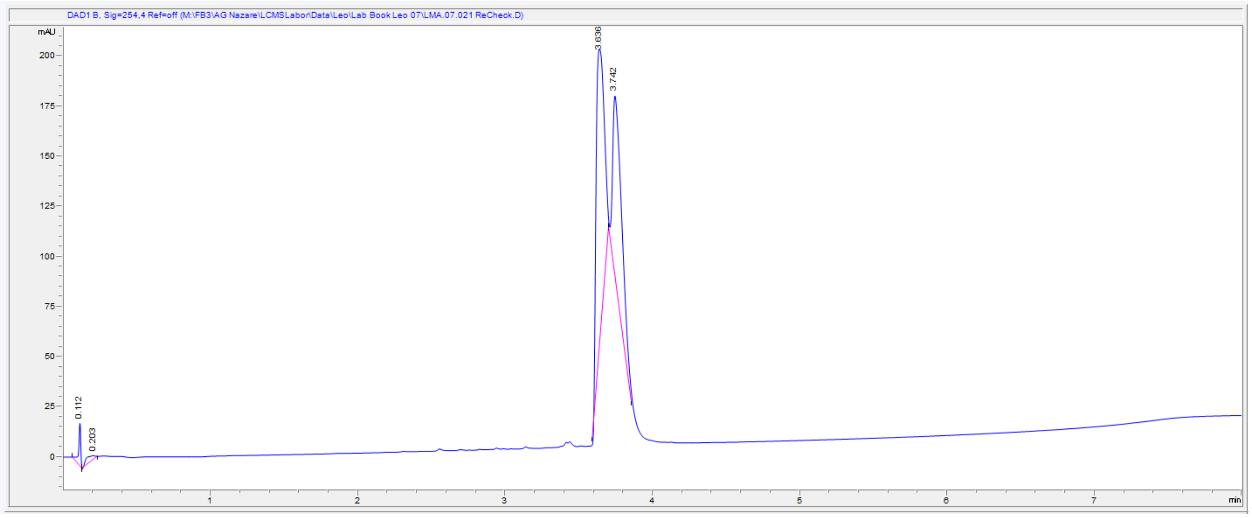

Figure S32. HPLC purity of compound **35** (5/6 TAMRA isomer).

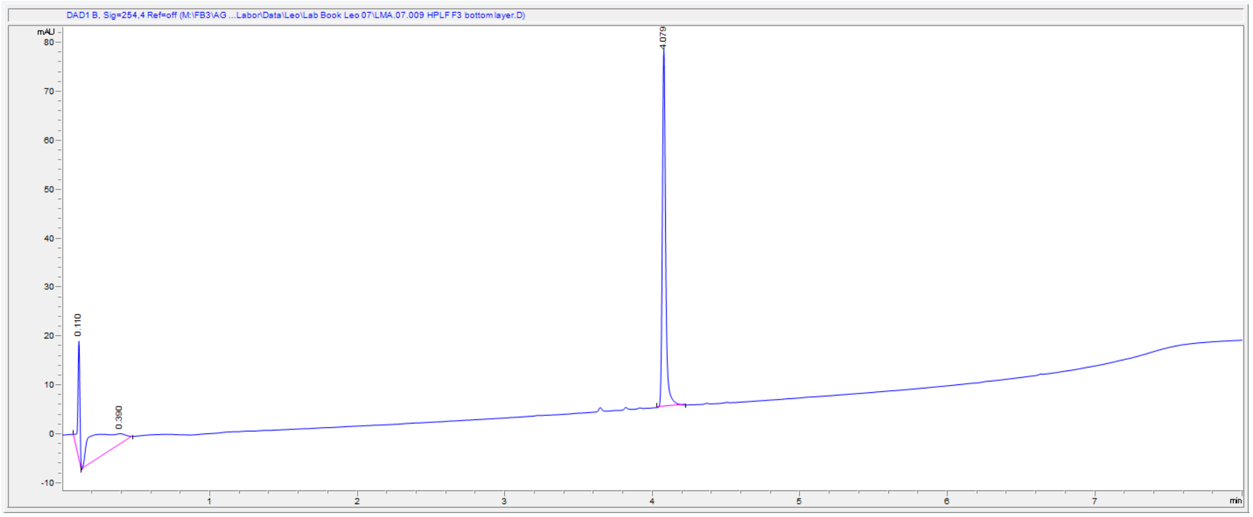

Figure S33. HPLC purity of compound **36**.

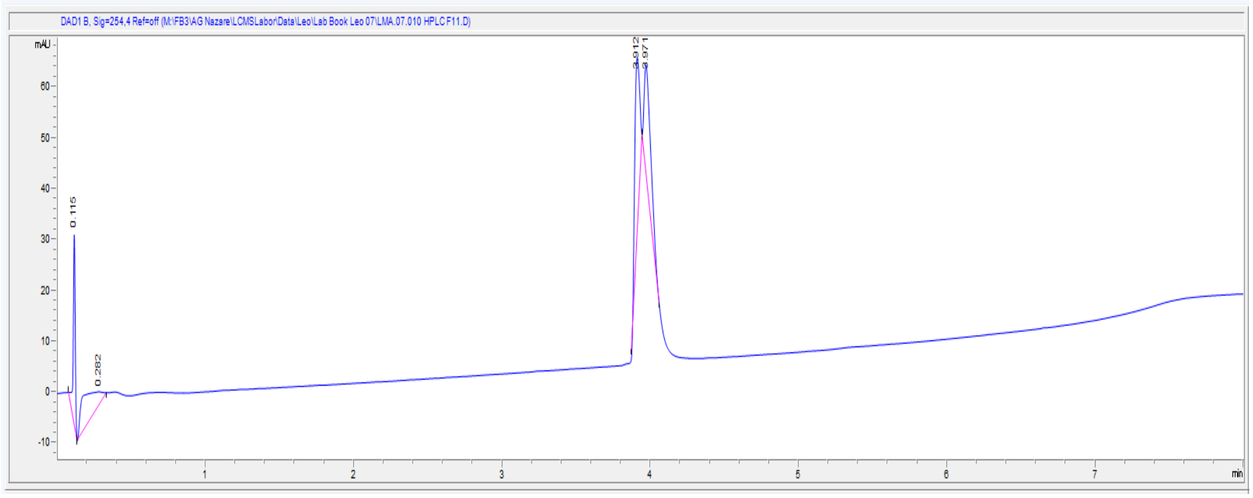

Figure S34. HPLC purity of compound **37** (5/6 TAMRA isomer).

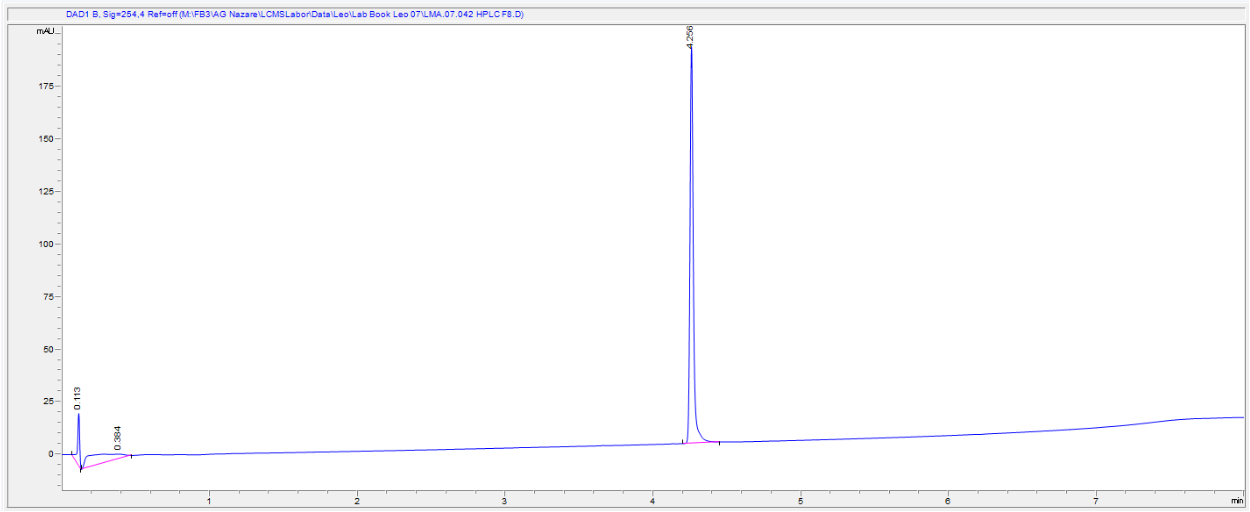

Figure S35. HPLC purity of compound **40**.

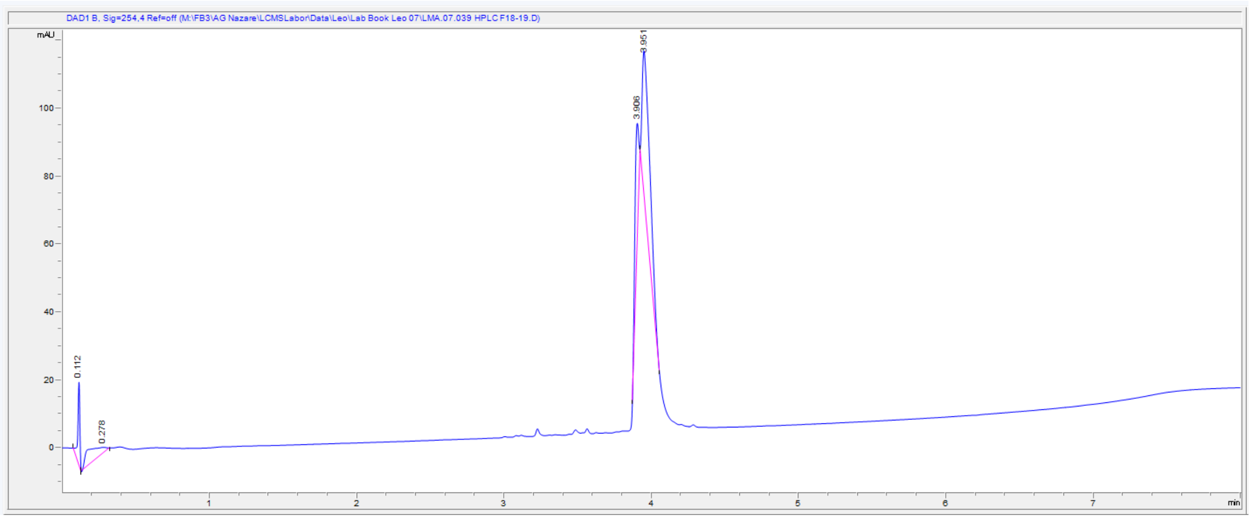

Figure S36. HPLC purity of compound **41** (5/6 TAMRA isomer).

References

(1) David, L.; Wenlock, M.; Barton, P.; Ritzen, A. Prediction of Chameleonic Efficiency. *ChemMedChem* **2021**, *16* (17), 2669-2685.

(2) Zhang, Y.; Burgess, J. P.; Brackeen, M.; Gilliam, A.; Mascarella, S. W.; Page, K.; Seltzman, H. H.; Thomas, B. F. Conformationally constrained analogues of N-(piperidinyl)-5-(4-chlorophenyl)-1-(2,4- dichlorophenyl)-4-methyl-1H-pyrazole-3-carboxamide (SR141716): design, synthesis, computational analysis, and biological evaluations. *J. Med. Chem.* **2008**, *51* (12), 3526-3539.
